# Supplementary material for: Spatial and single-nucleus transcriptomic analysis of genetic and sporadic forms of Alzheimer’s disease
Source: Nat Genet. 2024 Nov 22;56(12):2704–17. doi: 10.1038/s41588-024-01961-x (PMC11631771; doi:10.1038/s41588-024-01961-x)
Supplement: Supplementary file 1 — Supplementary Note (results and discussion) and Supplementary Figs. 1–59. [file 41588_2024_1961_MOESM1_ESM.pdf]

nature genetics

Supplementary information https://doi.org/10.1038/s41588-024-01961-x

Spatial and single-nucleus transcriptomic analysis of genetic and sporadic forms of Alzheimer's disease

In the format provided by the authors and unedited

Supplementary Results

Data quality control. Due to the heterogeneity inherent to postmortem human brain samples, we examined the distribution of the spatial spots by diagnosis for each cluster and found that most clusters are well represented across the diagnoses, except for WM3 in early-stage AD cases (Supplementary Fig. 1). Two mouse clusters lacked clear spatial localization, one being a mixture of erythrocytes and neurons based on marker gene expression, and the other a group of low quality spots based on UMIs, which we removed from our subsequent analyses (Supplementary Fig. 1). Furthermore, we examined the expression of a post-mortem "ex-vivo activation" signature, previously identified as an artifact of cellular stress during sample processing[1] in both the spatial and snRNA-seq datasets, and while found expression of select ex-vivo activation genes like *HSPA1A*, *HSPA1B*, *FOS*, and *JUN*, the overall expression program score[2] was low (Supplementary Figs. 1, 5).

Differential cell composition analysis. We performed differential cell composition analysis with Milo[3] to test whether the cell states captured by snRNA-seq were enriched or depleted in disease (Supplementary Fig. 6), noting that the largest differences were found among microglia, astrocytes, and vascular cell types. This analysis uses a network-based approach to aggregate transcriptomically similar cells into "cell neighborhoods", and performs a statistical test for the over- or under-representation of different groups (i.e. disease and control) within these neighborhoods[3] (Methods). For example, microglia cluster MG2 (*SPP1*+, *CD74*+, *INPP5D*+) and astrocyte cluster ASC1 (*GFAP*+, *NEAT1*+, *CLU*+) were enriched in disease in our snRNA-seq dataset. Alternatively, astrocyte clusters ASC2 (*RORA*+, *GLI3*+) and ASC4 (*HMGB1*+, *BAG3*+, primarily observed in the PCC), were depleted in disease. Excitatory and inhibitory neuronal cell states were generally well represented among control and disease samples; however based on the effect size distributions we found considerable variability among the cellular neighborhoods within these states stratified by disease status. Among the brain vascular cells, we found the largest differences in cell state abundances within subtypes of pericytes, with extracellular matrix pericytes[4] (M-pericytes) enriched in disease and membrane transport pericytes (T-pericytes) depleted in disease. Imbalances in the composition of the brain vascular compartment like those observed here may be emblematic of the molecular and structural breakdown of the blood-brain-barrier[5]. Together, this analysis revealed shifts in cell state composition throughout neurodegeneration, encompassing both selectively vulnerable and disease reactive states.

Comparative differential expression analyses of sporadic AD and DSAD. We focused our differential expression analyses on the frontal cortex (FCX), since we had both spatial and single-nucleus data for this region, and we found significant positive correlations between effect sizes for gene expression changes in the posterior cingulate cortex (PCC) and FCX across most cell clusters (Supplementary Figs. 10-16). In our spatial data, although the majority of differentially expressed genes (DEGs) were unique to a diagnosis (early-stage AD, late-stage AD, or DSAD), we still discovered many are conserved, including genes previously associated with AD, like *CST3*, *VIM*, *NEAT1*, and *CLU*. As discussed in the main text, we found modest correlations between effect sizes from the different diagnoses in the snRNA-seq data; the cell populations with the weakest correlations across disease conditions included OPC2 and several of the microglial, vascular, and astrocytic clusters.

In DSAD and late-stage AD, we found genes associated with NIK/NF-κB signaling were downregulated in spatial cluster L3/4 and excitatory neuron clusters EX L2/3 and L3-5 (Fig. 2g-h). However, we also found regional differences between DSAD and late-stage AD; NIK/NF-κB signaling genes are upregulated in L1 and L2/3 exclusively in DSAD samples (Fig. 2i-l). Altogether, we identified conserved regional and cell-specific transcriptional changes between late-stage AD and DSAD, supporting the utility of studying DSAD to understand AD molecular changes in both DS and the general population.

Cellular deconvolution of human spatial DEGs. We sought to identify the cellular origins of the spatial DEGs by performing cellular deconvolution analysis of these DEGs (Methods). Inspecting snRNA-seq of the spatial DEGs shared between late-stage AD and DSAD revealed cell-type-specific expression of many spatial DEGs, of which many were also region specific (Fig. 2c). For example the gene *QKI*, previously identified as upregulated in AD[6], is upregulated only in upper cortical layers (L2/3 and L3-5; average log2(fold-change) > 0.2, adjusted p-value < 0.05), although it is highly expressed in WM and oligodendrocytes (Extended Data Fig. 1). In early-stage AD, many oligodendrocyte genes were found among the downregulated DEGs in cortical cluster L6b and the WM clusters, while relatively more of these oligodendrocyte genes were upregulated in cortical clusters for DSAD (Fig. 2d). Intriguingly, early-stage AD downregulated DEGs in L3/4 contained a greater proportion of neuronal genes, although many of the downregulated DEGs did not correspond to any cell marker genes, further supporting that these L3/4 transcriptional changes may be involved in AD-related cognitive decline (Fig. 2d).

Spatiotemporal gene expression changes in 5xFAD. In addition, we wanted to identify evolutionary-conserved AD transcriptional changes, as drug development largely relies on mouse models. However, mouse models of AD have been criticized for discrepancies with clinical AD. Considering previous literature shows baseline regional differences between human and mouse[7,8], we hypothesized that there may be also regional differences in disease. In addition, we believe that focusing on the features shared between human and mouse will help forward *in vivo* research. We performed DE analysis on our mouse ST dataset comparing 5xFAD versus WT within each spatial cluster for each timepoint (Supplementary Figs.

17-20; Supplementary Tables 14-17). This analysis revealed an increased number of upregulated genes with age except in thalamic clusters, where the maximum was at the earliest timepoint (4 months, Supplementary Fig. 21). Upregulated genes at 4 months in the thalamus included disease-associated microglia (DAM) genes[9], like *Cst7*, *Tyrobp*, *Ctsd*, and *Trem2*, suggesting an early response to plaques localized to the thalamus, and with increasing age, we found upregulation of these genes across brain regions. We then examined the expression of DEGs identified in our human ST dataset in a matched comparison with the cortical and WM clusters of our mouse spatial dataset (Supplementary Fig. 22). We found significant and positive fold-change correlations in most cortical layer comparisons, except L1 and L6b. Like our comparison of DSAD and late-stage AD, WM correlations were weaker, indicating that the 5xFAD model recapitulates some but not all clinical AD changes. Our analyses compiled a list of species-conserved, regional DEGs including some genes previously identified in disease-associated glia signatures[9–11].

Multi-scale co-expression network analysis reveals spatial expression programs disrupted in AD . Co-expression networks contain groups of genes (modules) that are highly co-expressed and are potentially co-regulated or involved in shared biological functions. We constructed spatial co-expression networks and identified gene modules individually for each cortical layer cluster and WM, yielding 166 modules from seven networks (network plot in Fig. 3a). Based on an approach previously employed in a brain-wide network analysis in bulk RNA-seq[12], we defined a set of 15 cortex-wide "meta-modules" by hierarchically clustering these 166 modules based on their similarity in gene expression patterns (module eigengenes, MEs) and their constituent gene sets (dendrogram and triangular heatmaps in Fig. 3a, Methods). We used the ST dataset and the snRNA-seq dataset to evaluate the spatial and cellular expression patterns of the meta-modules, noting that many of these expression signatures correspond to grey matter regions and neurons, and that some were expressed across multiple regions and cell types (Figs. 3d).

We compared effect sizes between the differential module eigengene (DME) tests for each of the 166 modules to assess the similarity in network-level changes between subtypes of AD (Figs. 3b). The DMEs that were shared across these disease groups contained hub genes associated with key AD-related processes like amyloid-beta formation, myelination and chromatin organization (Extended Data Fig. 3). We found that the largest set of unique DMEs were those downregulated in early-stage AD, highlighting the role of transient systems-level expression changes in the molecular cascade underlying AD progression (Extended Data Fig. 3). The early disease alterations in L3/4 also support our DEG findings and pinpoint gene networks that may contribute to the initial cognitive symptoms of AD, such as memory deficits and impaired information processing. Further, while the majority of the DMEs specific to early-stage AD were downregulated, the majority of DMEs specific to late-stage AD and DSAD were upregulated compared to controls. Although these modules were transcriptomically distinct across the different network analyses, the hub genes for these modules upregulated in late-stage AD and DSAD were enriched for genes associated with neurotransmission, neuronal development, and RNA processing.

Hemoglobin and ATP synthase subunit expression in DSAD females. Examining the sex DEGs identified in DSAD from our ST dataset, gene set overlap analysis demonstrated that many of these DEGs with the largest effect sizes were shared across multiple spatial clusters, such as the hemoglobin subunit beta gene *HBB* and the complement subcomponent gene *C1QB* in females, and the myelin associated gene *MOBP* in males (Fig. 4g). Although *HBB* expression in female samples may be partly due to sample variability in blood contamination, hemoglobin is also expressed by neurons and glia. A previous study found protein-protein interactions between Hbb and ATP synthase[13], and we also identified ATP synthase subunits (*ATP5PD* and *ATP5MPL*) as female DEGs.

Additional sex-specific signatures in AD. We sought to establish whether the sex-related DEGs were similar among disease groups, similar to the disease vs. control DEGs, or similar between human and mouse. First, we compared DE effect sizes between disease groups in the human ST dataset (Supplementary Fig. 39). In each region, we found genes whose DE effect size directions were in agreement, as well as those which were discordant across the disease groups. When comparing DSAD to the other disease groups, we generally observed more differences than similarities; however, this varied between regions. For example, WM DE effect sizes between DSAD and early-stage AD were positively correlated (R=0.21), while there was a negative correlation between late-stage AD and early-stage AD (R=-0.47). Sex DEGs in DSAD and late-stage AD showed greater agreement in the snRNA-seq dataset than in the ST dataset (Supplementary Fig. 40). We next compared DE effect sizes between the sex DEGs and the diagnosis DEGs in the ST and snRNA-seq datasets (Supplementary Figs. 41-44), and we found that some of the genes that were not DE between disease and controls showed sex-level differences in the disease groups. Finally, we compared the sex DEGs between the human and mouse datasets (Supplementary Fig. 45), and we found a greater agreement between the 5xFAD mice and DSAD compared to early and late-stage AD, which may be related to relatively higher amounts of amyloid deposition in the 5xFAD and DSAD groups.

Imaging mass cytometry to examine differences in AD protein and RNA expression changes. Imaging mass cytometry (IMC) uses a panel of metal-conjugated antibodies for multiplexed labeling, followed by fast UV-laser tissue ablation

and cytometry by time of flight (CyTOF) to yield quantitative single-cell spatial proteomic data at approximately 1μm resolution, and this approach has been previously applied in postmortem human brain tissue[14]. The constructed images remarkably mirror images obtained from classical microscopy but with greater channel capacity (Figs. 5b-c). This method circumvents the constraints of conventional immunohistochemistry, which only allows for analysis of a handful of markers, restricting the understanding of cellular phenotypes, and avoids the autofluorescence issues inherent to aged human brain tissue. We applied a supervised machine learning approach to generate cell segmentation masks, allowing us to quantify protein intensities for individual cells, yielding 32,759 cellular profiles after quality controls (Methods). Our antibody panel did not include marker proteins for all brain cell types due to limited antibody availability and compatibility. However, we were able to profile neurons, astrocytes, and microglia.

Using this dataset, we were able to investigate the congruence between protein and gene expression changes in AD. For example, we found Cystatin C (*CST3*) significantly changed in microglia and astrocytes from late-stage AD and DSAD (Fig. 5i). *CST3* also was upregulated in both groups in spatial clusters L3-5 and L5/6 but was upregulated in only ASC1 for DSAD and MG2 for late-stage AD in our snRNA-seq data. Similar to *CST3*, we found that *CD44* expression in the ST data was more concordant with IMC data (Figs. 5j-k) than that in the snRNA-seq data. We expected CD44 upregulation in astrocytes, but we did not detect microglial upregulation of *CD44* at the single-nucleus transcriptome. These discrepancies may be due to the biological discrepancy between gene and protein expression or other technological and algorithmic limitations. In sum, our IMC experiment in light of our other -omics experiments highlights the importance of a multi-faceted approach to comprehensively understand the altered molecular signatures of the brain in neurodegeneration.

Spatial annotation of cell clusters to identify cell-cell signaling dysregulated in disease. While excitatory neuron subpopulations clustered and were easily annotated by cortical layer, we were not able to stratify inhibitory neurons and non-neuronal cells by region with snRNA-seq alone. Therefore, we sought to leverage our ST data to infer region localization of our snRNA-seq clusters. To go beyond cell-type deconvolution analysis (Supplementary Figs. 46-47), we used CellTrek[15] to integrate spatial and single-cell transcriptomic data and predict spatial coordinates of each cell. For this analysis, we computed paired co-embeddings for each of the 39 human ST samples with the 48 snRNA-seq cortical samples generated in this study and used CellTrek to train a multivariate random forest model to predict spatial coordinates for each cell using the shared embeddings (Methods, Extended Data Fig. 6a). We visualized these results in representative spatial samples by plotting the mapped snRNA-seq cells colored by their cell identity split by major cell lineages (Extended Data Fig. 6a), as well as the expression of selected cortical layer marker genes side by side with the ST and mapped snRNA-seq datasets, confirming this approach assigns cell types to their known brain region (Extended Data Fig. 6b). Since this analysis was performed on each of the ST samples, each cell from the snRNA-seq data was mapped to multiple spatial contexts. Using this information, we computed a probability for each mapped cell to each of the spatial clusters. For example, this analysis uncovered that a subpopulation of *LAMP5*+ inhibitory neurons are WM interstitial neurons[16] (Extended Data Fig. 6c-d). Finally, for the snRNA-seq data we added a label for upper cortical layer, lower cortical layer, or WM based on the spatial context that the cell was most frequently mapped to (Methods). We performed differential cell composition analysis using these updated annotations (excluding the spatially mapped cell states with 10 or fewer Milo cell neighborhoods) and identified new trends that were masked in our previous analysis without spatial information (Extended Data Fig. 6e). For example, our previous analysis found that ASC1 was enriched in DSAD (Supplementary Fig. 6), and this analysis shows that this enrichment is greater in the WM than the cortex. Additionally, we found that WM pericytes were enriched in disease, while cortical pericytes were depleted (Extended Data Fig. 6e). This analysis also revealed a considerable shift in the composition of all inhibitory neuron subtypes in disease, where deep layer inhibitory neurons were relatively less abundant compared to upper layer inhibitory neurons. Furthermore, we inspected the predicted cell density distributions of specific cell subpopulations like subtypes of astrocytes and microglia in representative ST samples (Extended Data Fig. 6f). We also used these mappings to directly investigate the spatial and cellular contexts of systems-level transcriptomic signatures by visualizing the meta-module eigengenes in the predicted ST coordinates split by the major cell types (Extended Data Fig. 6g).

We used CellChat[17] to infer cell signaling between different cell states based on the expression of ligand and receptor pairs and perform statistical testing to compare the signaling patterns between control and disease (Fig. 6a). The additional spatial information allows us to distinguish these signaling changes as short- or long-range cellular communication, which is otherwise lost with snRNA-seq data alone. We compared the CellChat results to LIANA[18], another CCC inference method that computes consensus results from a variety of individual inference methods and ligand-receptor databases (Methods), and we found that the number of predicted signaling links between cell states were correlated across the two methods (Supplementary Figs. 48-49). For this analysis, we chose to only include the snRNA-seq data generated in this study since current CCC inference tools do not explicitly account for batch effects.

Downregulation of CD99 signaling in AD. *CD99* is also a hub gene of M11, but CD99 signaling is downregulated in DSAD (Extended Data Fig. 8). We discovered pericyte and astrocyte (ASC3) communication with excitatory and inhibitory neuronal populations is absent, suggesting that disrupted CD99-CD99L2 signaling may contribute to neurovascular changes seen in AD, and we confirmed by immunofluorescence that CD99-CD99L2 colocalization is significantly decreased in both

late-stage AD and DSAD compared to control (Extended Data Fig. 8f-g). Notably, despite the overall downregulation of CD99 signaling in DSAD, we also found CD99-PILRA signaling between cortical lower layer pericytes and microglia exclusively in DSAD. PILRA, a myeloid inhibitory signaling receptor, has been associated with AD genetic risk[19–22]; our findings implicate pericytes in the modulation of microglial function in AD via PILRA activity.

Integrating spatial transcriptomics with fluorescent amyloid imaging to identify pathology-associated gene expression signatures. Amyloid pathology is a defining feature of AD, and there is a critical need to understand the development of amyloid pathology and the cellular response. Previous studies identified amyloid-associated cell subpopulations that are transcriptionally distinct[9–11] as well as plaque-associated gene expression changes[23]. However, these studies did not investigate amyloid-associated gene expression changes regarding the conformational changes associated with plaque formation, and they did not use a uniform approach in humans and mice.

We devised a strategy to identify amyloid-associated gene expression changes in both our human and mouse ST datasets (Methods). Immunofluorescent staining ST tissue sections with Amylo-glo and the antibody OC yielded information about the size and spatial location of amyloid aggregates in the same coordinates as our gene expression data, and we developed a custom image analysis pipeline to integrate these amyloid quantifications with our ST datasets. We performed geospatial statistical analysis[24–26] to identify "hot spots" of plaques and fibrils (Figs. 7d-e, Supplementary Figs. 52-55), and used generalized linear models (GLMs) to perform differential gene expression testing with respect to the amyloid hot spot intensity (Methods). Critically, this analysis was agnostic of disease status or mouse age groups to specifically identify genes associated with amyloid rather than those associated with other disease-related factors.

Since amyloid depositions appear in grey matter much more frequently than in WM, for the human dataset we focused our analysis on the gray matter regions (Methods). Gene set overlap analysis showed that 55 genes were common between the plaque- and fibril-associated genes (Fig. 7f, Fisher's exact test p-value = 1.8e-115, odds ratio = 1248.92) in the human dataset. There were more amyloid-associated genes identified in the mouse dataset compared to the human dataset, likely because the mouse dataset profiled several different brain regions each with their own unique transcriptomic profiles while the human dataset only profiled the PFC. We found that some of the mouse amyloid-associated gene sets had significant overlaps between those found across different spatial clusters, but most of these genes were spatially restricted to only one of these groups. Perhaps surprisingly, we noted a general lack of microglial genes in the human amyloid-associated genes, whereas several of these mouse amyloid gene sets, including those from cortical regions, contained microglial genes like *Trem2* and *Tyrobp*. However, we identified *MEF2C*, a microglial transcription factor associated with AD genetic risk[19,20], in the shared amyloid genes.

We compared the amyloid-associated gene sets identified in this study with plaque-induced gene (PIG) module from another amyloid mouse model[23], in addition to other relevant gene signatures like disease-associated microglia (DAMs[9]), astrocytes (DAAs[10]), and oligodendrocytes (DOLs[11]), which were originally identified in 5xFAD mice. We performed gene set overlap analysis, and we computed gene signature scores for these gene sets in the mouse and human ST dataset (Methods), revealing significant overlaps between our mouse amyloid-associated genes with these published gene sets (Fig. 7k). Since the PIG module was previously identified using co-expression network analysis, we performed a similar network analysis in our 5xFAD ST dataset to see if we could recover a similar expression program using hdWGCNA[27] (Methods, Extended Data Figs. 9-10, Supplementary Fig. 57, Supplementary Table 38). One of the gene modules identified in this analysis (SM6) was enriched for genes related to microglial activation and amyloid-beta response, and the expression of this module increased with age in 5xFAD but not WT mice (Extended Data Fig. 9). Further, we found that this module significantly overlapped with the PIG module, and that the expression of the PIG module also only increased with age in 5xFAD mice (Supplementary Figs. 58, 59). Altogether, these findings demonstrate that these amyloid-associated gene expression changes are highly reproducible and are mouse model-agnostic.

Supplementary Discussion

Single-cell sequencing has uncovered cell state-specific changes in disease, revealing novel insights into disease pathophysiology and gene targets for further study. A critical limitation of these methods is their lack of spatial information. AD pathology progresses in a predictably anatomical manner that may be linked to brain circuitry. Recently, several spatial profiling methods were developed with varying levels of resolution, throughput, and number of genes[28–33]. Spatial transcriptomics (ST) relies on a grid of spots with primers to uniquely barcode transcripts based on their spatial location and does not require pre-determined gene targets, allowing an unbiased assessment of gene expression. However, the size of each spot is 55µm, resulting in transcriptomic profiles that may be from 1-10 cells. We generated ST data from postmortem human brain tissue samples of clinical AD, encompassing early- and late-stage AD in the general population, and DSAD. Due to technical limitations, we could not profile gene expression at a macroscopic level of multiple brain regions, and our samples had varying amounts of cortical layers and WM. We also generated ST data from the 5xFAD mouse model at four ages, allowing us to simultaneously assess multiple brain regions and the temporal dynamics of amyloid accumulation, with reduced sample heterogeneity compared to

the human dataset. These human and mouse ST datasets are the largest of their kind to our knowledge (n=119 total), together encompassing a rich data resource for the AD community.

Further, we generated a unique snRNA-seq dataset of DSAD and integrated previously published AD studies to identify cellular changes conserved between both populations. Differential cell abundance analysis showed enrichment of activated microglial and astrocyte cell states in DSAD, as well as altered makeup of the vascular compartment, compared to cognitively normal controls. Many of the genes upregulated in all disease groups were attributed to glial and vascular cells based on our DEG deconvolution analysis, where notably more oligodendrocyte changes were found in the WM in early and late-stage AD. In addition, inspecting spatial gene co-expression modules in our snRNA-seq data provided cellular contexts of these gene programs, uncovering neuronal and glial components that may be integral to disease biology.

Recent efforts in genome-wide association studies (GWAS) and fine mapping analyses of sporadic AD have nominated numerous candidate risk loci and risk genes[19,34–37]. Disease risk SNPs, cis-regulatory elements, genes, and proteins do not operate in a vacuum, and a disease-associated gene may play a role in a broader regulatory network that ultimately contributes to a disease phenotype[38]. Several previous studies have found associations between microglial cis-regulatory elements and AD genetic risk in mouse[39] and human brains[40–42]. Additionally, key shifts in the transcriptional and epigenomic identities of glia have been previously observed in single-cell studies of human AD[42] and mouse models[9,10,43], where AD risk genes and loci are directly involved in these shifts in microglia. Integration of polygenic disease enrichment with multi-scale gene co-expression networks has the potential to widen the scope of our understanding of AD genetics. We performed a series of genetic enrichment analyses with scDRS[44] and found evidence of association between AD risk and meta-module M11 based on the human and mouse ST datasets and the human snRNA-seq dataset. Interestingly, the ligands *CD99* and *ANGPTL4*, identified in our cell signaling analyses, are hub genes in M11, and a few genes specific to fibrillar amyloid are also members of M11, including *AEBP1* and *DTNA*, both previously found associated with AD[45–47].

While previous 5xFAD studies identified amyloid-associated subpopulations and genes, not all are recapitulated in clinical AD, and this has been previously highlighted in human AD snRNA-seq studies examining the DAM signature. One study that focused on TREM2 genetics in human AD and in mouse models found remarkable transcriptomic changes in reactive microglia[48], while another identified a network of plaque-induced genes in an APP mouse model, but it remains unclear to what degree analogous signatures are observed in humans[23]. While this may be due to single-cell vs single-nucleus comparisons[49], a previous snRNA-seq study using human AD and 5xFAD samples[48] in addition to our previous re-analysis[42] proposed that this may be a species difference. Identifying cross-species transcriptomic changes are of particular interest for mouse model development and preclinical translation. We evaluated the expression of DEGs identified in clinical AD in the 5xFAD dataset, identifying regional evolutionary-conserved disease gene expression changes. The large genetic component of AD, which underlies the axis of cognitive resilience-susceptibility, greatly contrasts the uniform genetic background of AD mouse models like 5xFAD[50]. While mouse models may provide limited insights into human AD, comparative cross-species -omics analyses would likely be improved using a genetically diverse mouse strain[51]. Furthermore, it is important that future studies include larger and more complete human datasets to properly disentangle biological differences between species from technical differences.

Finally, we generated a spatial proteomic dataset to examine the protein expression of genes we identified in our transcriptomic datasets, in addition to other AD-related proteins. In this dataset, we discovered increased tau and amyloid pathology in DSAD cells compared to late-stage AD. A limitation of our transcriptomic analyses is our focus on amyloid pathology. Although we did not observe dramatic transcriptional differences between DSAD and late-stage AD, tau pathology better correlates with cognitive decline; future studies investigating the individual and combined effects of tau and amyloid pathology in DSAD may help to disentangle the molecular differences between these groups. Notably though we found that findings in the ST data were upheld at the protein level; however, we could not confirm layer-specific expression due to technical limitations. snRNA-seq results were less concordant with the proteomic data. Although this may be due to profiling only nuclear RNA, resultant protein expression may also be affected by posttranscriptional and posttranslational regulatory changes, as well as protein trafficking, which may not be captured in transcriptomic data. High-throughput methods to assess protein expression are also still limited, and the availability of effective antibodies remains a large impediment in the study of proteins. Despite these limitations, the proteomic data supported that M11 represents key AD co-regulated genes, as we found the encoded proteins of both hub genes *CD44* and *CLU* upregulated in AD glia, demonstrating our integrative analytical approach uncovers functional information that enriches our understanding of disease-relevant gene targets.

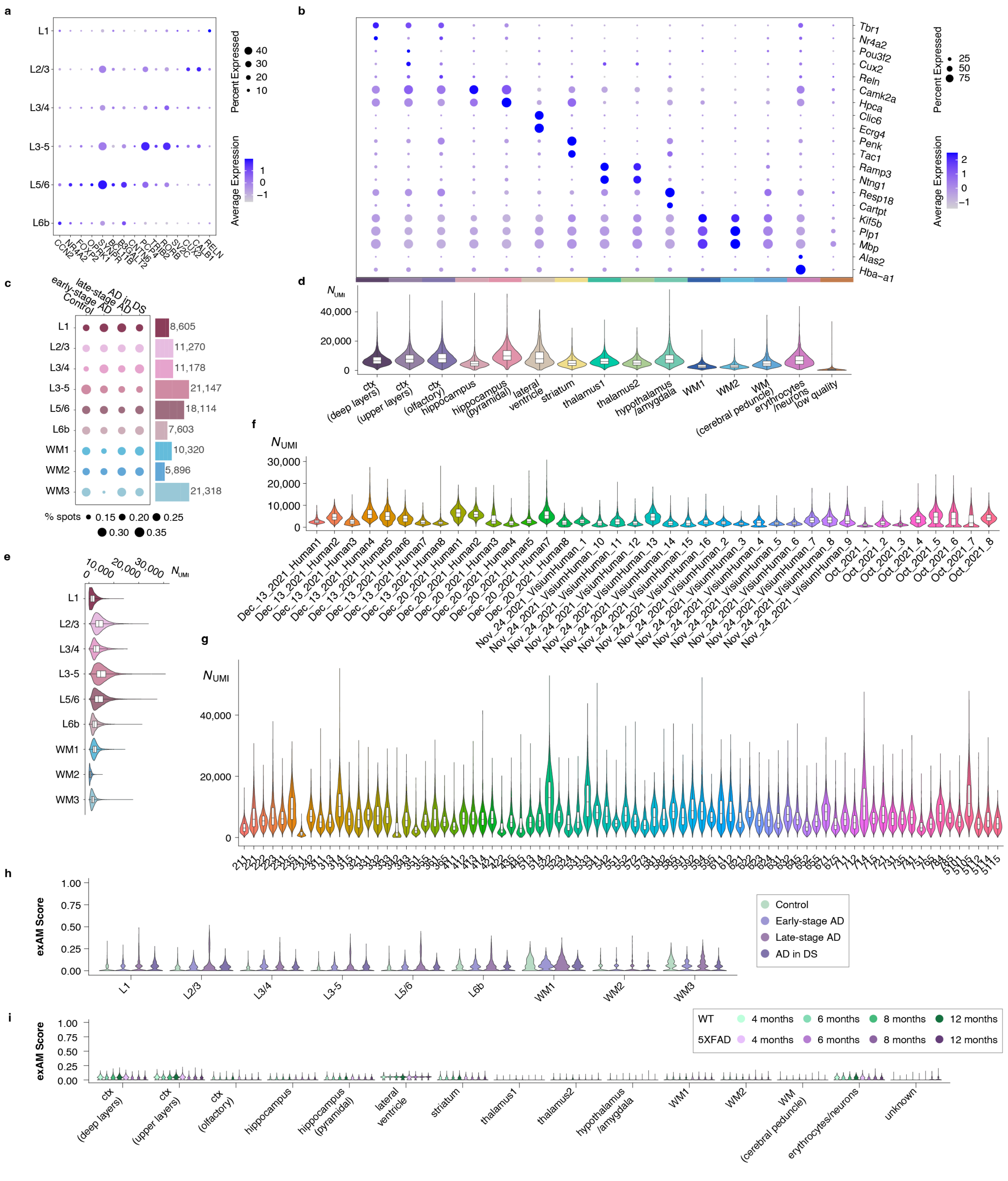

Supplementary Fig. 1 | Quality control of the human and mouse spatial transcriptomics datasets. a, Dot plot showing the expression of selected marker genes in the cortical layer clusters from the human ST dataset. **b**, Dot plot showing the expression of selected marker genes in the clusters from the mouse ST dataset. **c**, The number of spots profiled in each cluster, and the proportion of diagnosis attributed to each of the clusters. **d-e**, Distributions of the number of UMI captured in each of the mouse (**d**) and human (**e**) ST clusters. Each data point represents a ST spot, and the number of ST spots per cluster is noted in Supplementary Table 1. **f-g**, Distributions of the number of UMI captured in each of the human (**f**) and mouse (**g**) samples. Number of ST spots per cluster noted in Supplementary Tables 1-2. **h-i**, Distributions of ex-vivo activation [1] UCell [2] scores in each of the ST clusters in the human (**h**) and mouse (**i** clusters). For box and whisker plots, box boundaries and line correspond to the interquartile range (IQR) and median, respectively. Whiskers extend to the lowest or highest data points that are no further than 1.5 times the IQR from the box boundaries.

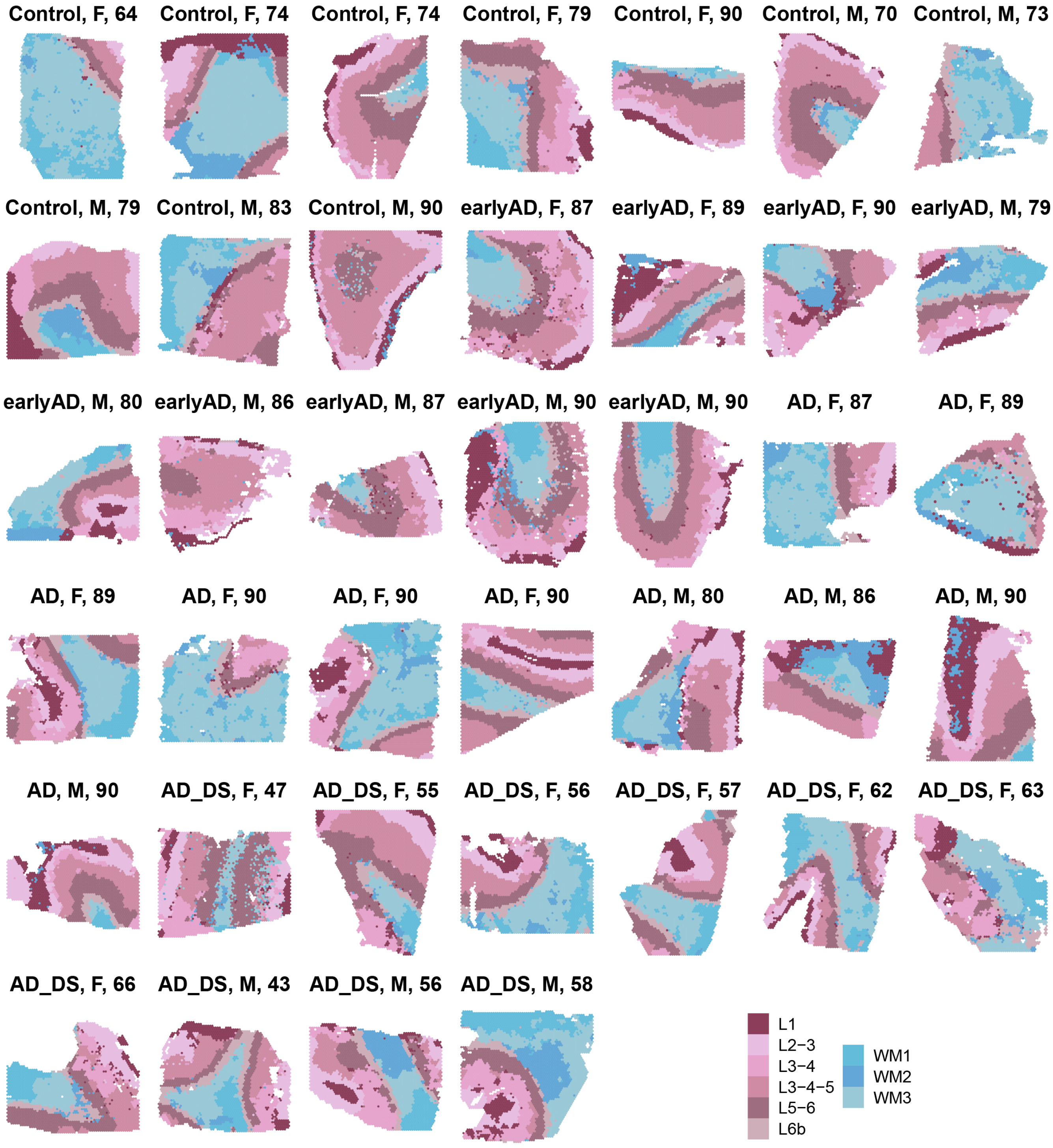

Supplementary Fig. 2 | Clustering results in the human ST dataset. We used BayesSpace[52] to jointly cluster spatial transcriptomic spots in the 39 samples from the human dataset based on their transcriptomic content as well as their spatial information (Methods). This process resulted in nine clusters, which we annotated based on marker gene expression and anatomic location. Each human ST sample is shown here with each spot colored by BayesSpace cluster assignment. We organized the samples in this plot by disease status, sex, and age.

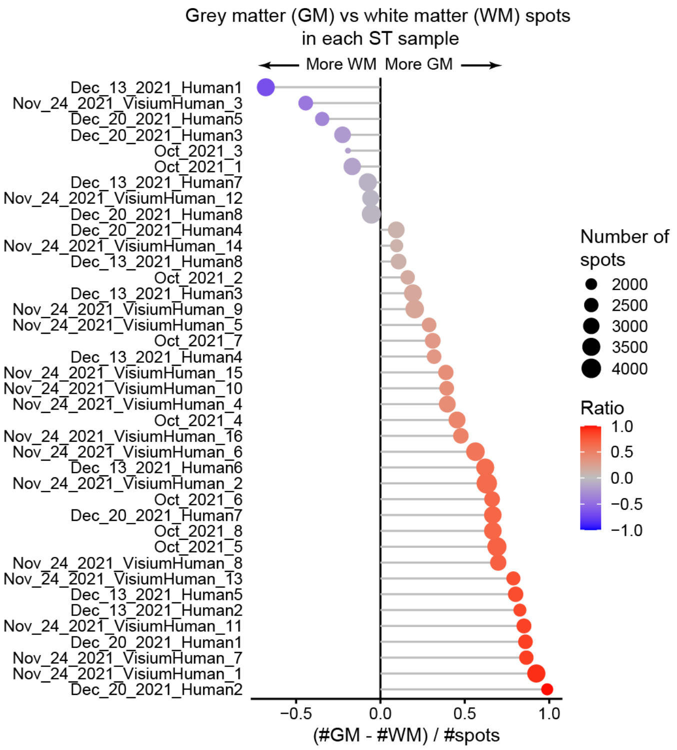

Supplementary Fig. 3 | Comparison of grey and white matter content in human ST samples. For each of the human ST samples, we calculated the difference between the number of grey matter spots and white matter spots, then divided by the total number of spots. Grey matter vs white matter was determined based on our unbiased clustering analysis. We then ranked each sample based on this metric. In this plot we also visualize the total number of spots per sample as the size of each dot.

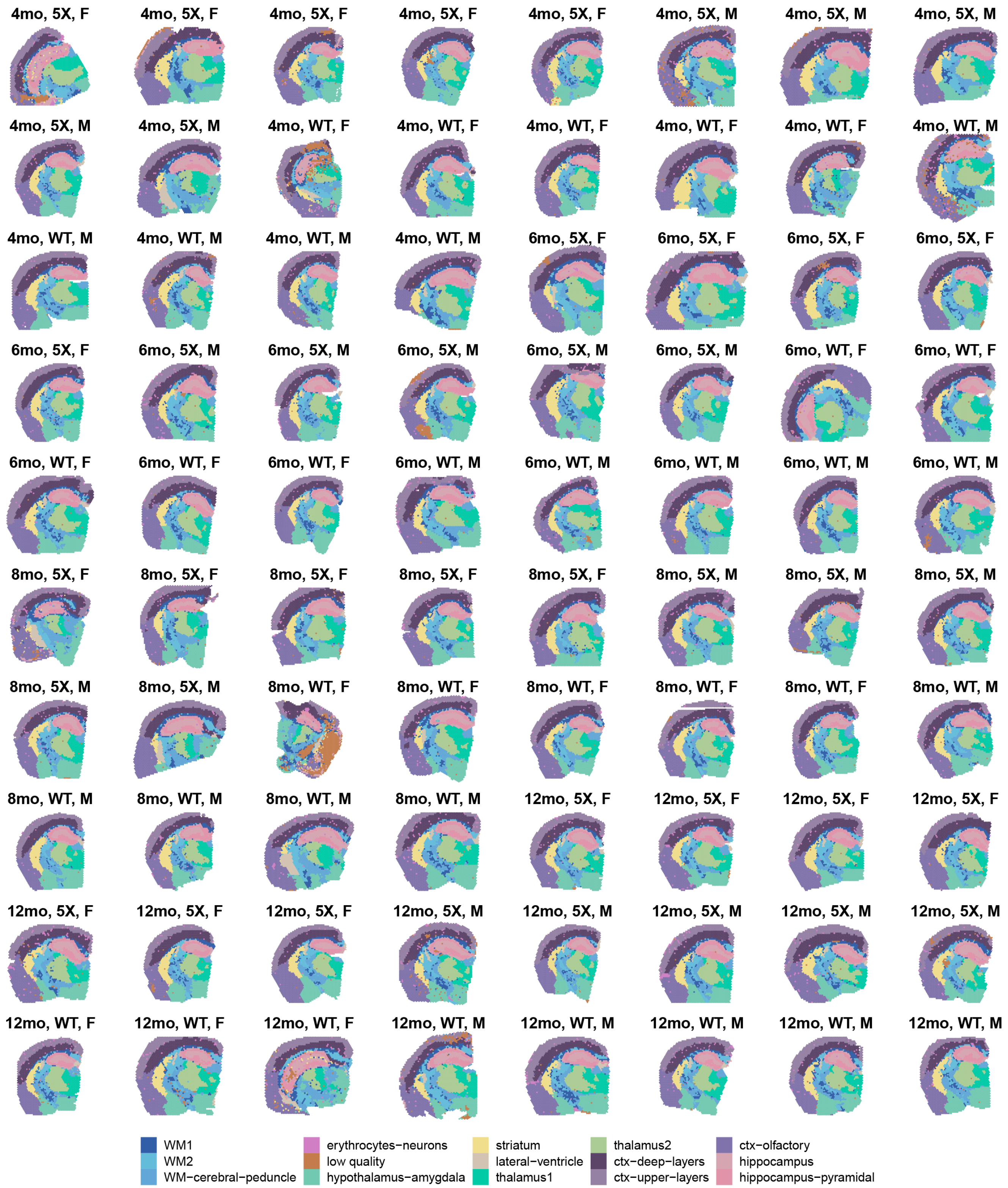

Supplementary Fig. 4 | Clustering results in the mouse ST dataset. We used BayesSpace [52] to jointly cluster spatial transcriptomic spots in the 80 samples from the mouse dataset based on their transcriptomic content as well as their spatial information (Methods). This process resulted in 15 clusters, which we annotated based on marker gene expression and anatomic location. Each mouse ST sample is shown here with each spot colored by BayesSpace cluster assignment. We organized the samples in this plot by disease age, genotype, and sex.

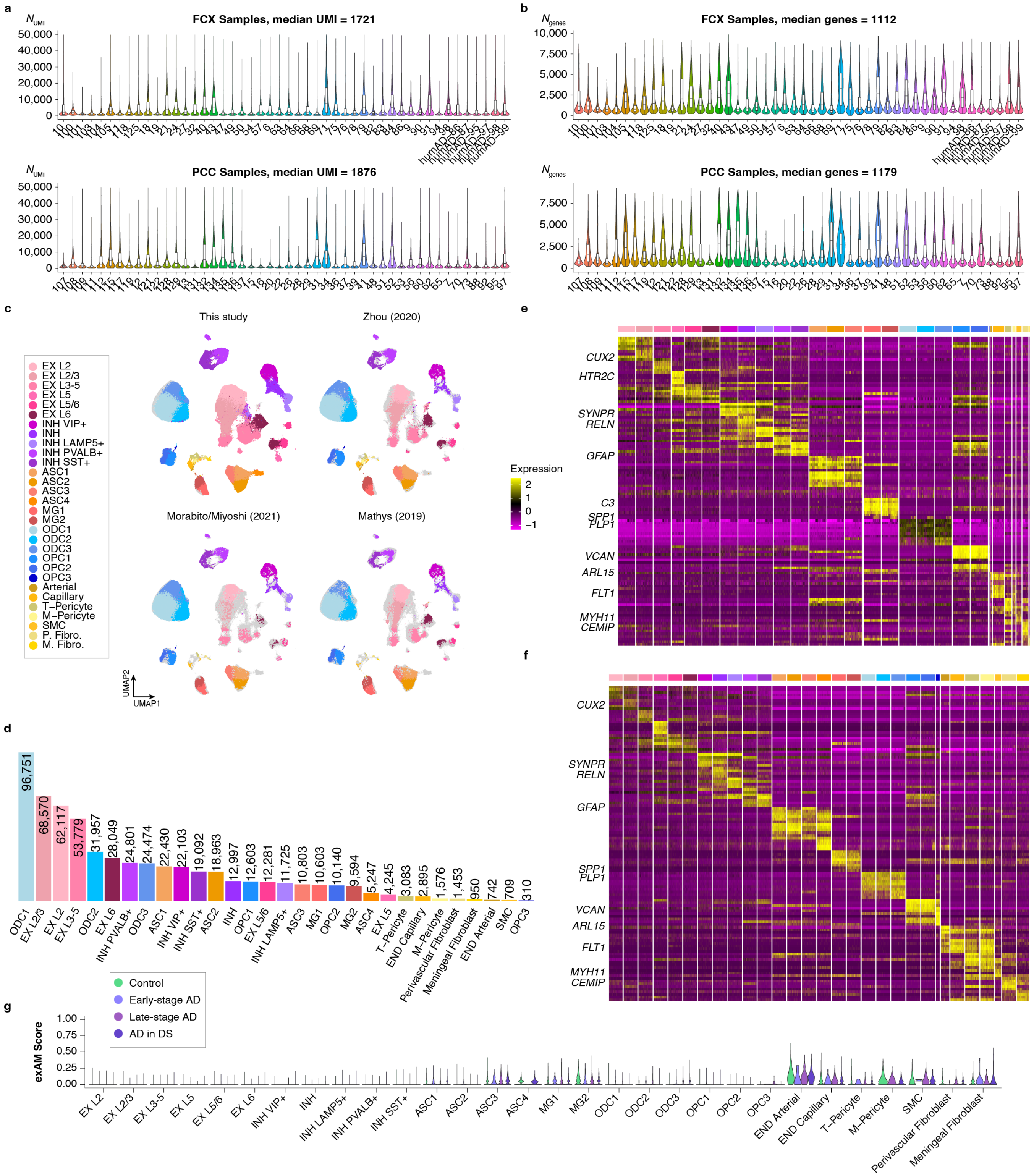

Supplementary Fig. 5 | **Quality control of the integrated snRNA-seq dataset.** **a**, Distributions of the number of UMI captured in each snRNA-seq sample for the frontal cortex (FCX, top) and the posterior cingulate cortex (PCC, bottom). **b**, Distributions of the number of genes captured in each snRNA-seq sample for the FCX (top) and the PCC (bottom). **c**, UMAP dimensionality reduction plot of the integrated snRNA-seq dataset comprised of the data generated in this study and three previous snRNA-seq datasets of AD. The UMAP plot is faceted by study of origin, and colored by cluster annotations. **d**, Bar plot showing the number of nuclei in each of the snRNA-seq clusters. Each data point represents a cell barcode from the snRNA-seq dataset from Milo, and the number of barcodes shown per sample are listed in Supplementary Table 5. **e**, Heatmap showing the expression of the top 5 marker genes ranked by effect size in the snRNA-seq dataset. These marker genes were identified using only the data generated in this study, and this heatmap only shows expression data from these nuclei (Methods). **f**, Heatmap showing the expression of the same genes as in panel (**e**) in the three other snRNA-seq datasets. **g**, Distributions of ex-vivo activation[1] UCell[2] scores in each of the snRNA-seq clusters. For box and whisker plots, box boundaries and line correspond to the interquartile range (IQR) and median, respectively. Whiskers extend to the lowest or highest data points that are no further than 1.5 times the IQR from the box boundaries.

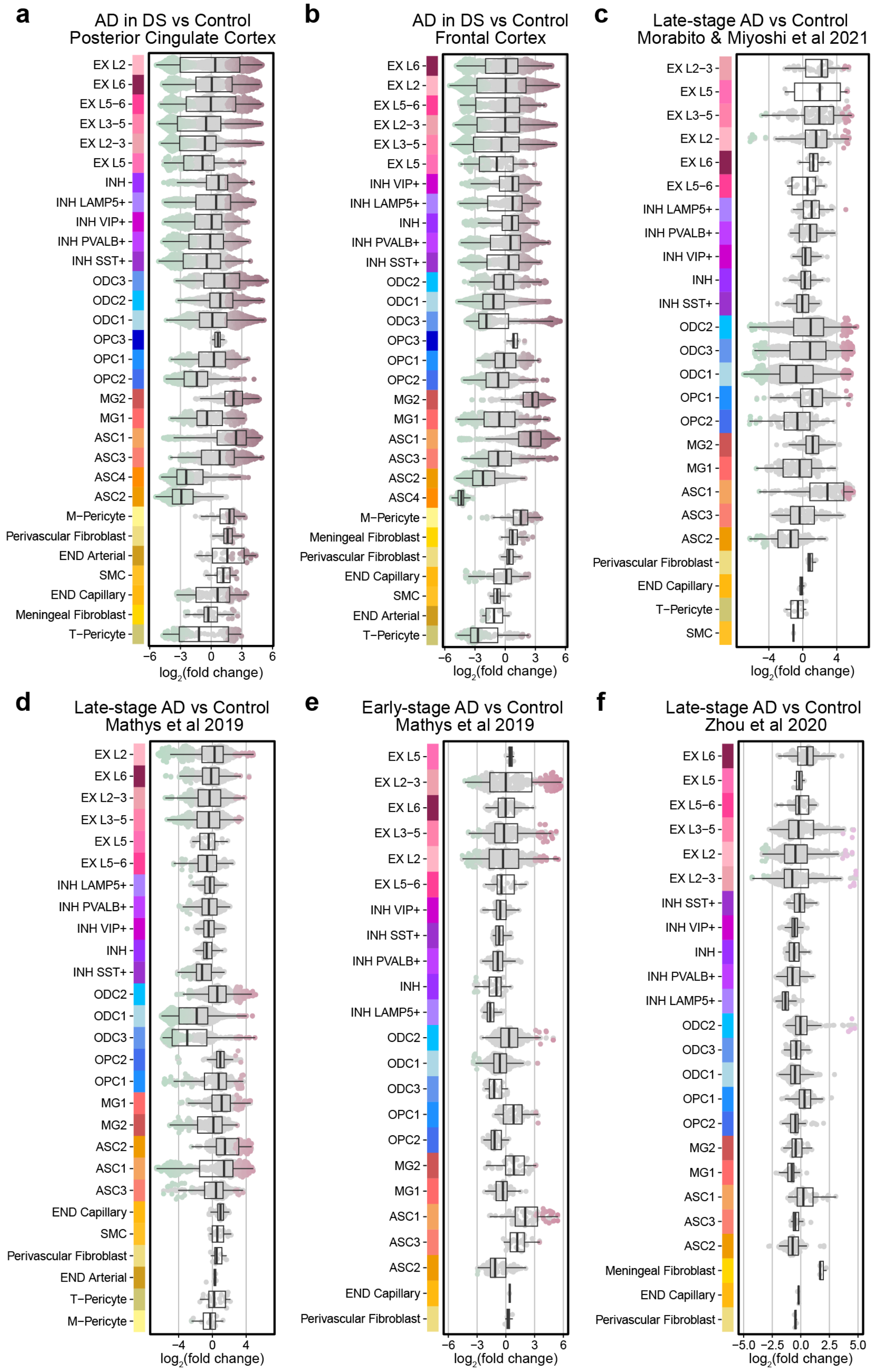

Supplementary Fig. 6 | Differential cell state composition in snRNA-seq datasets. a-f, Box and whisker plots showing the results of the differential cell state composition tests using MiloR[3]. Each data point represents a cellular neighborhood from the Milo algorithm, and the differential cell composition between disease (right) and control (left) effect sizes are shown. Groups are organized on the y-axis by major cell types, and ordered by median fold-change values within each cell type. For box and whisker plots, box boundaries and line correspond to the interquartile range (IQR) and median, respectively. Whiskers extend to the lowest or highest data points that are no further than 1.5 times the IQR from the box boundaries. Each data point represents a single cell neighborhood from Milo, and the number of cell neighborhoods per cluster are shown in Supplementary Table 5.

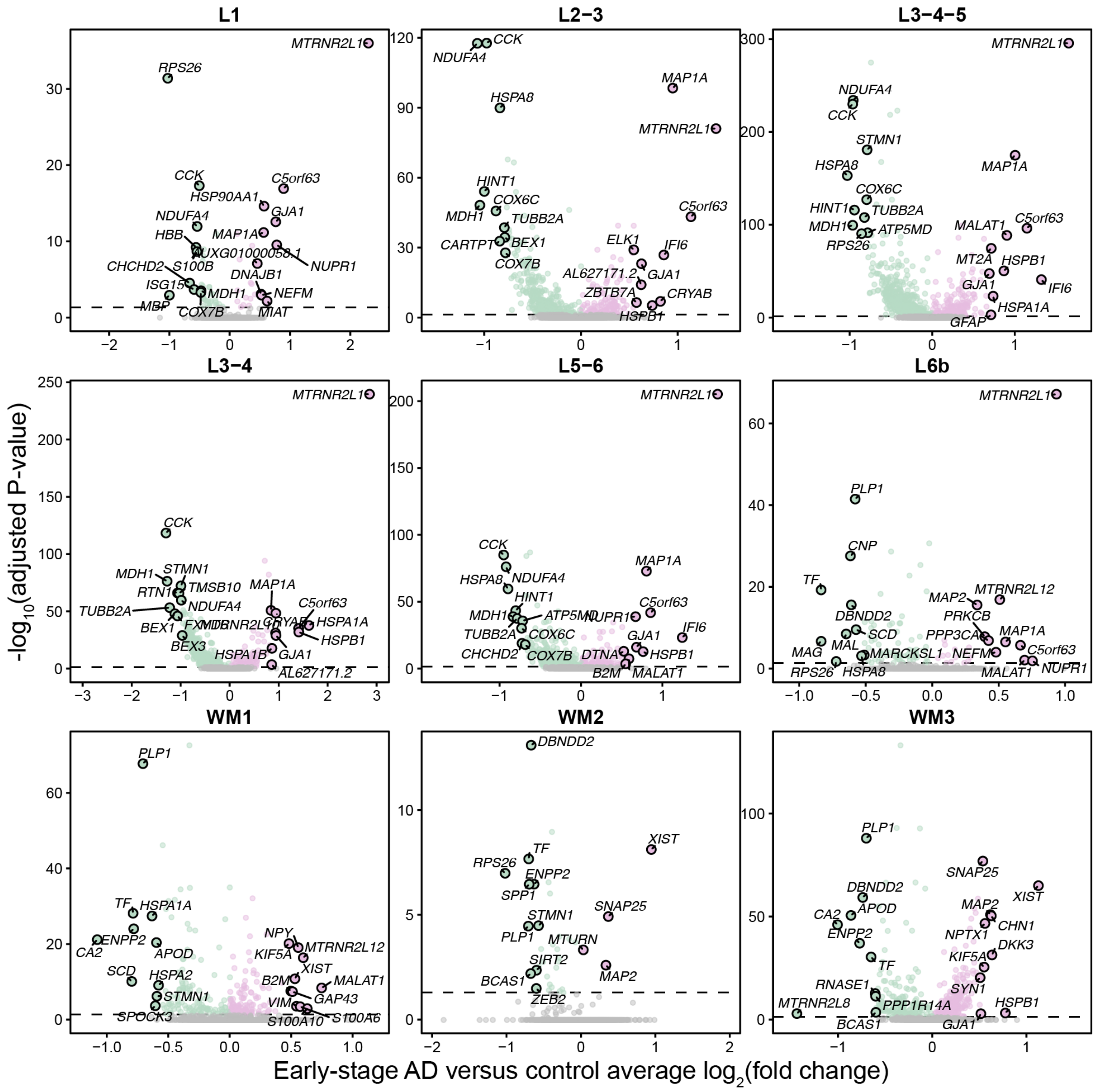

Supplementary Fig. 7 | Early-stage AD versus control DEGs in the ST dataset. Volcano plots show the adjusted significance levels and effect sizes from the differential gene expression comparisons between early-stage AD cases versus cognitively normal controls in the human ST dataset. The results are shown for the differential gene expression analysis in each of the nine spatial clusters. The top and bottom ten genes by effect size are annotated. Statistical test: MAST[53], two-sided test.

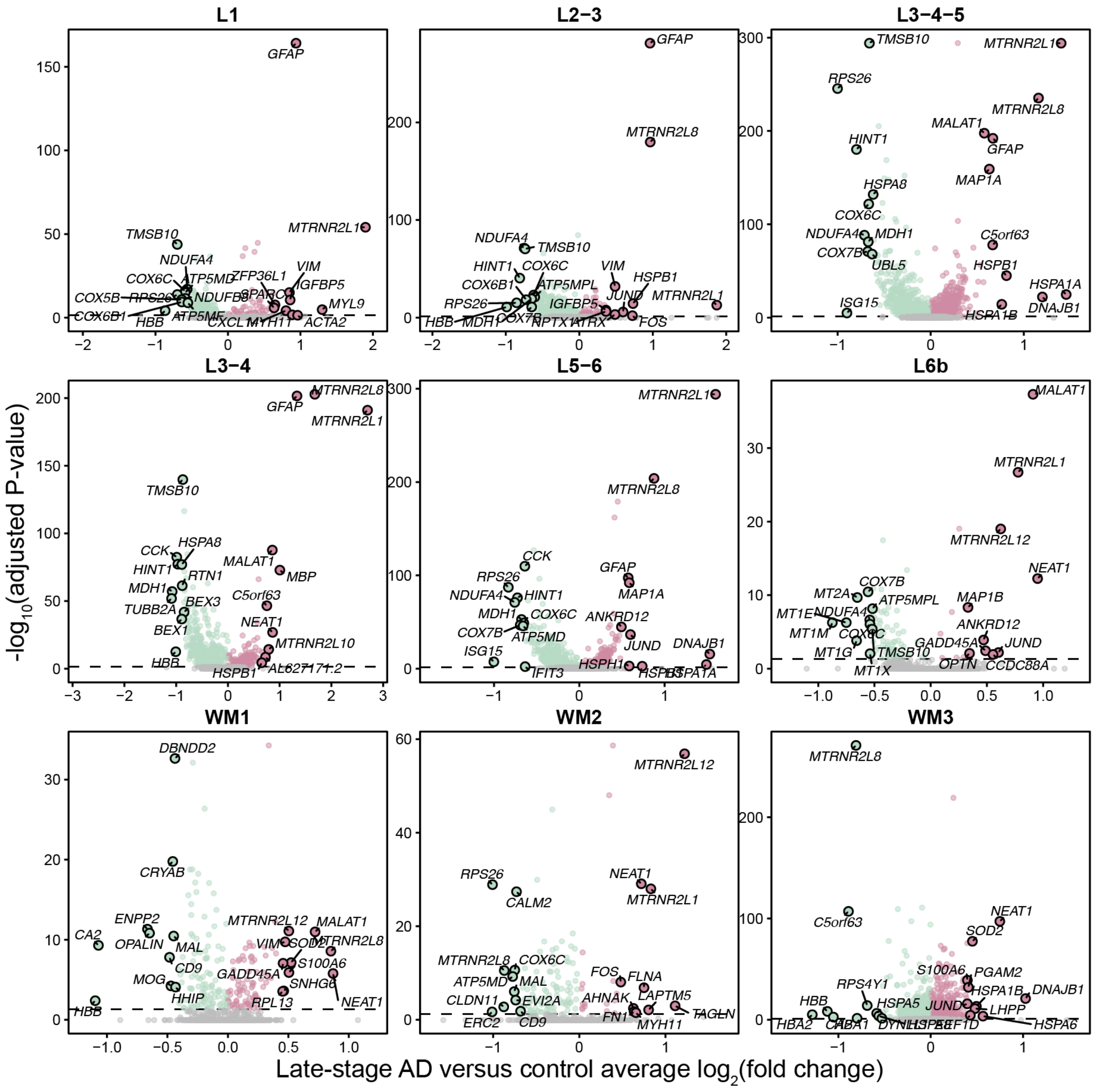

Supplementary Fig. 8 | Late-stage AD versus control DEGs in the ST dataset. Volcano plots show the adjusted significance levels and effect sizes from the differential gene expression comparisons between late-stage AD cases versus cognitively normal controls in the human ST dataset. The results are shown for the differential gene expression analysis in each of the nine spatial clusters. The top and bottom ten genes by effect size are annotated. Statistical test: MAST[53], two-sided test.

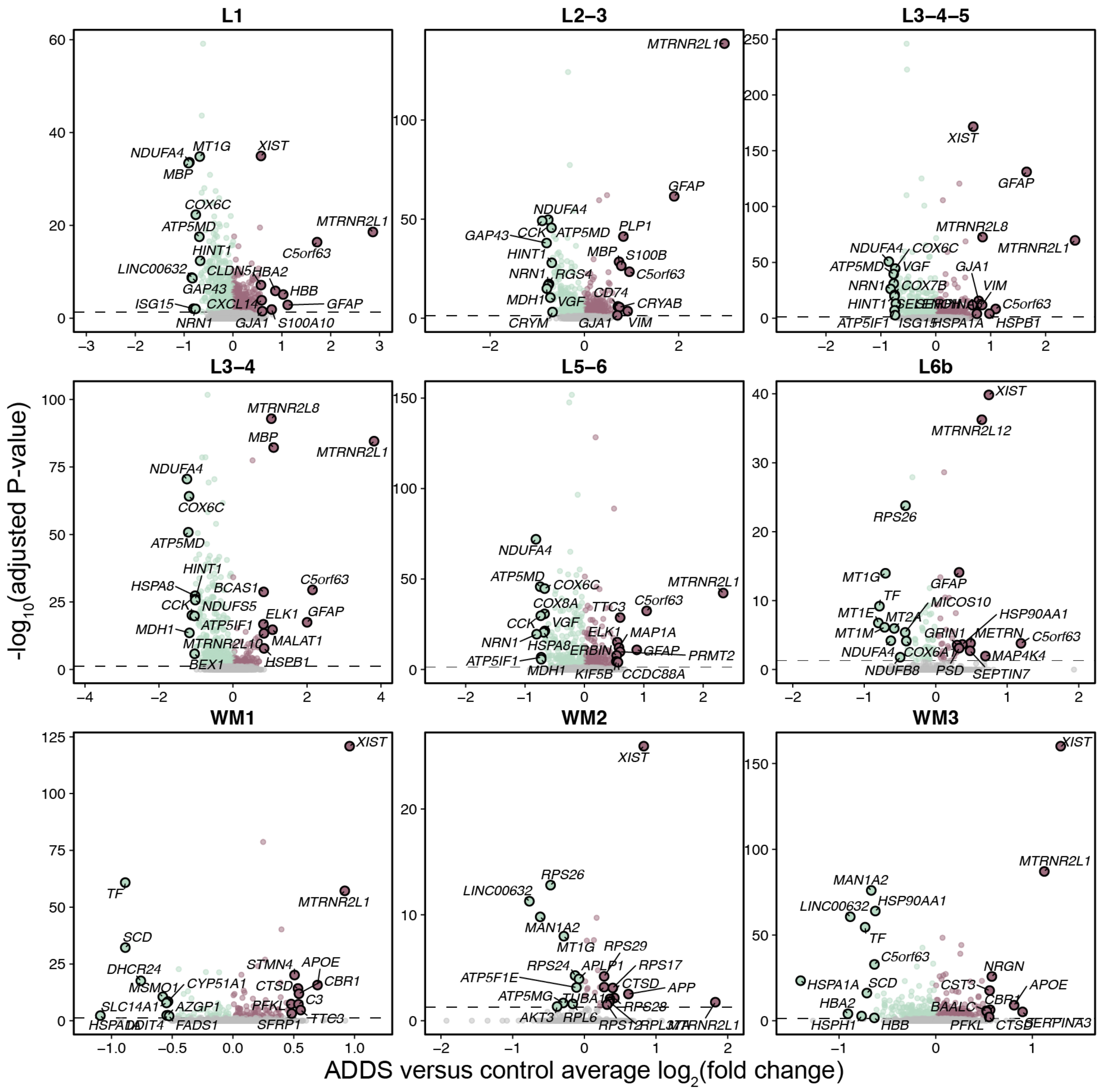

Supplementary Fig. 9 | AD in DS versus control DEGs in the ST dataset. Volcano plots show the adjusted significance levels and effect sizes from the differential gene expression comparisons between AD in DS cases versus cognitively normal controls in the human ST dataset. The results are shown for the differential gene expression analysis in each of the nine spatial clusters. The top and bottom ten genes by effect size are annotated. Statistical test: MAST [53], two-sided test.

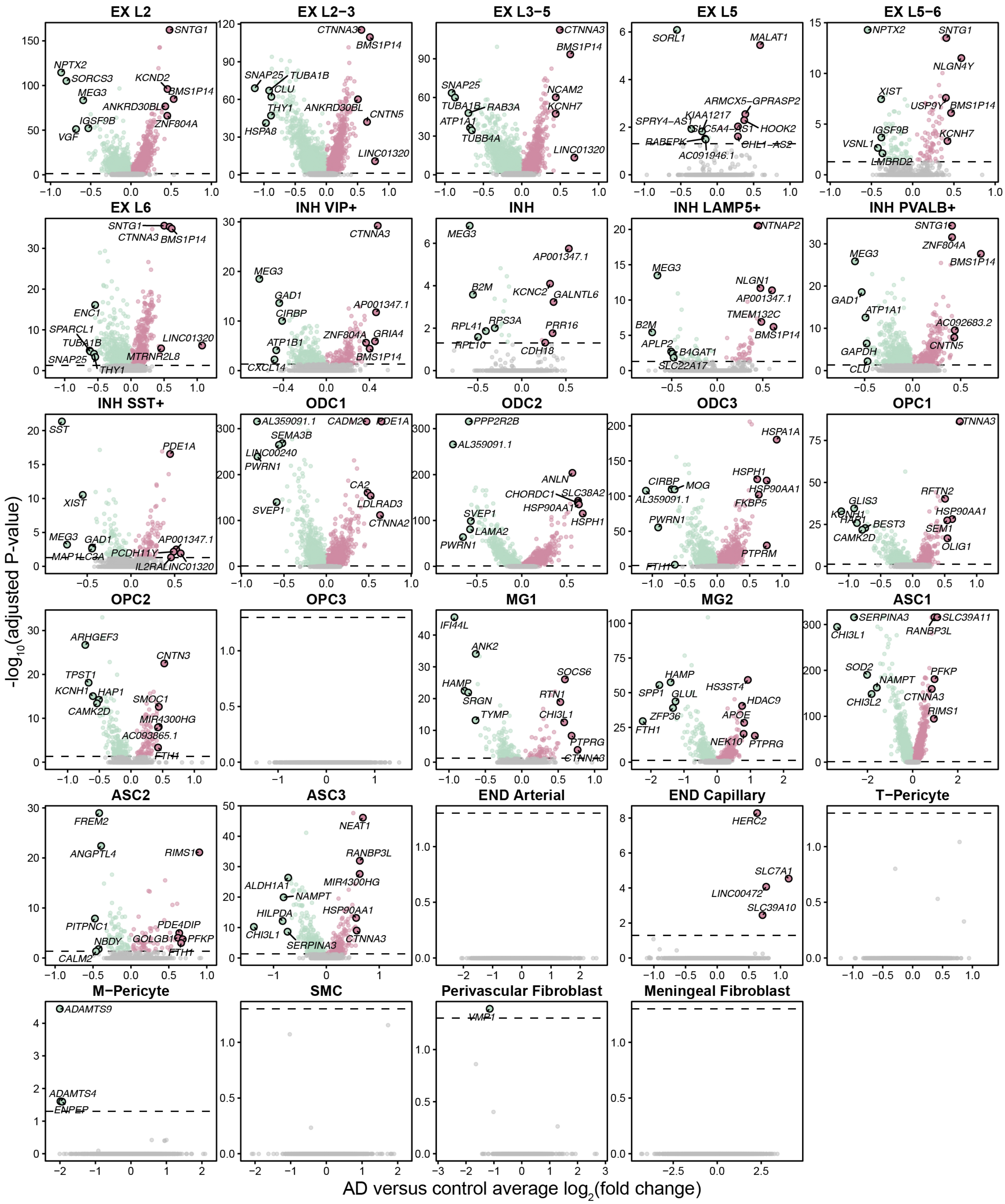

Supplementary Fig. 10 | Late-stage AD versus control DEGs in the snRNA-seq dataset. Volcano plots show the adjusted significance levels and effect sizes from the differential gene expression comparisons between late-stage AD cases versus cognitively normal controls from the integrated analysis of the three previously published snRNA-seq datasets. The results are shown for the differential gene expression analysis in each of the snRNA-seq clusters. The top and bottom five genes by effect size are annotated. Statistical test: MAST[53], two-sided test.

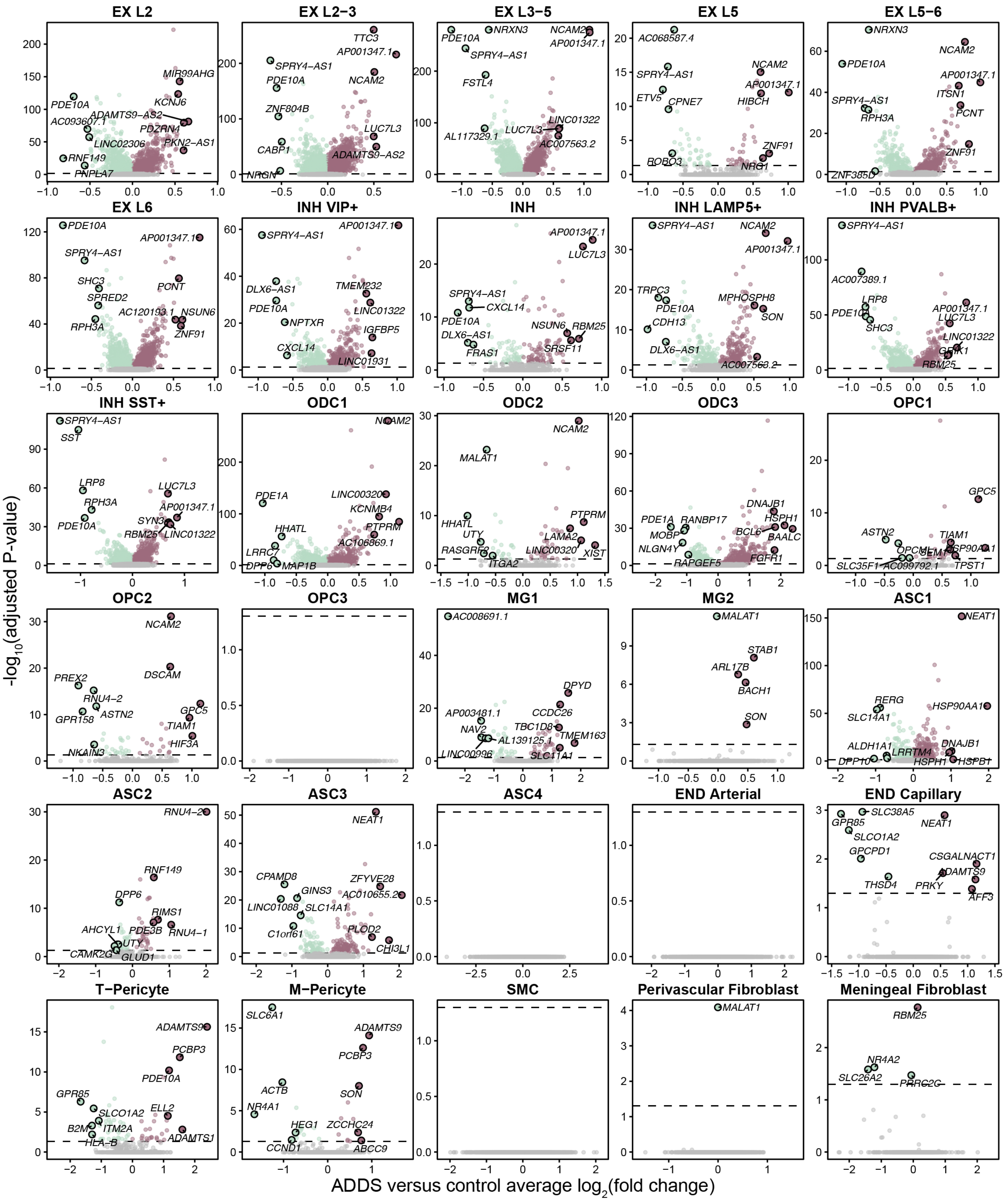

Supplementary Fig. 11 | AD in DS versus control DEGs in the FCX snRNA-seq dataset. Volcano plots show the adjusted significance levels and effect sizes from the differential gene expression comparisons between AD in DS cases versus cognitively normal controls in the frontal cortex (FCX) snRNA-seq dataset. The results are shown for the differential gene expression analysis in each of the snRNA-seq clusters. The top and bottom five genes by effect size are annotated. Statistical test: MAST[53], two-sided test.

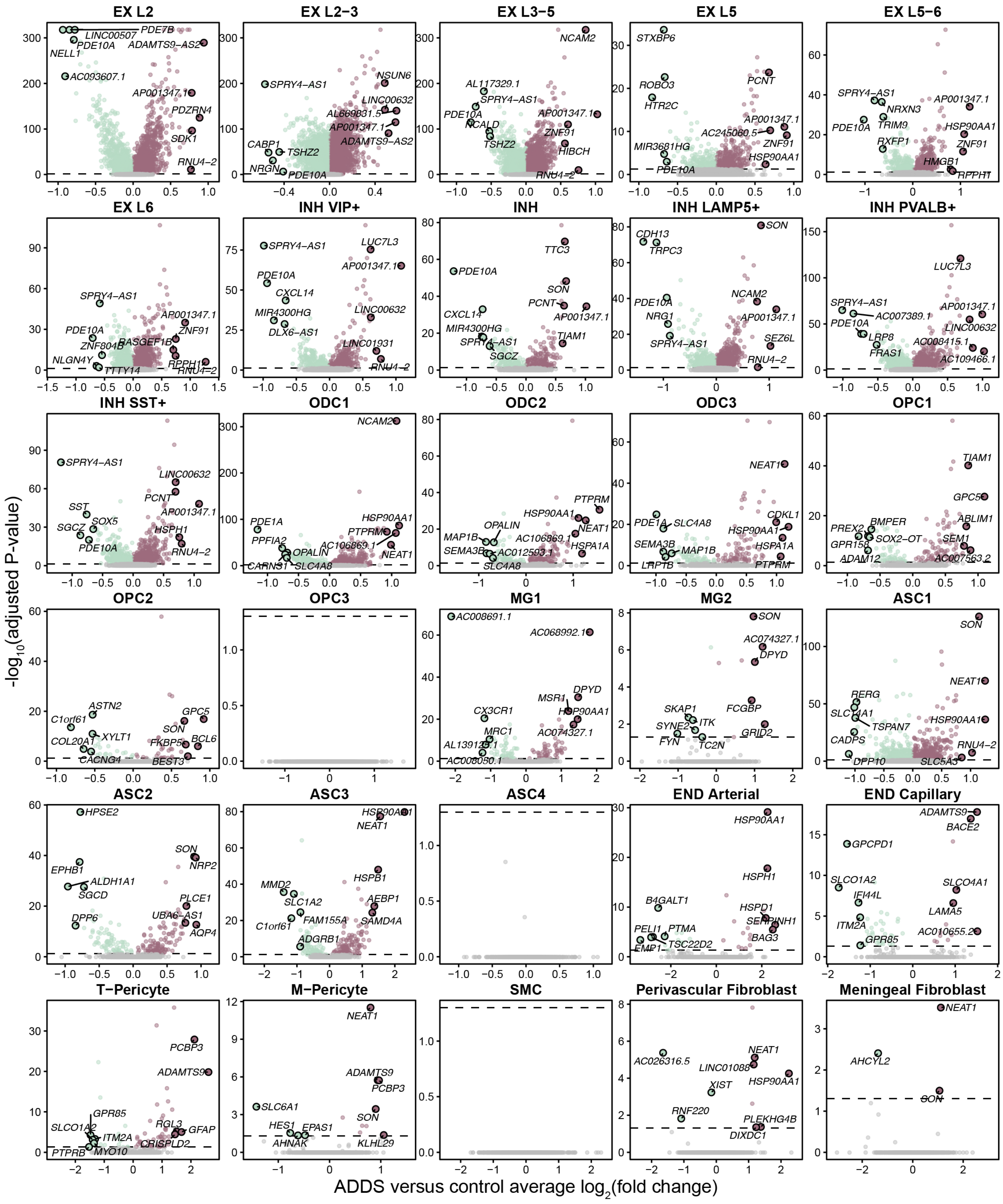

Supplementary Fig. 12 | AD in DS versus control DEGs in the PCC snRNA-seq dataset. Volcano plots show the adjusted significance levels and effect sizes from the differential gene expression comparisons between AD in DS cases versus cognitively normal controls in the posterior cingulate cortex (PCC) snRNA-seq dataset. The results are shown for the differential gene expression analysis in each of the snRNA-seq clusters. The top and bottom five genes by effect size are annotated. Statistical test: MAST [53], two-sided test.

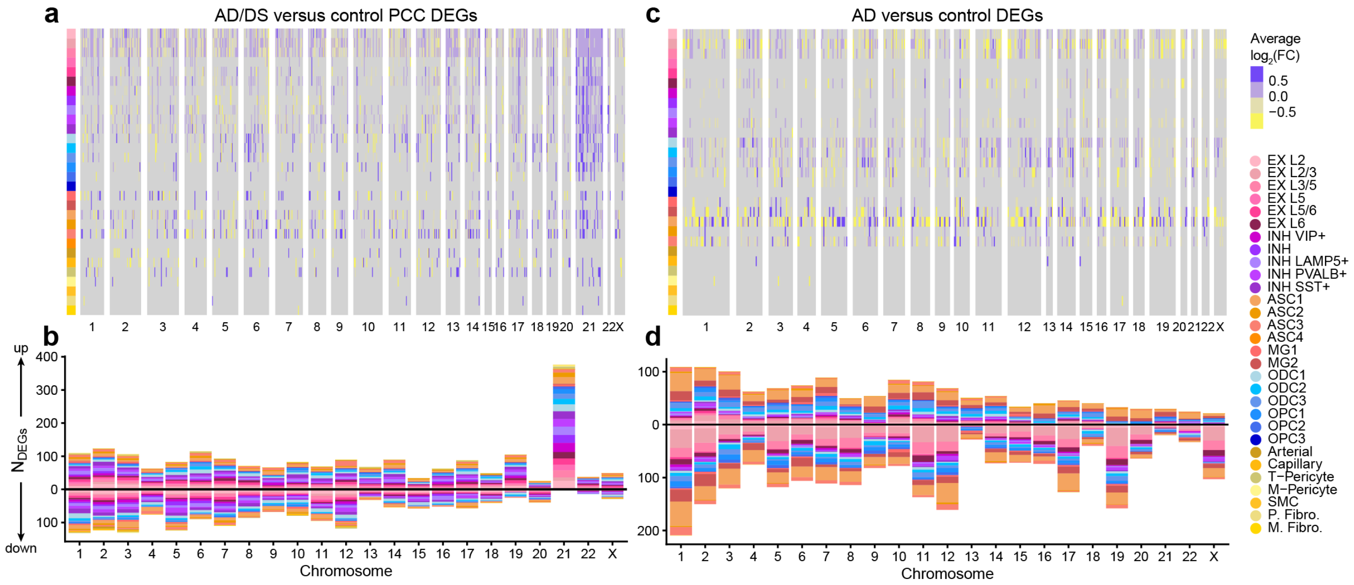

Supplementary Fig. 13 | snRNA-seq DEGs examined by chromosome. a, Heatmap colored by effect size from the PCC AD in DS versus control differential gene expression analysis, with genes stratified by chromosome and by spatial region. Statistically significant (FDR < 0.05) genes with an absolute average log2(fold-change) >= 0.25 in at least one region are shown. **b**, Stacked bar chart showing the number of PCC AD in DS versus control DEGs in each snRNA-seq cluster stratified by chromosome. **c**, Heatmap colored by effect size from the late-stage AD versus control differential gene expression analysis, with genes stratified by chromosome and by spatial region. Statistically significant (FDR < 0.05) genes with an absolute average log2(fold-change) >= 0.25 in at least one region are shown. **d**, Stacked bar chart showing the number of late-stage AD versus control DEGs in each snRNA-seq cluster stratified by chromosome.

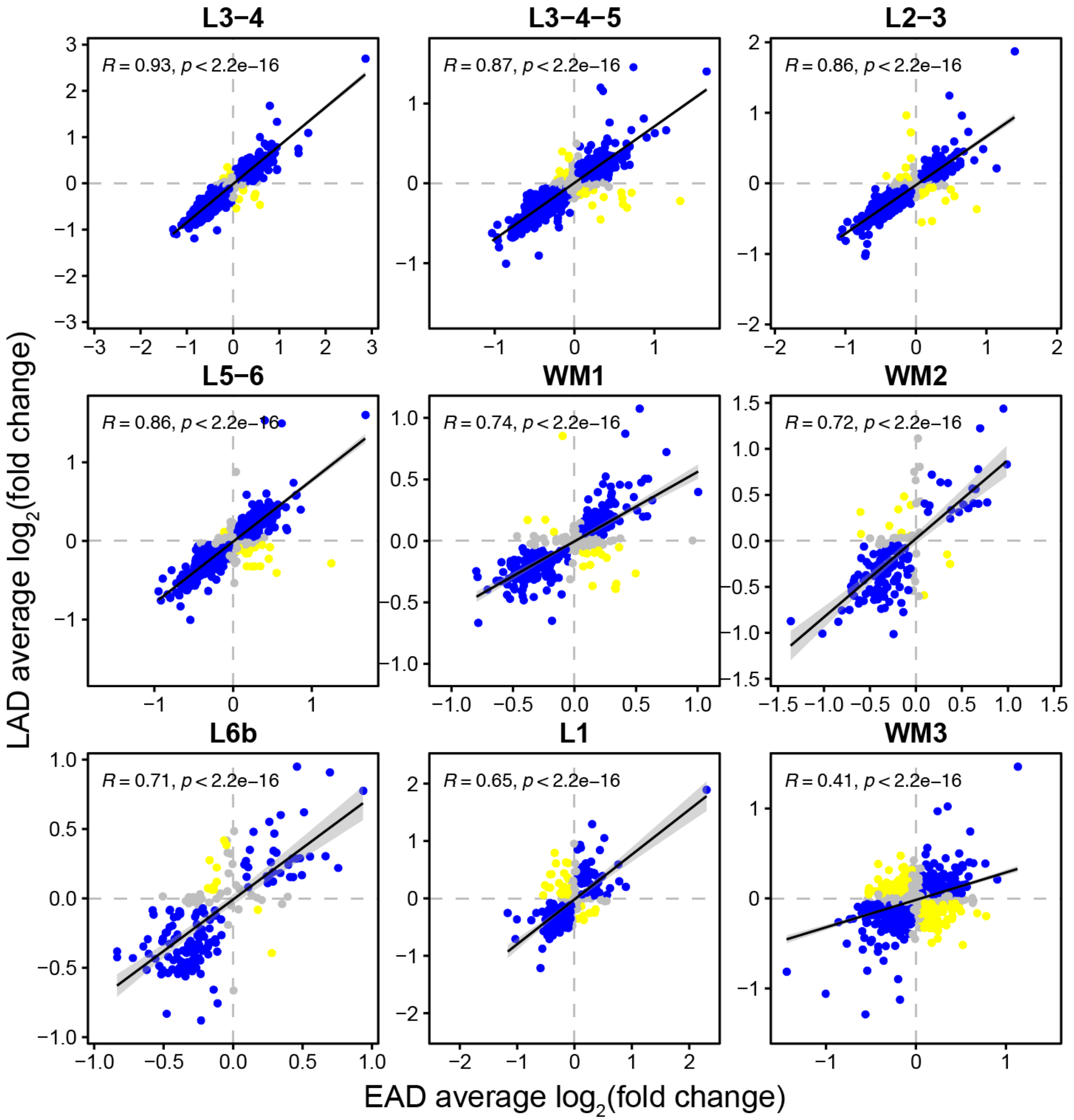

Supplementary Fig. 14 | Comparison of spatial transcriptomic DEG effect sizes between early-stage AD and late-stage AD. Comparison of differential expression effect sizes from early-stage AD versus control and late-stage AD versus control. Genes that were statistically significant (adjusted p-value < 0.05) in either comparison were included in this analysis. Genes are colored blue if the direction is consistent, yellow if inconsistent, and grey if the absolute effect sizes were smaller than 0.05. Black line represents a linear regression with a 95% confidence interval around the mean shown in grey. Pearson correlation coefficients are shown in the upper left corner of each panel.

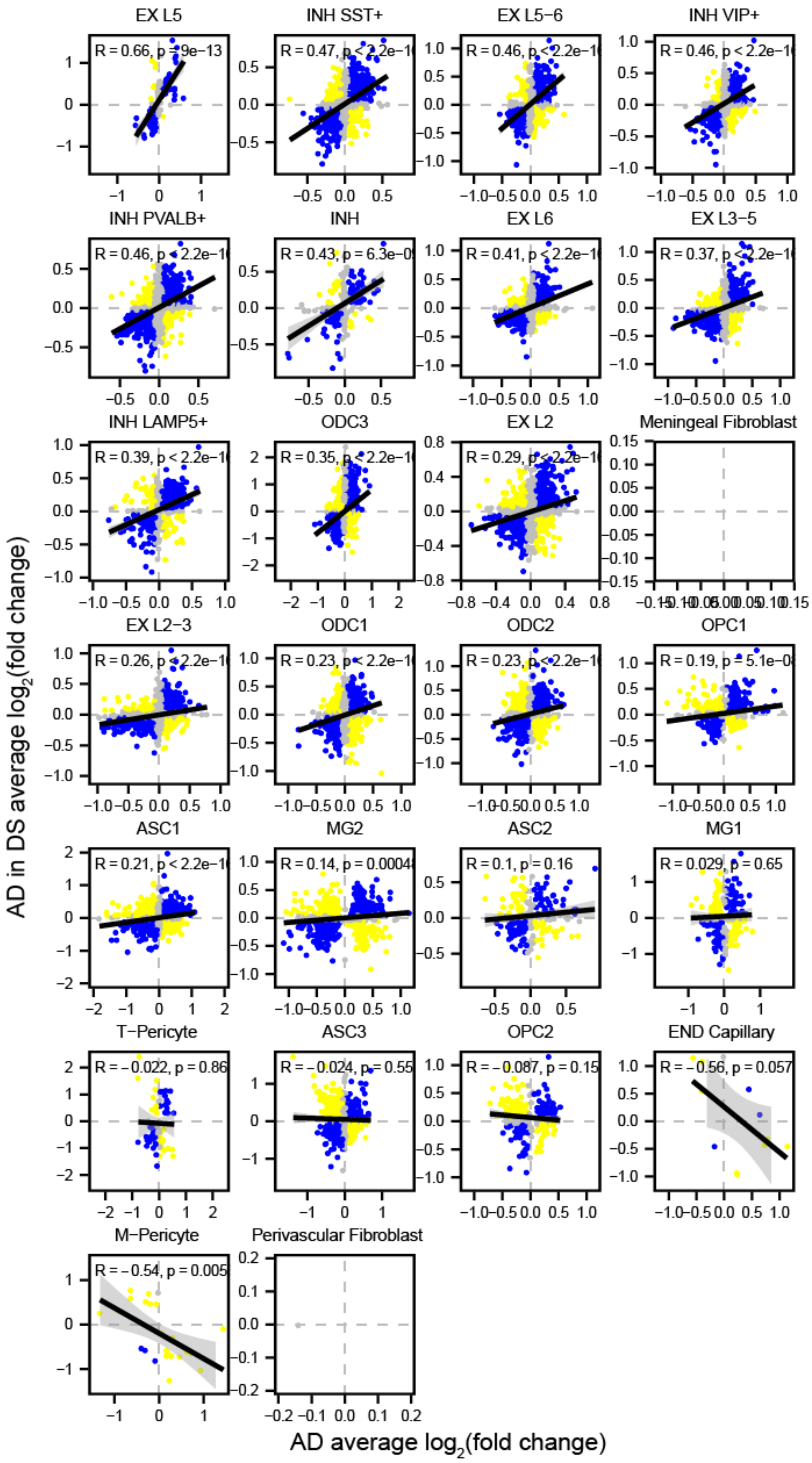

Supplementary Fig. 15 | Comparison of snRNA-seq DEG effect sizes between sporadic AD and AD in DS. Comparison of differential expression effect sizes from sAD versus control and AD in DS versus control. Genes that were statistically significant (adjusted p-value < 0.05) in either comparison were included in this analysis. Genes are colored blue if the direction is consistent, yellow if inconsistent, and grey if the absolute effect sizes were smaller than 0.05. Black line represents a linear regression with a 95% confidence interval around the mean shown in grey. Pearson correlation coefficients are shown in the upper left corner of each panel.

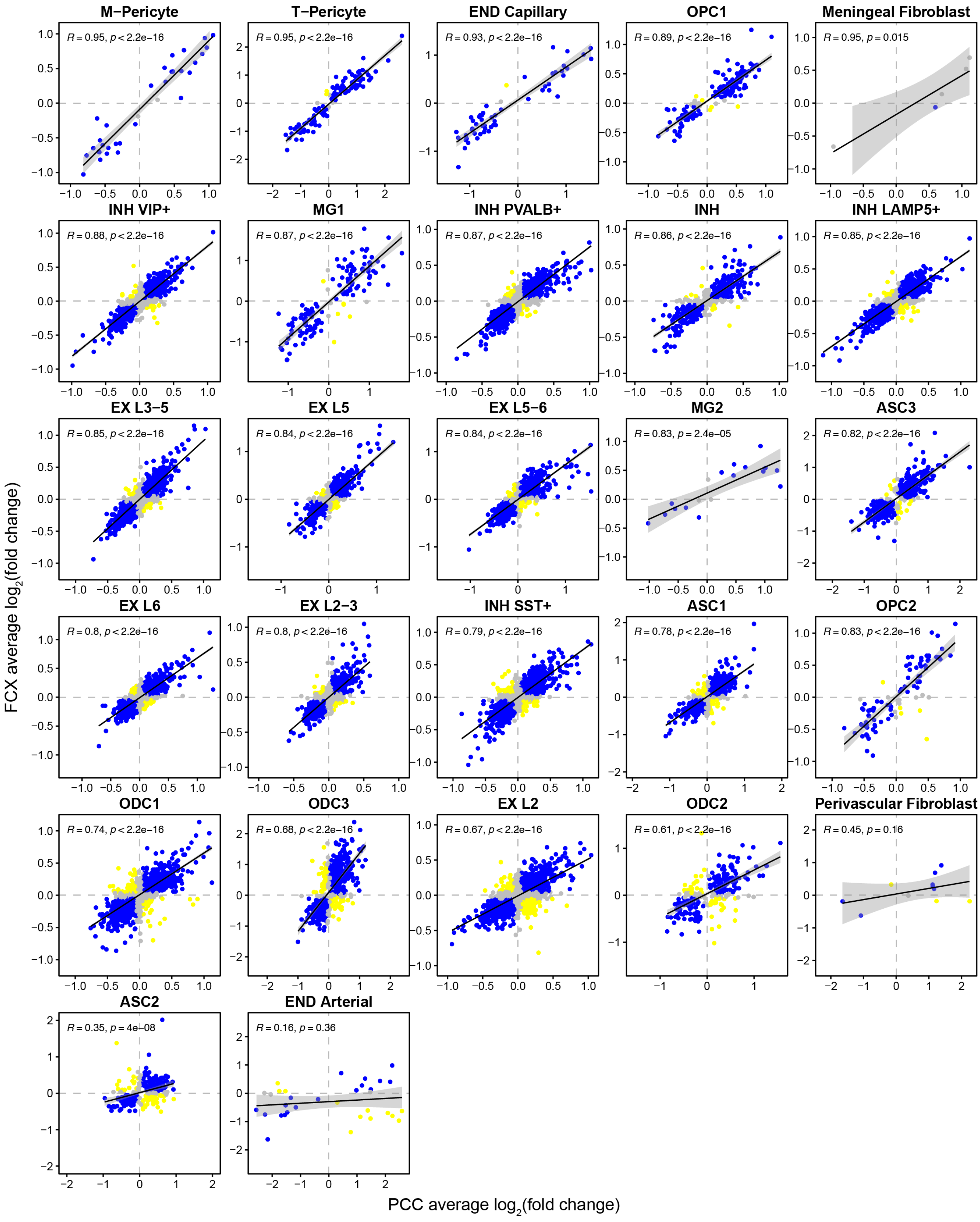

Supplementary Fig. 16 | Comparison of snRNA-seq AD in DS versus control DEG effect sizes in the PCC and FCX. Comparison of differential expression effect sizes from AD in DS versus control in the PCC and FCX snRNA-seq data. Genes that were statistically significant (adjusted p-value < 0.05) in either comparison were included in this analysis. Genes are colored blue if the direction is consistent, yellow if inconsistent, and grey if the absolute effect sizes were smaller than 0.05. Black line represents a linear regression with a 95% confidence interval shown in grey. Pearson correlation coefficients are shown in the upper left corner of each panel.

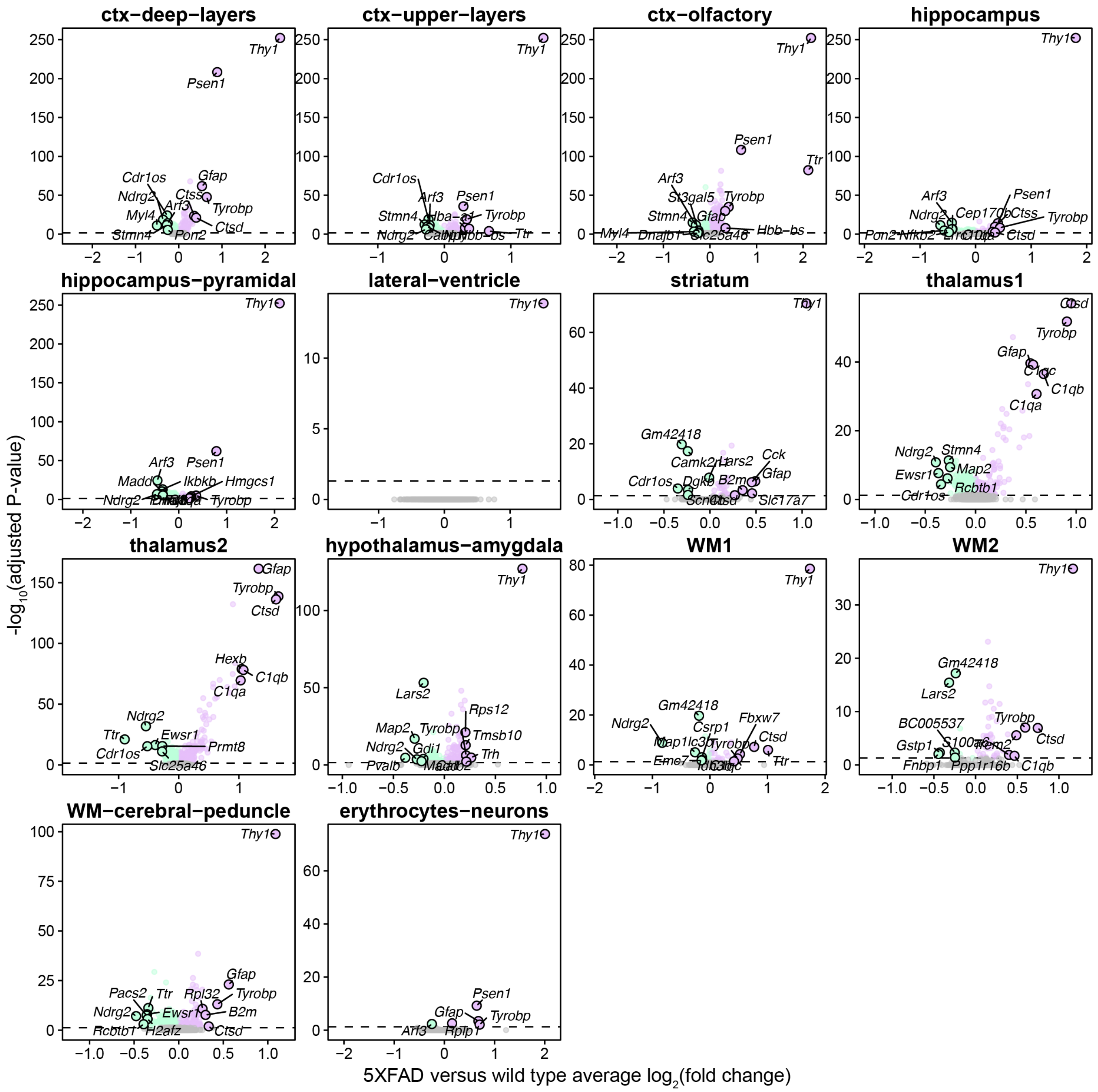

Supplementary Fig. 17 | 5xFAD versus wild type DEGs at four months of age. Volcano plots show the adjusted significance levels and effect sizes from the differential gene expression comparisons between 5xFAD versus wild type mice at four months of age. The results are shown for the differential gene expression analysis in each of the spatial transcriptomic clusters. The top and bottom six genes by effect size are annotated. Statistical test: MAST[53], two-sided test.

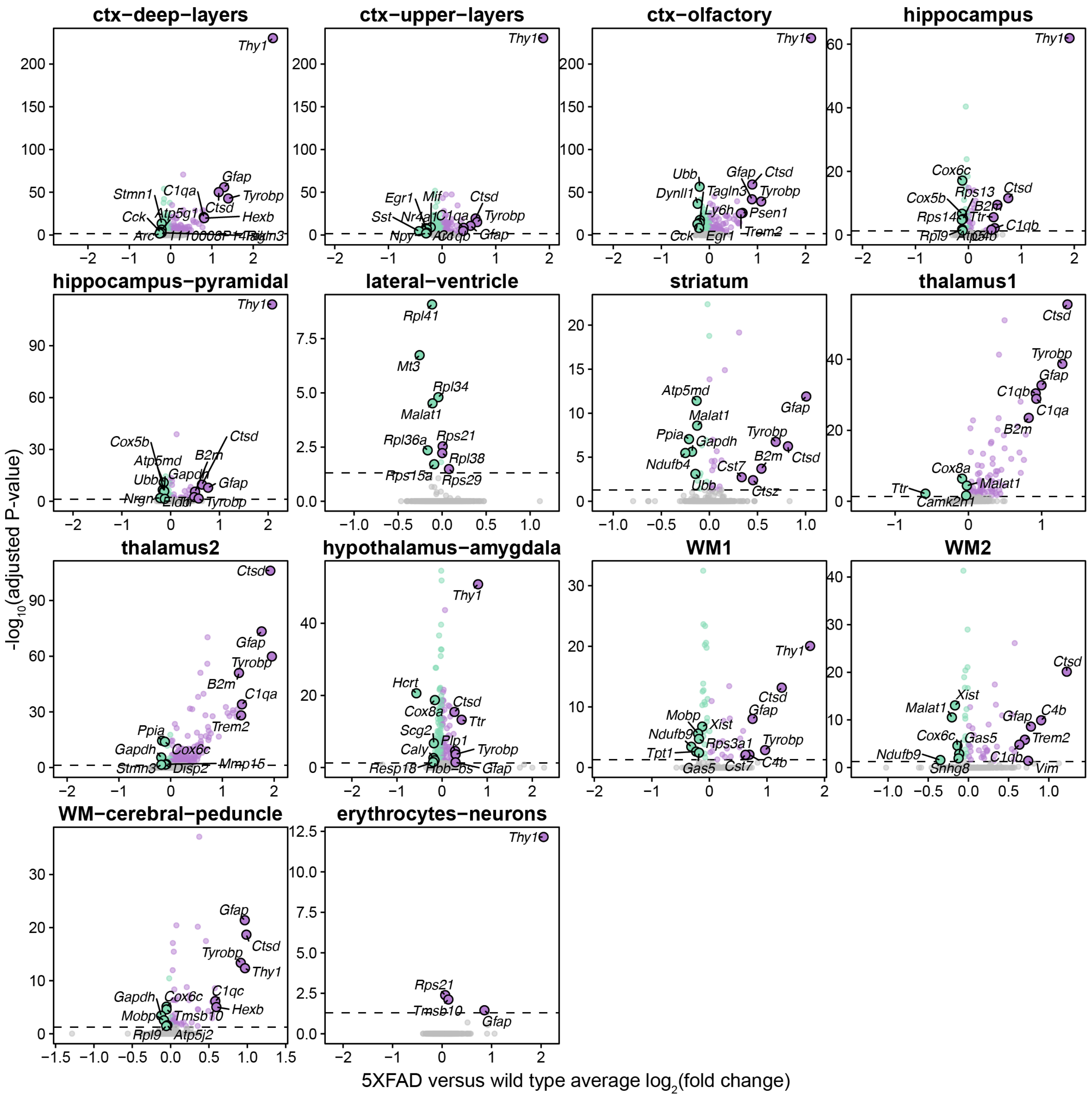

Supplementary Fig. 18 | 5xFAD versus wild type DEGs at six months of age. Volcano plots show the adjusted significance levels and effect sizes from the differential gene expression comparisons between 5xFAD versus wild type mice at six months of age. The results are shown for the differential gene expression analysis in each of the spatial transcriptomic clusters. The top and bottom six genes by effect size are annotated. Statistical test: MAST[53], two-sided test.

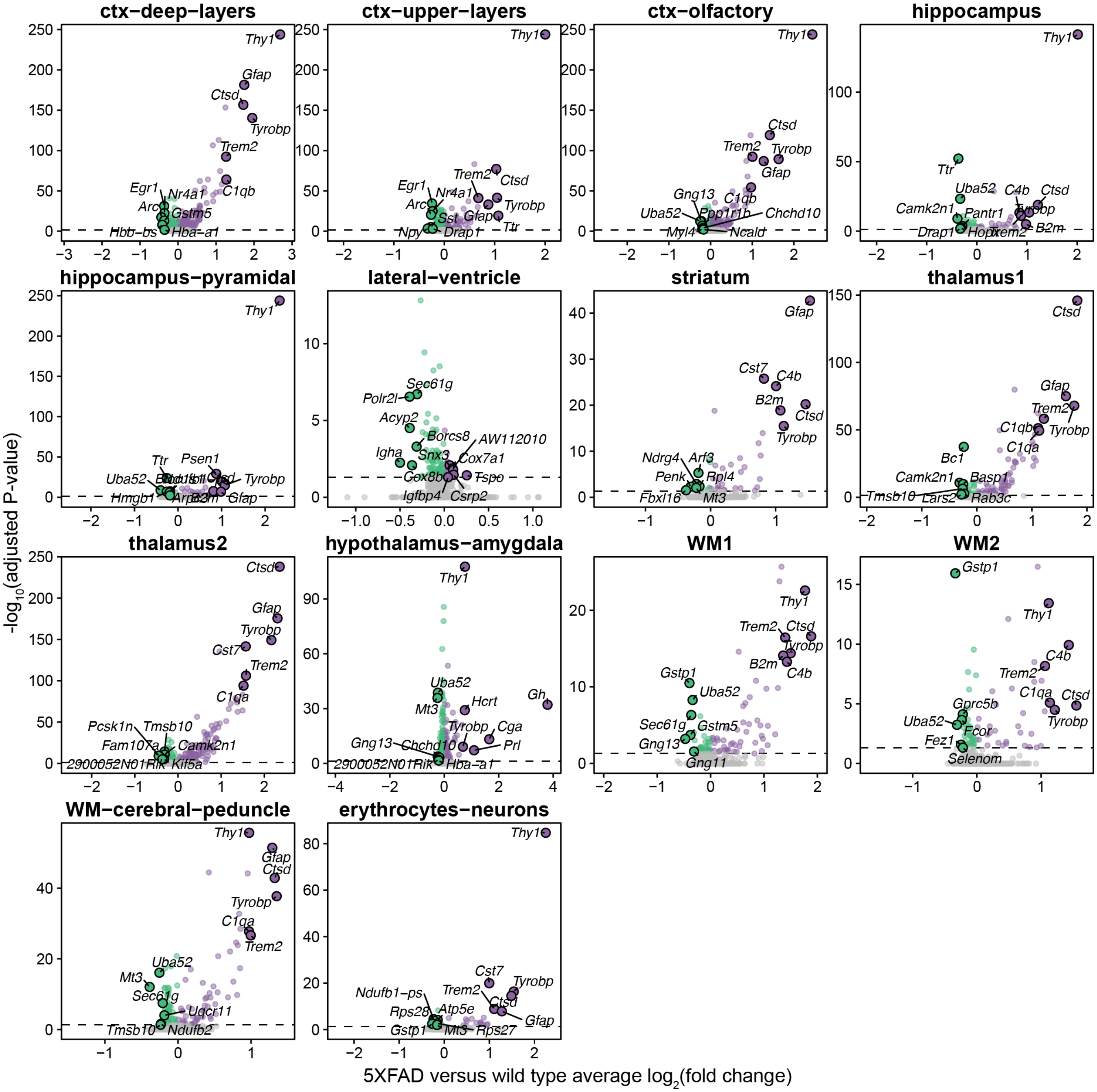

Supplementary Fig. 19 | 5xFAD versus wild type DEGs at eight months of age. Volcano plots show the adjusted significance levels and effect sizes from the differential gene expression comparisons between 5xFAD versus wild type mice at six months of age. The results are shown for the differential gene expression analysis in each of the spatial transcriptomic clusters. The top and bottom six genes by effect size are annotated. Statistical test: MAST[53], two-sided test.

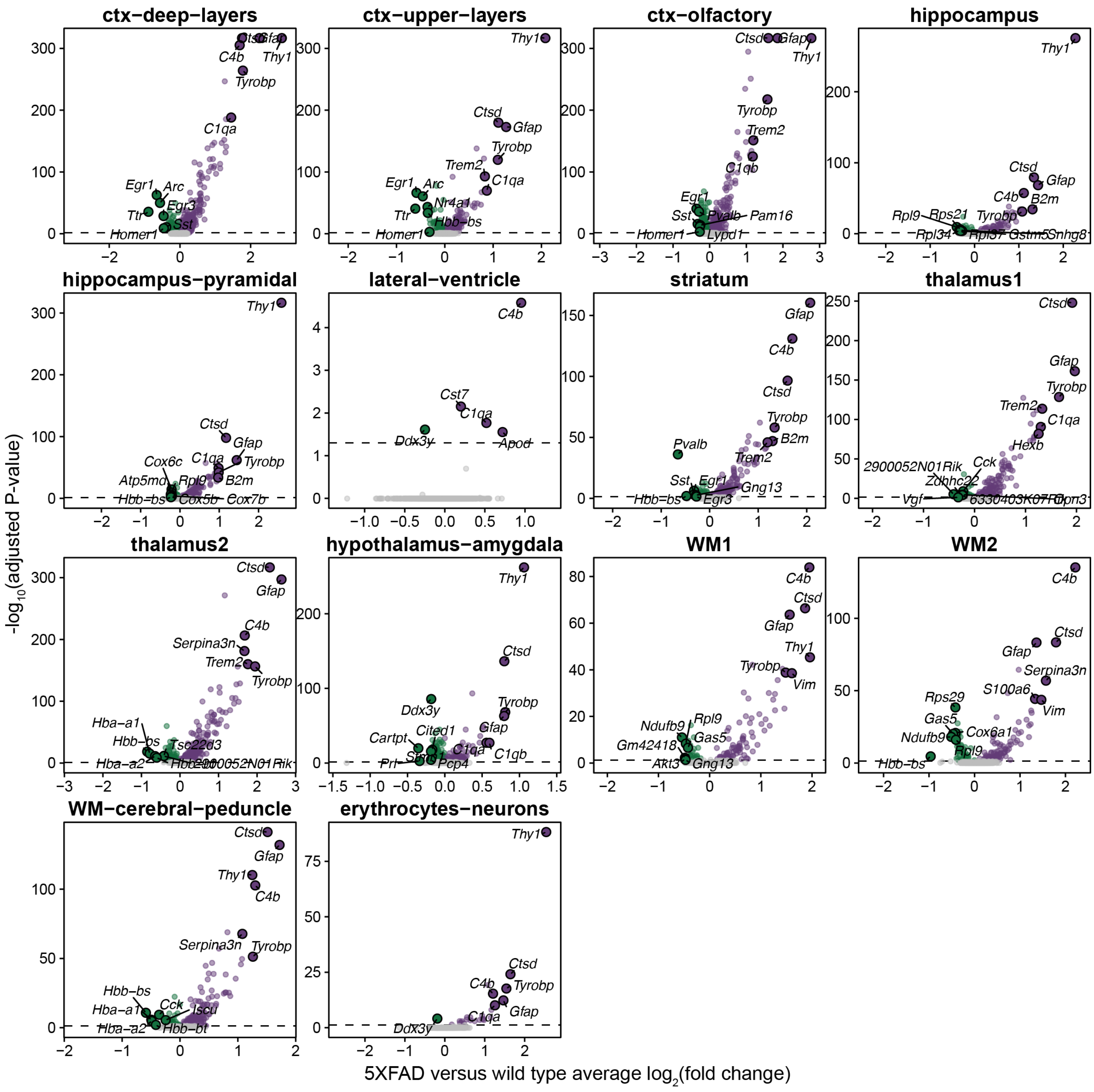

Supplementary Fig. 20 | 5xFAD versus wild type DEGs at twelve months of age. Volcano plots show the adjusted significance levels and effect sizes from the differential gene expression comparisons between 5xFAD versus wild type mice at six months of age. The results are shown for the differential gene expression analysis in each of the spatial transcriptomic clusters. The top and bottom six genes by effect size are annotated. Statistical test: MAST[53], two-sided test.

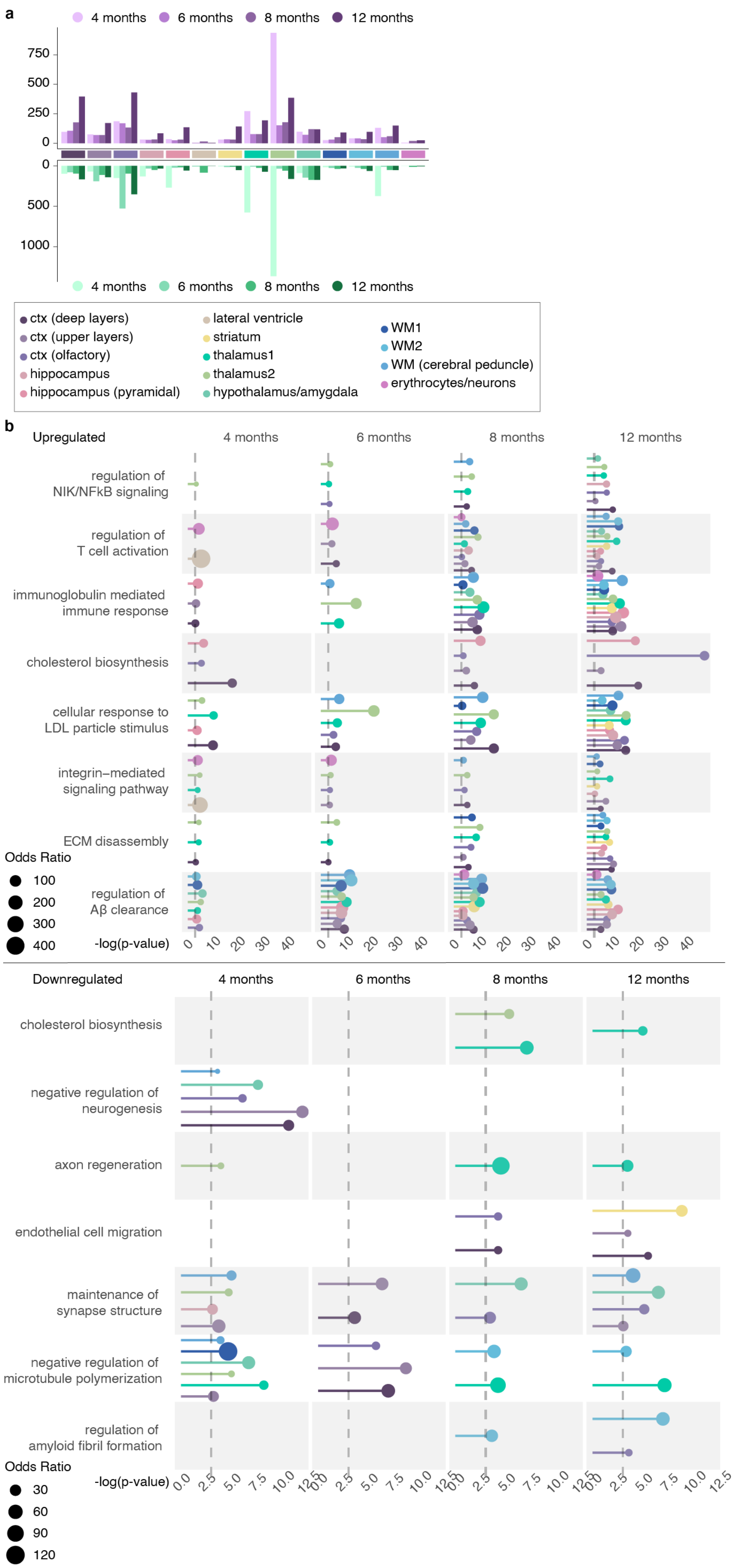

Supplementary Fig. 21 | Number of 5xFAD DEGs and GO term enrichment analysis. a, Barplot showing the number of DEGs in each group. **b**, Selected pathway enrichment analysis for the 5xFAD DEGs. One-sided Fisher's exact test was used for enrichment analysis.

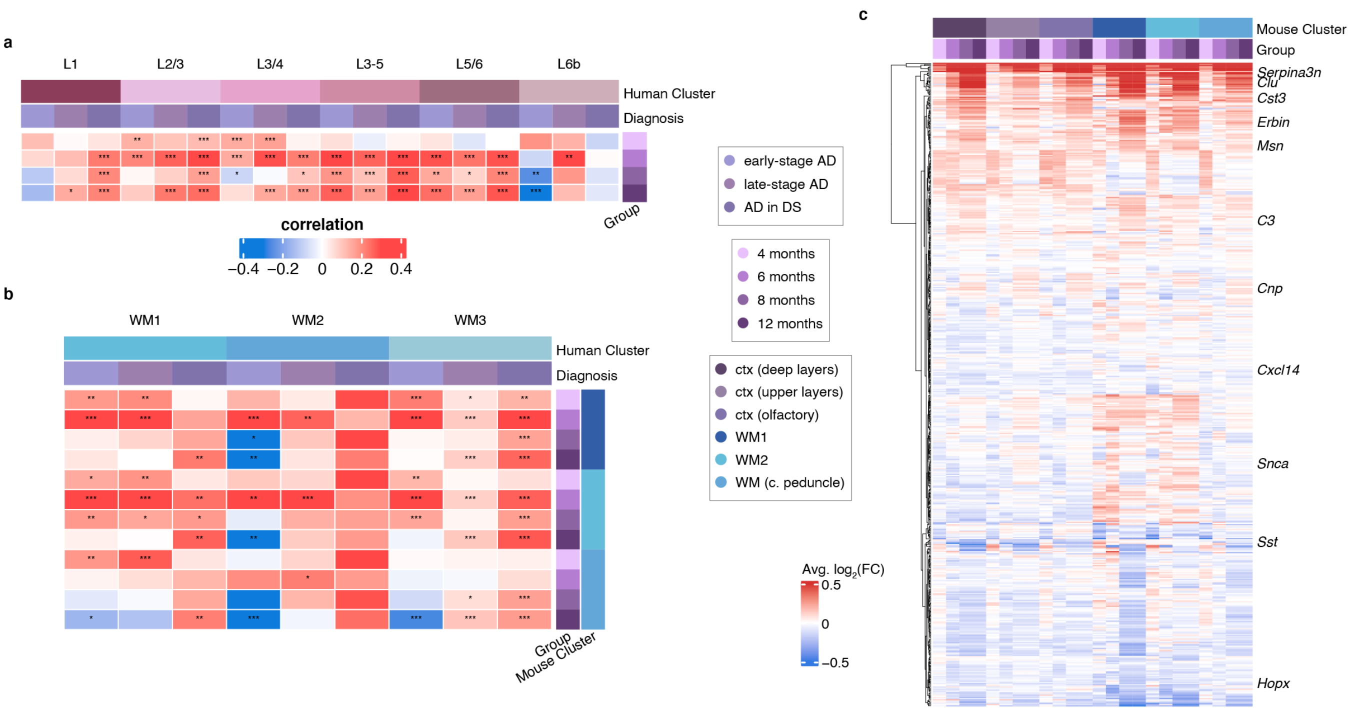

Supplementary Fig. 22 | **Comparisons of human and mouse DEGs. a-b**, Heatmaps showing correlations of effect sizes between human and mouse ST datasets in orthologous genes between human and mouse for cortical layer clusters **a** and white matter **b** clusters. **c**, Heatmap showing differential gene expression effect size results in the 5xFAD versus WT comparisons.

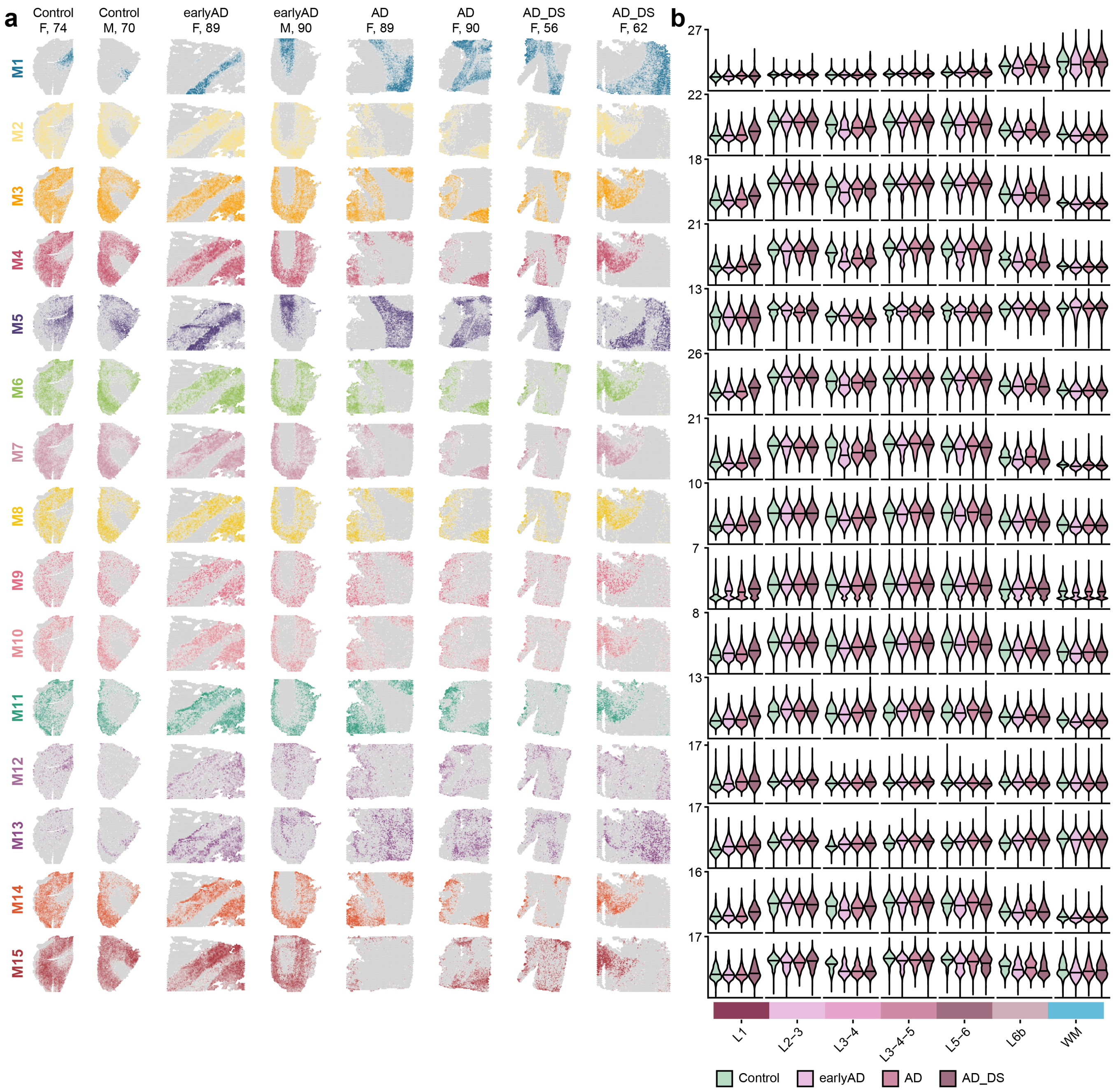

Supplementary Fig. 23 | Module eigengenes for the human ST meta-modules. a, Spatial feature plots showing module eigengenes (MEs) for the 15 human co-expression meta-modules shown in eight representative samples. **b**, ME distributions in each disease group (control, early-stage AD, late-stage AD, AD in DS) stratified by cortical layer clusters and white matter.

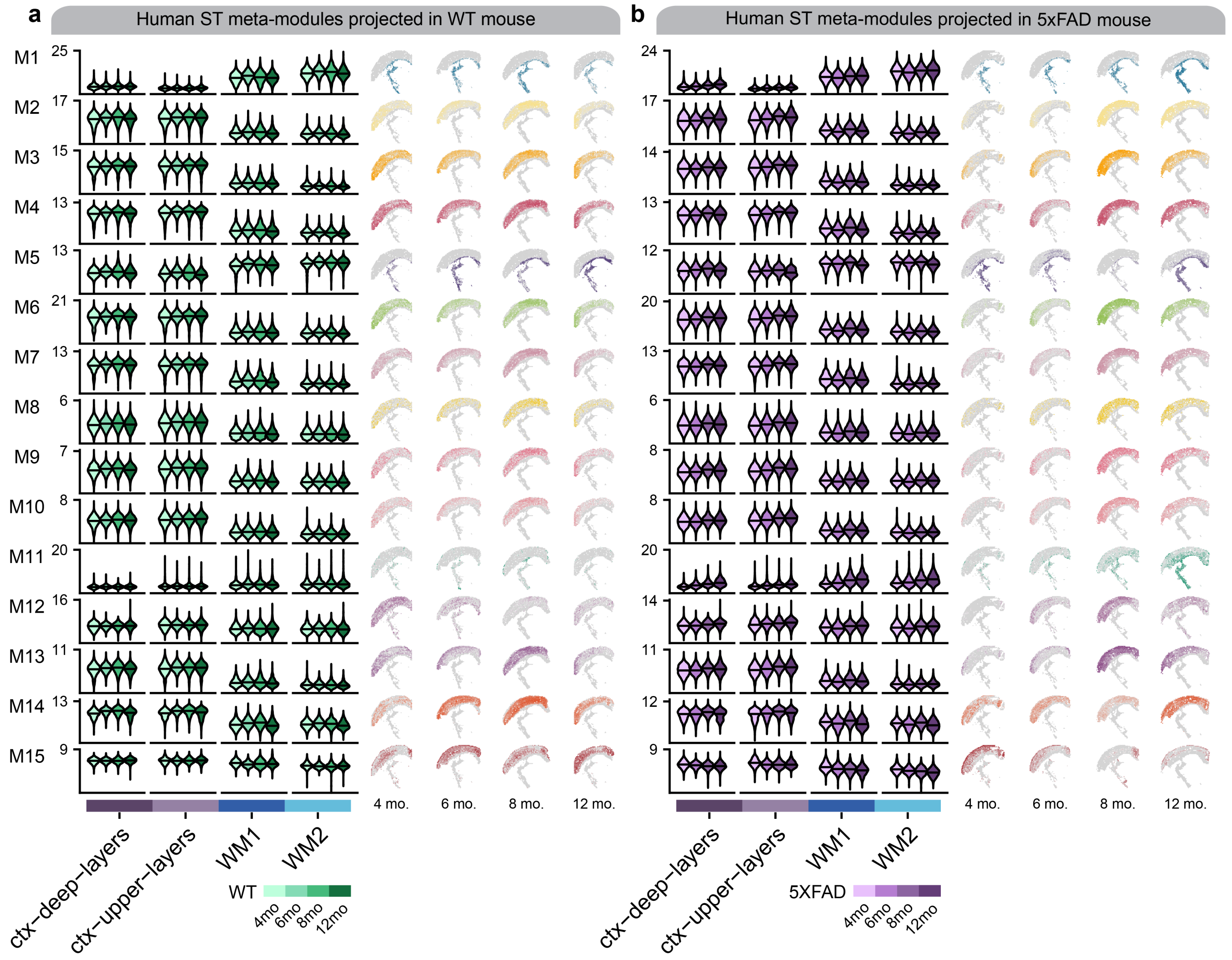

Supplementary Fig. 24 | Module eigengenes of human co-expression meta-modules in the mouse ST dataset. a-b, Left: Module eigengene (ME) distributions for the 15 human co-expression meta-modules in each mouse age group (control, early-stage AD, late-stage AD, AD in DS) stratified by cortical and white matter clusters. Right: Spatial feature plots showing MEs in four representative samples. Panel **a** shows the results in wild type mice and panel **b** shows the results in 5xFAD.

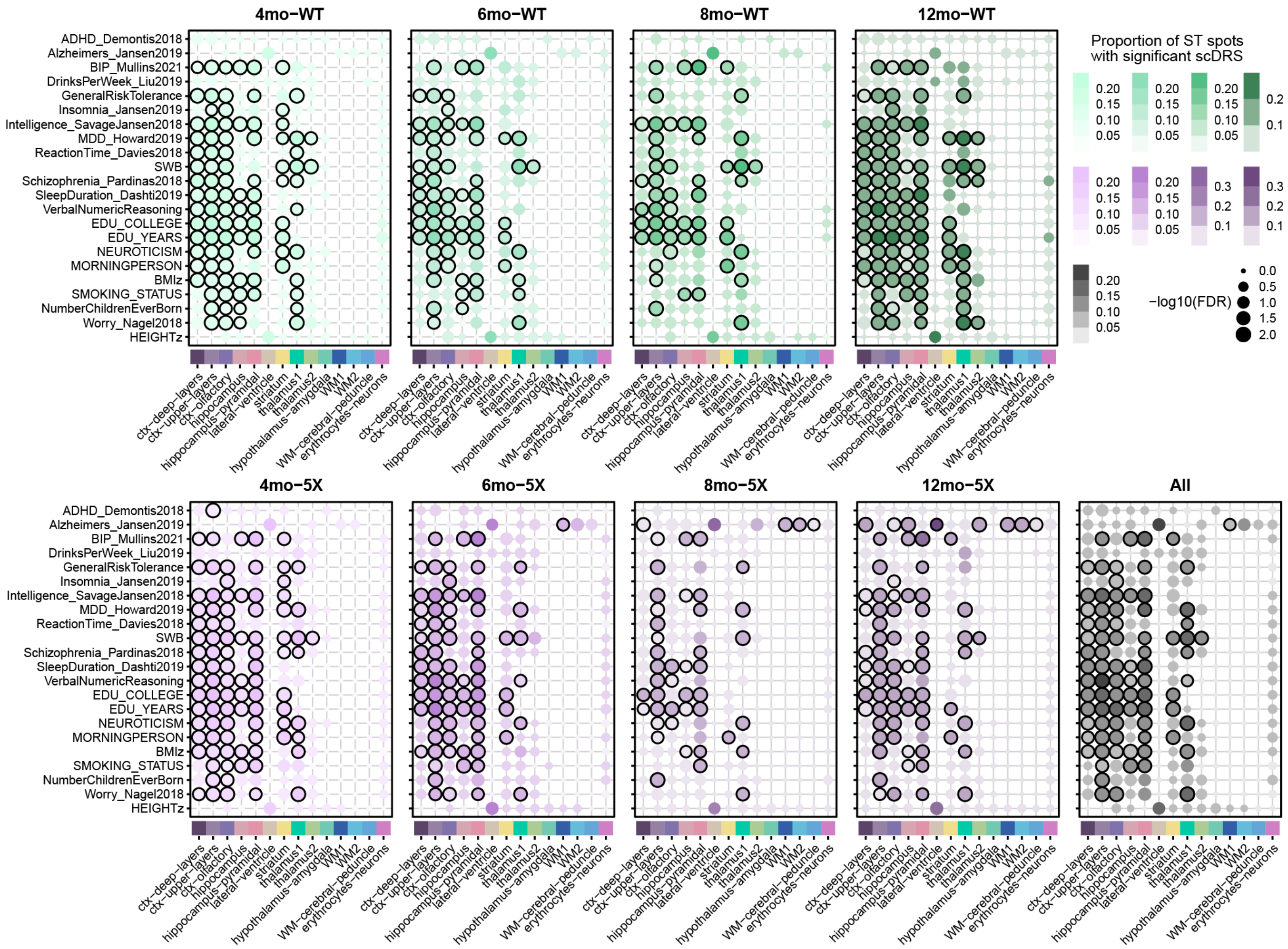

Supplementary Fig. 25 | Genetic enrichment analysis in mouse Spatial Transcriptomics. Dot plots showing the results of genetic enrichment analysis performed in the mouse Visium ST dataset using scDRS[44]. The scDRS Python package was run on the mouse ST dataset to compute spatial transcriptomic disease relevance scores (st-DRS) across a corpus of 22 traits provided by the scDRS package, resulting in spot-level disease enrichment scores and significance levels. A Monte Carlo (MC) test was used to test for group-level significance between each trait and the ST clusters, separately for each disease group and for the entire dataset together (All, right side). Black outlines on the dots denote a significant group-level association (FDR $\leq$ 0.05).

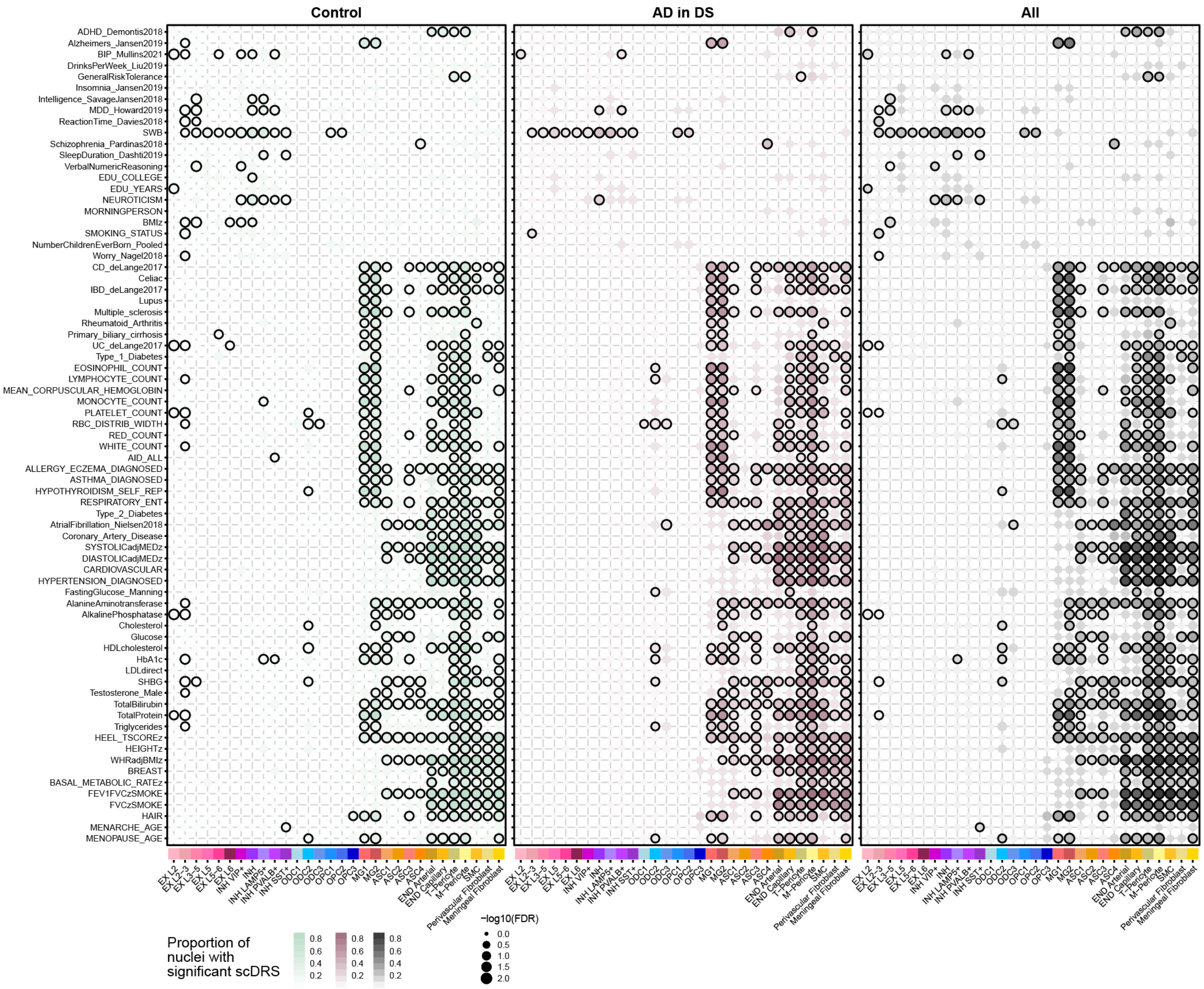

Supplementary Fig. 26 | Genetic enrichment analysis in the AD in DS snRNA-seq dataset. Dot plots showing the results of genetic enrichment analysis performed in the snRNA-seq dataset generated in this study using scDRS[44]. The scDRS Python package was run on the snRNA-seq dataset to compute single-cell disease relevance scores (sc-DRS) across a corpus of 74 traits provided by the scDRS package, resulting in cell-level disease enrichment scores and significance levels. A Monte Carlo (MC) test was used to test for group-level significance between each trait and the snRNA-seq clusters, separately for each disease group and for the entire dataset together (All, right side). Black outlines on the dots denote a significant group-level association (FDR $\leq$ 0.05).

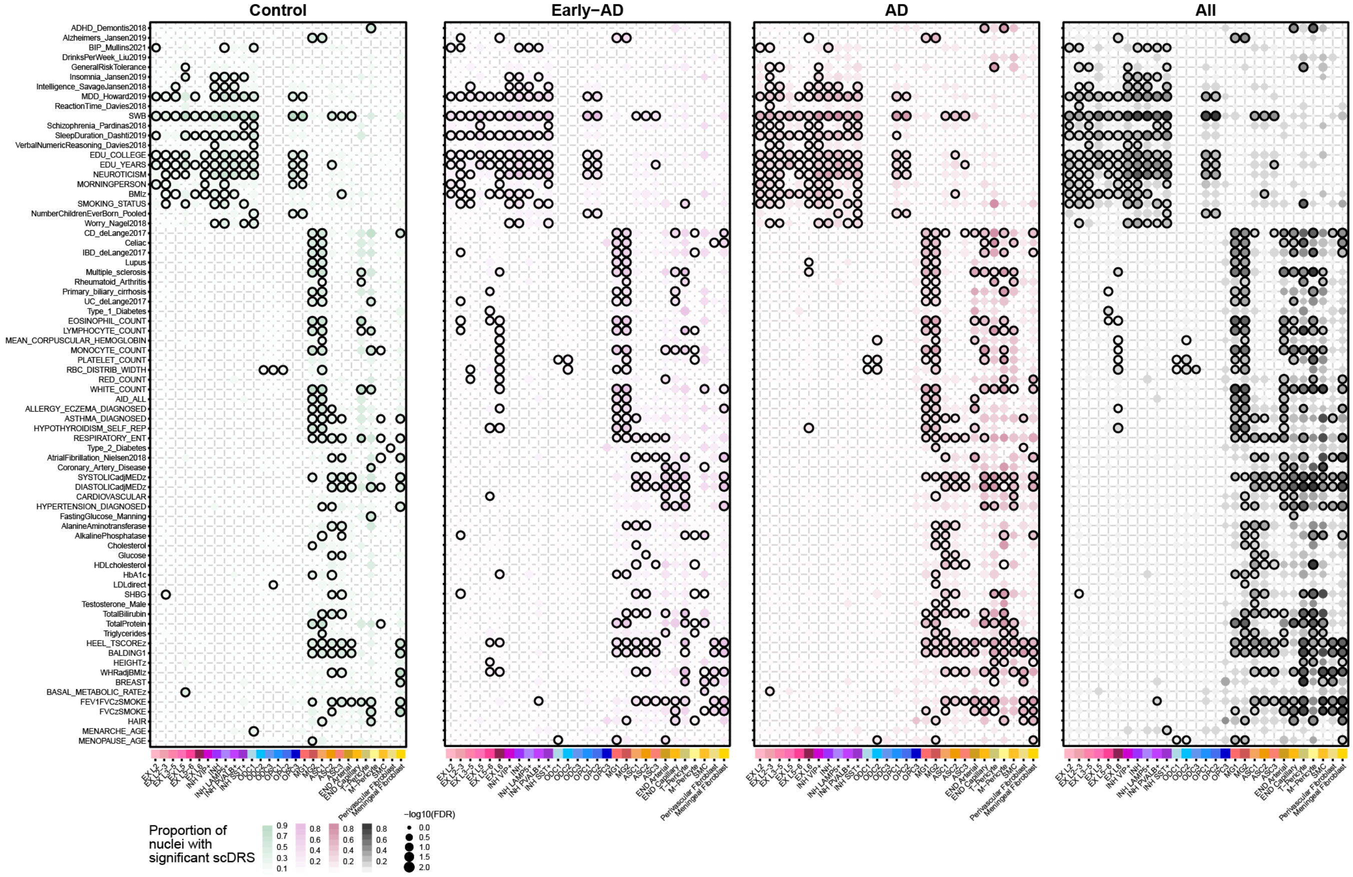

Supplementary Fig. 27 | Genetic enrichment analysis in the Mathys et al [54] snRNA-seq dataset. Dot plots showing the results of genetic enrichment analysis performed in the Mathys et al [54] dataset generated in this study using scDRS [44]. The scDRS Python package was run on the snRNA-seq dataset to compute single-cell disease relevance scores (sc-DRS) across a corpus of 74 traits provided by the scDRS package, resulting in cell-level disease enrichment scores and significance levels. A Monte Carlo (MC) test was used to test for group-level significance between each trait and the snRNA-seq clusters, separately for each disease group and for the entire dataset together (All, right side). Black outlines on the dots denote a significant group-level association (FDR ≤ 0.05).

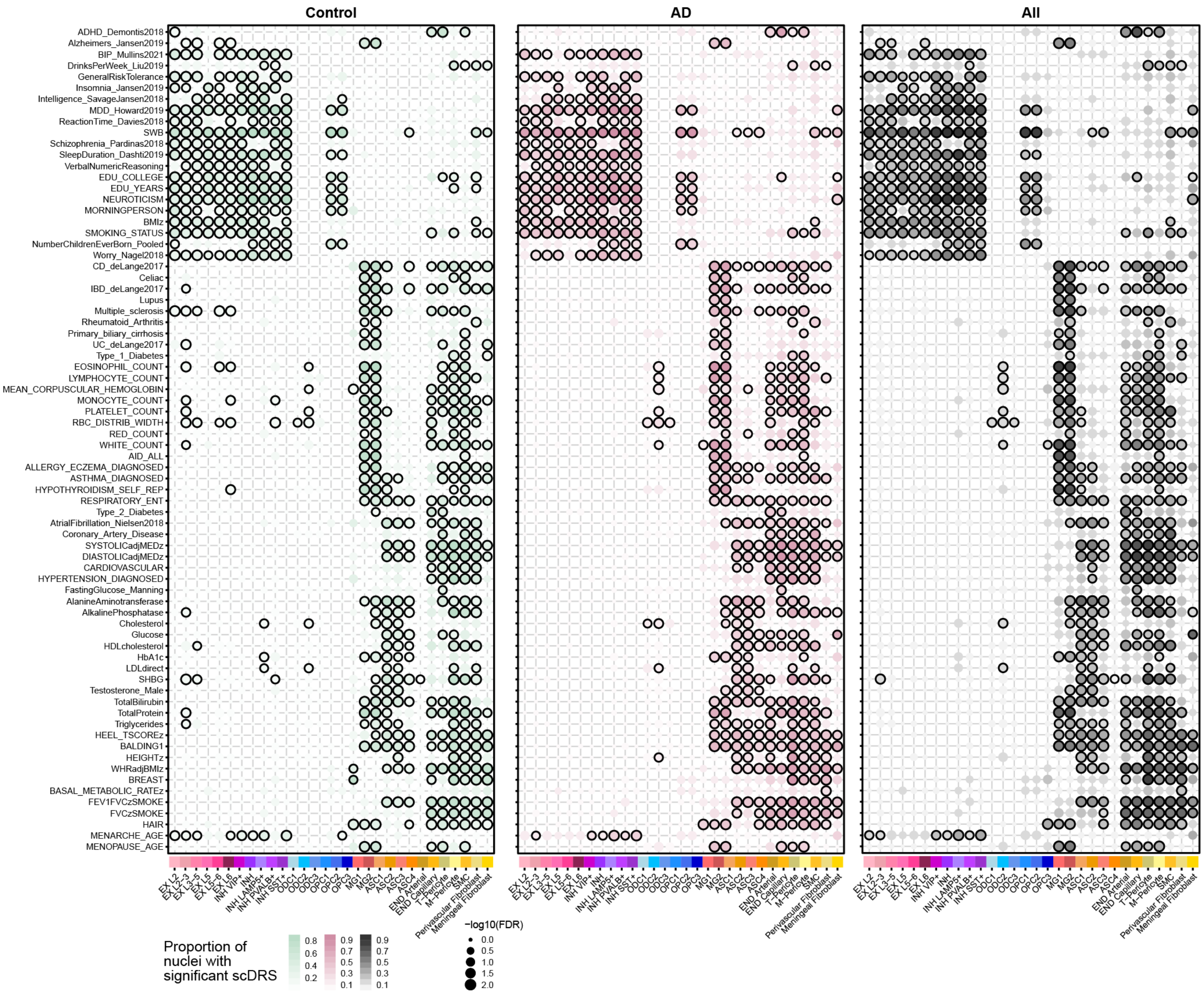

Supplementary Fig. 28 | Genetic enrichment analysis in the Zhou et al[48] snRNA-seq dataset. Dot plots showing the results of genetic enrichment analysis performed in the Zhou et al[48] dataset generated in this study using scDRS[44]. The scDRS Python package was run on the snRNA-seq dataset to compute single-cell disease relevance scores (sc-DRS) across a corpus of 74 traits provided by the scDRS package, resulting in cell-level disease enrichment scores and significance levels. A Monte Carlo (MC) test was used to test for group-level significance between each trait and the snRNA-seq clusters, separately for each disease group and for the entire dataset together (All, right side). Black outlines on the dots denote a significant group-level association (FDR $\leq$ 0.05).

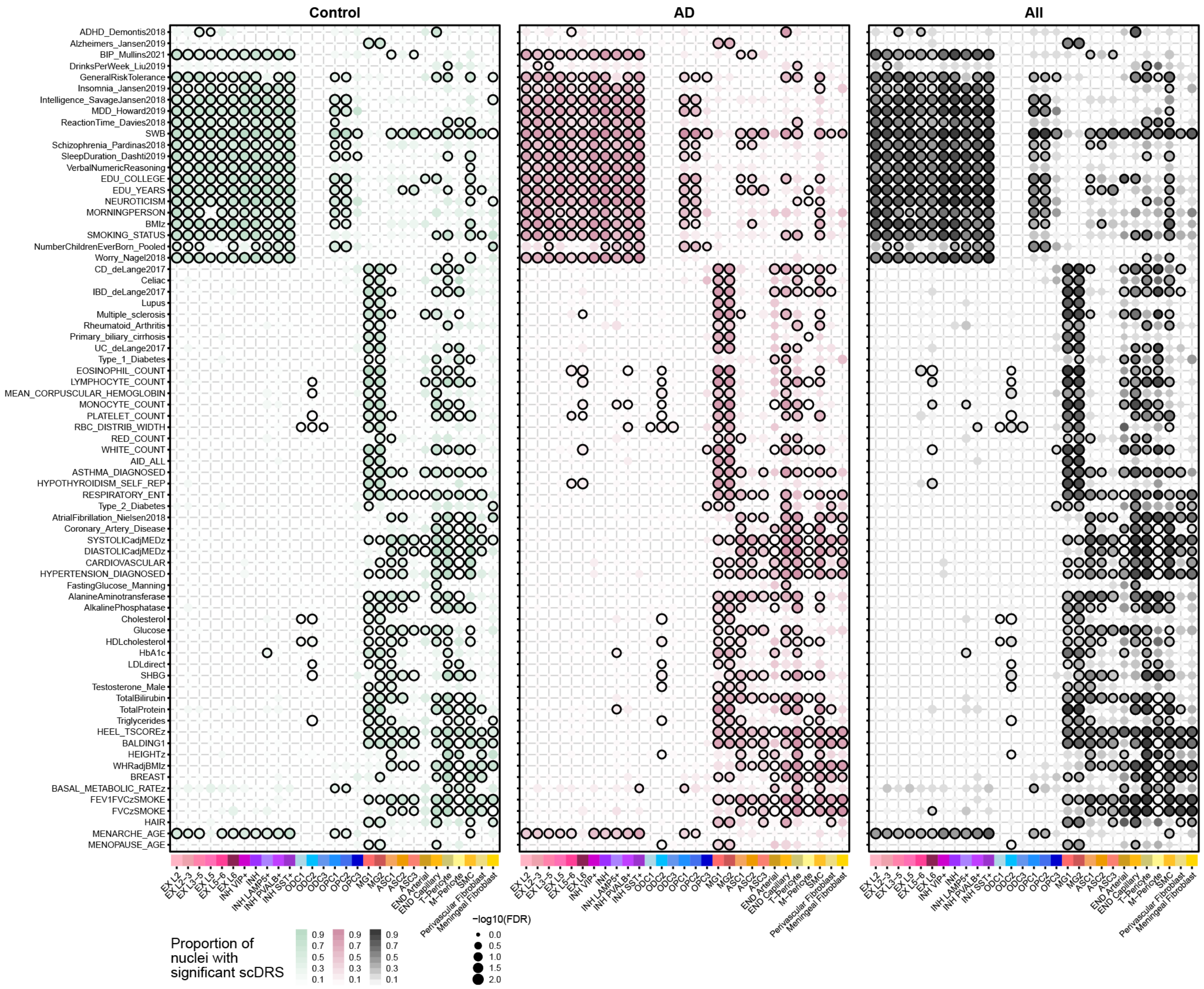

Supplementary Fig. 29 | Genetic enrichment analysis in the Morabito & Miyoshi et al [42] snRNA-seq dataset. Dot plots showing the results of genetic enrichment analysis performed in the Morabito & Miyoshi et al [42] dataset generated in this study using scDRS [44]. The scDRS Python package was run on the snRNA-seq dataset to compute single-cell disease relevance scores (sc-DRS) across a corpus of 74 traits provided by the scDRS package, resulting in cell-level disease enrichment scores and significance levels. A Monte Carlo (MC) test was used to test for group-level significance between each trait and the snRNA-seq clusters, separately for each disease group and for the entire dataset together (All, right side). Black outlines on the dots denote a significant group-level association (FDR $\leq$ 0.05).

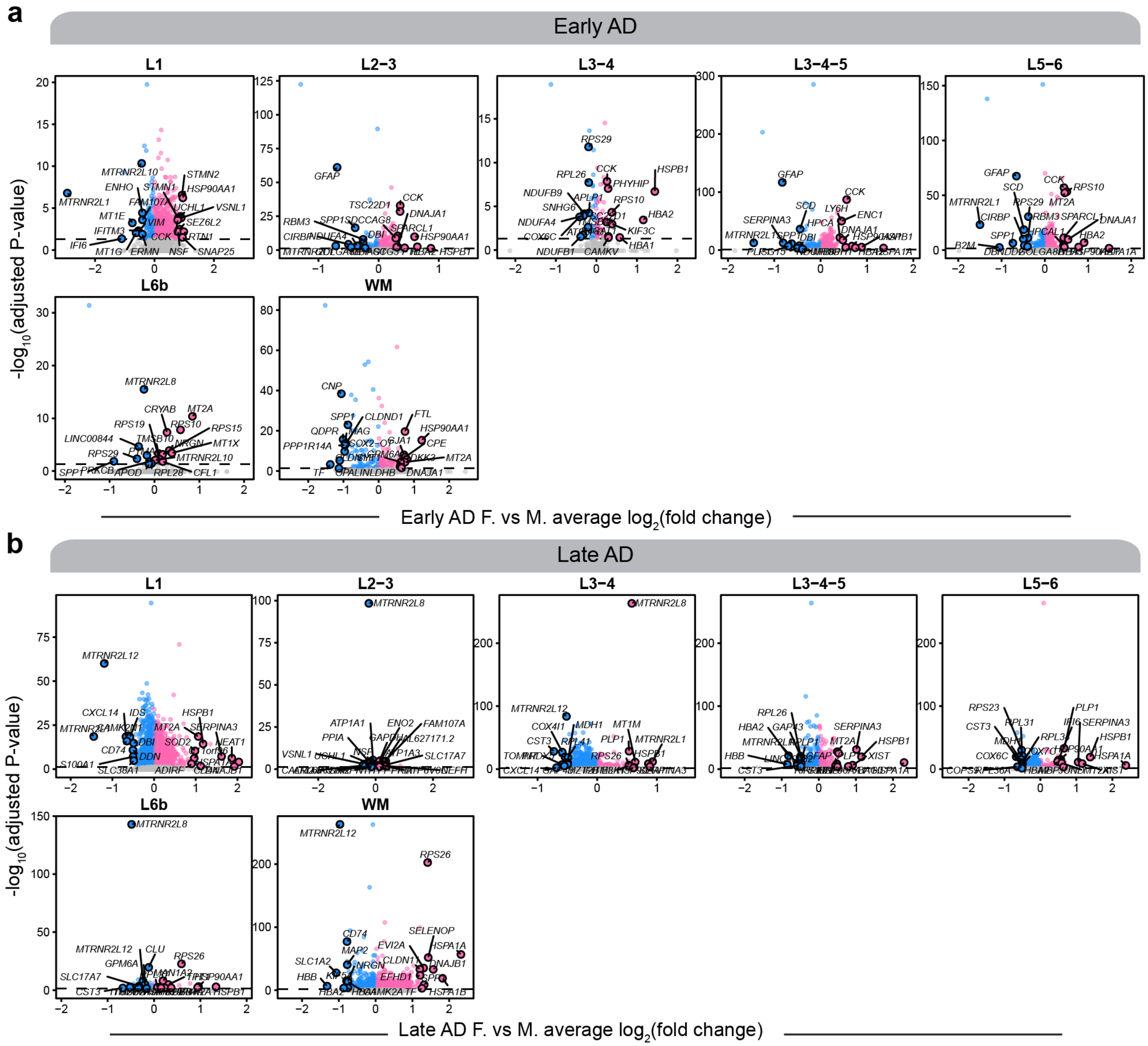

Supplementary Fig. 30 | **Sex differences within the early-stage and late-stage AD spatial transcriptomics.** Volcano plots showing the effect size and significance level from the early-stage AD (**a**) and late-stage AD (**b**) female versus male differential expression tests in each of the snRNA-seq clusters. The top 5 and bottom 5 significant genes by effect size are labeled. Statistical test: MAST[53], two-sided test.

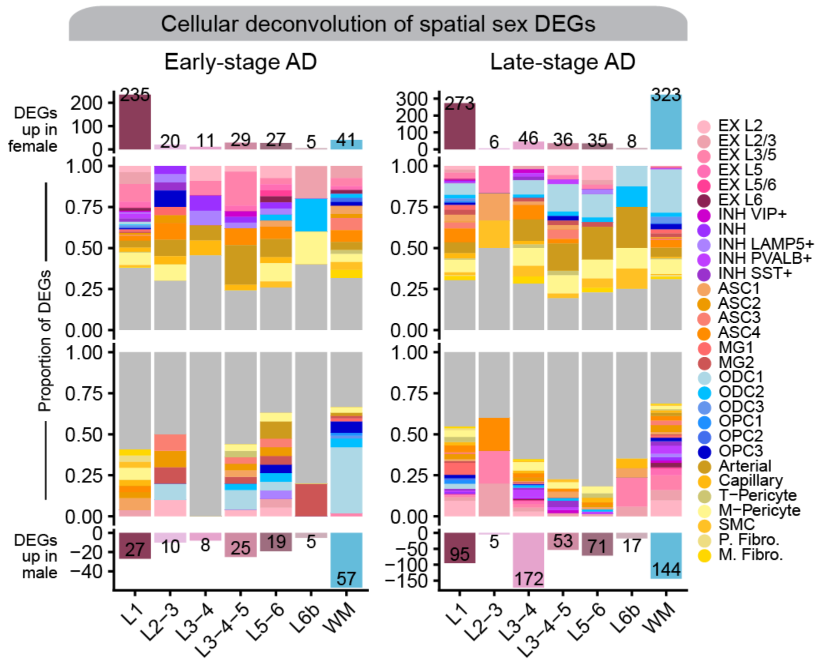

Supplementary Fig. 31 | Cellular deconvolution of sex DEGs in early-stage and late-stage AD. Deconvolution of spatial DEGs using snRNA-seq cluster marker genes. Bar charts showing the number of DEGs up or down in disease for each spatial cluster are shown on the top and bottom respectively. Proportional bar charts in the middle show the proportion of spatial DEGs that are also cluster marker genes in each of the snRNA-seq clusters. Spatial DEGs that are not in the snRNA-seq marker genes are shown in grey.

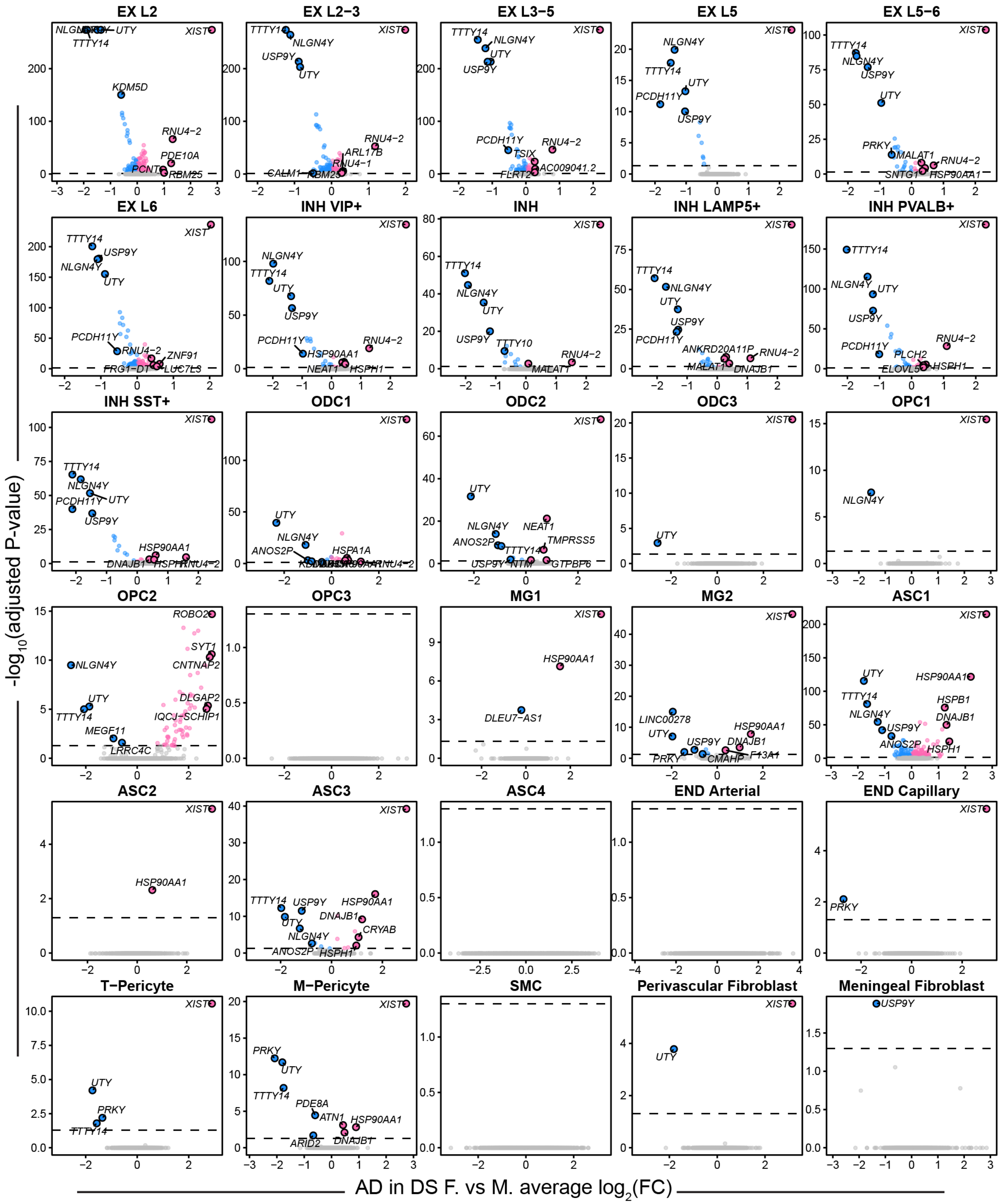

Supplementary Fig. 32 | Sex differences within the AD in DS cohort for the frontal cortex dataset snRNA-seq. Volcano plots showing the effect size and significance level from the AD in DS female versus male differential expression tests in each of the snRNA-seq clusters. The top 5 and bottom 5 significant genes by effect size are labeled. Statistical test: MAST[53], two-sided test.

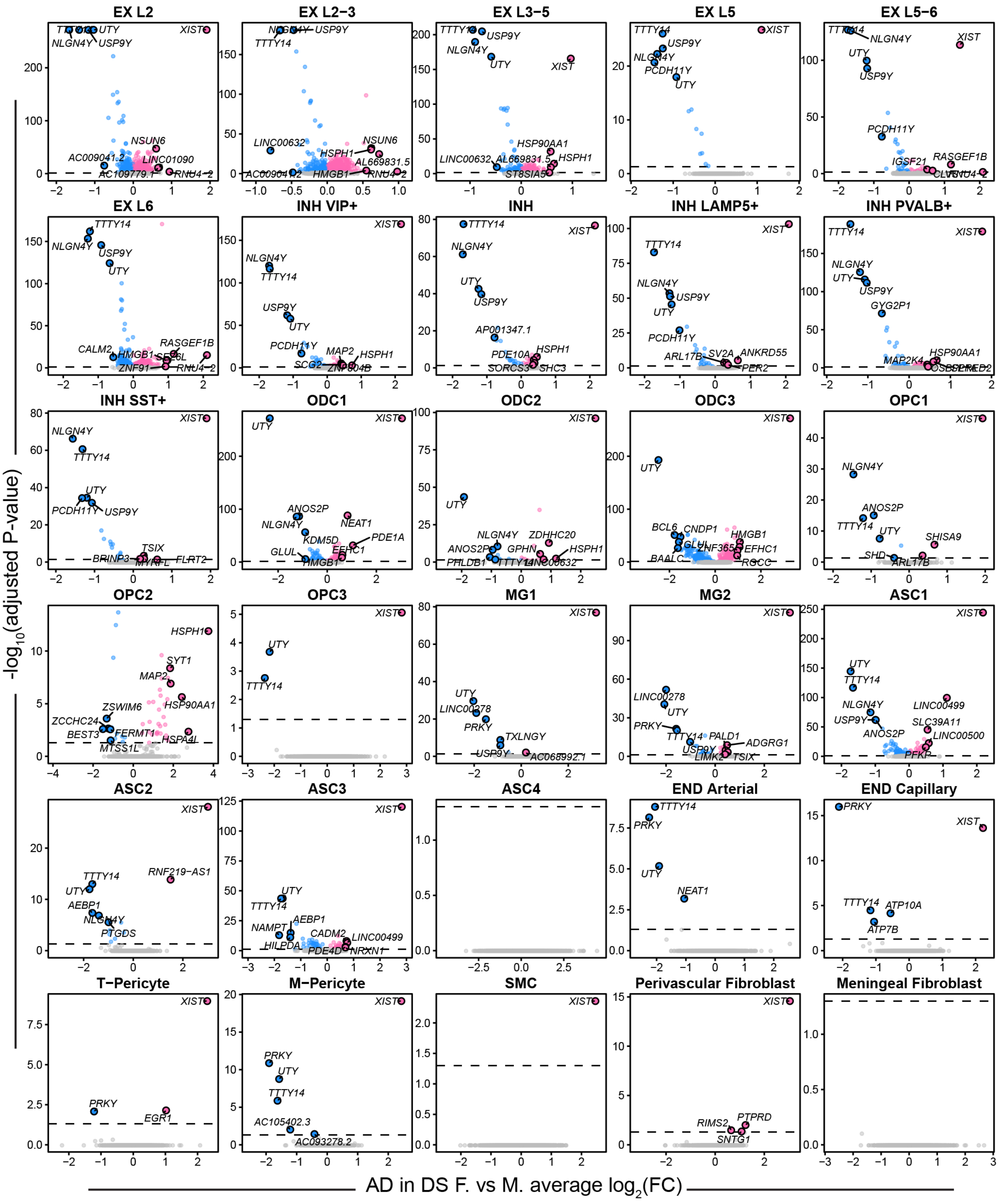

Supplementary Fig. 33 | Sex differences within the AD in DS cohort for the posterior cingulate cortex (PCC) dataset snRNA-seq. Volcano plots showing the effect size and significance level from the AD in DS female versus male differential expression tests in each of the snRNA-seq clusters. The top 5 and bottom 5 significant genes by effect size are labeled. Statistical test: MAST[53], two-sided test.

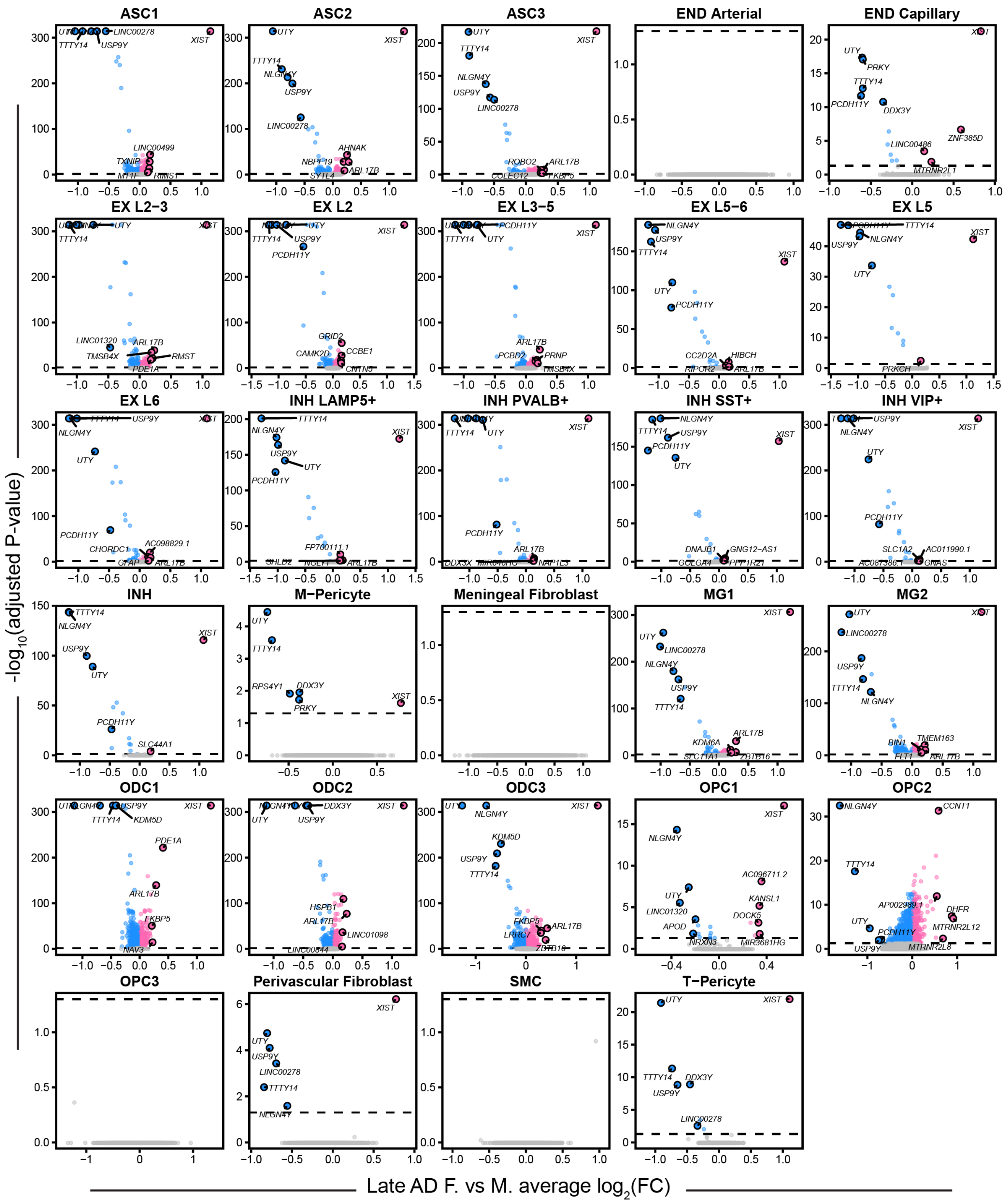

Supplementary Fig. 34 | Sex differences within late-stage AD snRNA-seq. Volcano plots showing the effect size and significance level from the late-stage AD female versus male differential expression tests in each of the snRNA-seq clusters. The top 5 and bottom 5 significant genes by effect size are labeled. Statistical test: MAST[53], two-sided test.

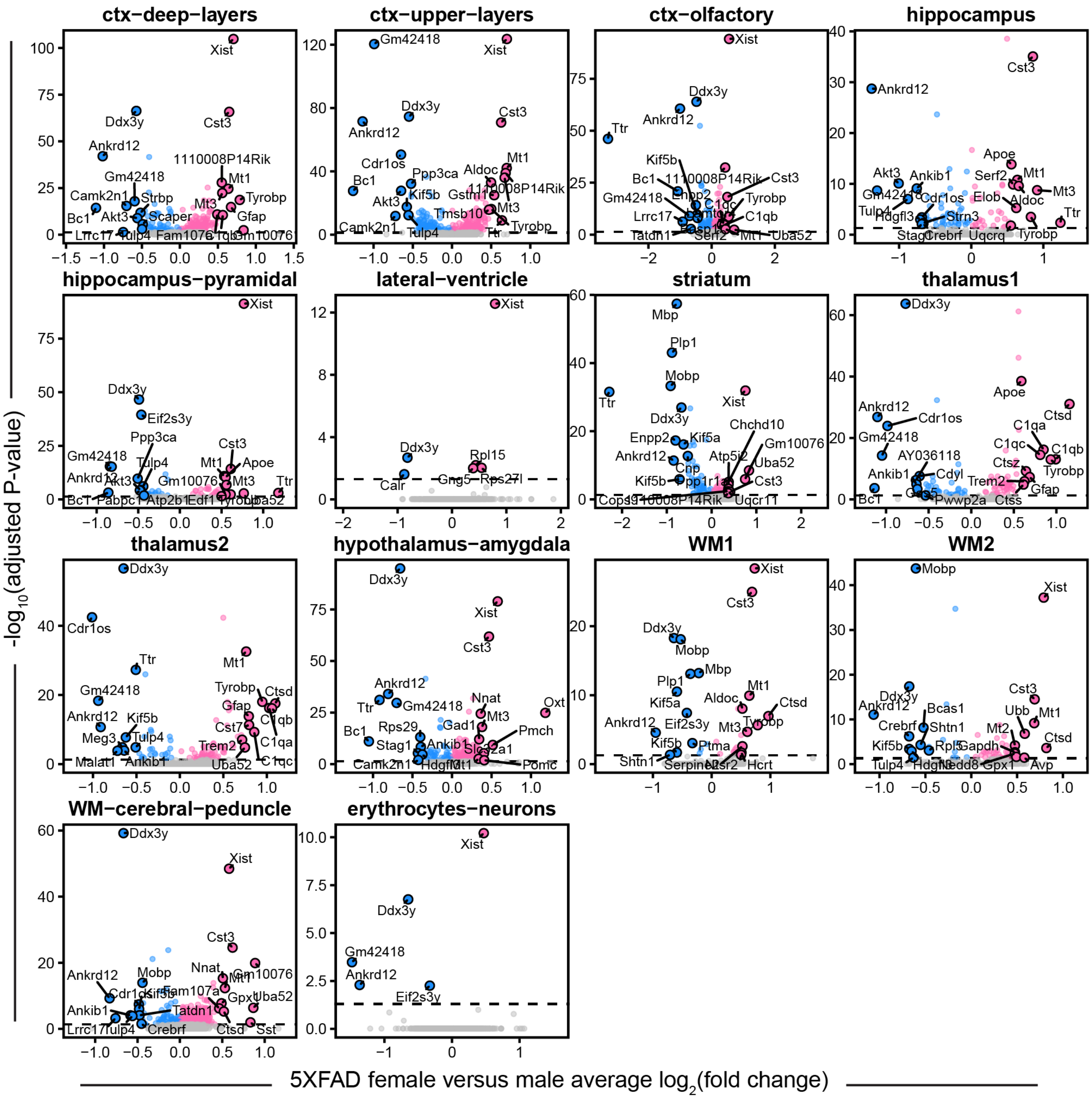

Supplementary Fig. 35 | Sex differences within 4 month 5xFAD spatial transcriptomics. Volcano plots showing the effect size and significance level from the 4 months of age 5xFAD female versus male differential expression tests in each of the snRNA-seq clusters. The top 10 and bottom 10 significant genes by effect size are labeled. Statistical test: MAST [53], two-sided test.

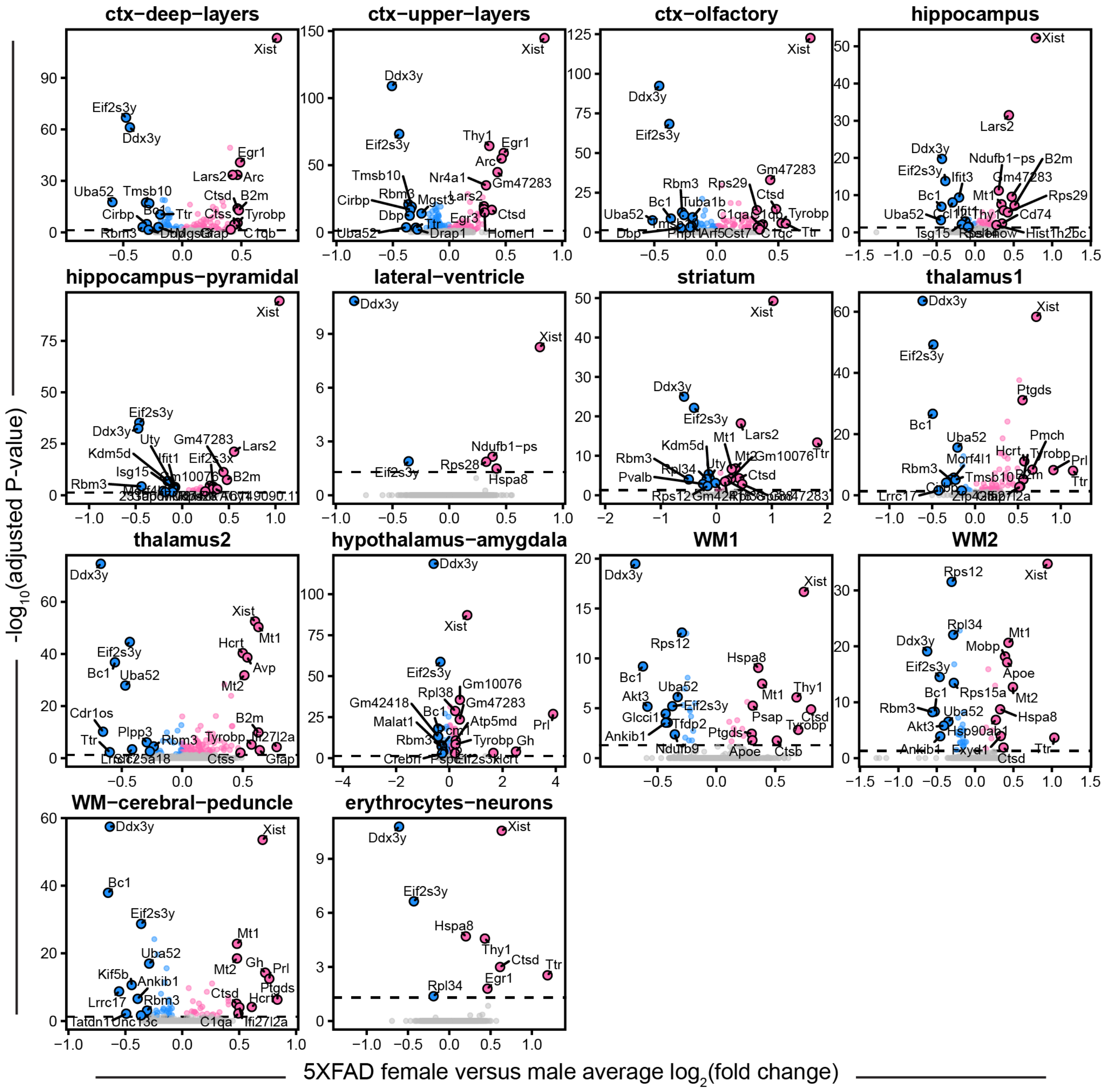

Supplementary Fig. 36 | Sex differences within 6 month 5xFAD spatial transcriptomics. Volcano plots showing the effect size and significance level from the 6 months of age 5xFAD female versus male differential expression tests in each of the snRNA-seq clusters. The top 10 and bottom 10 significant genes by effect size are labeled. Statistical test: MAST[53], two-sided test.

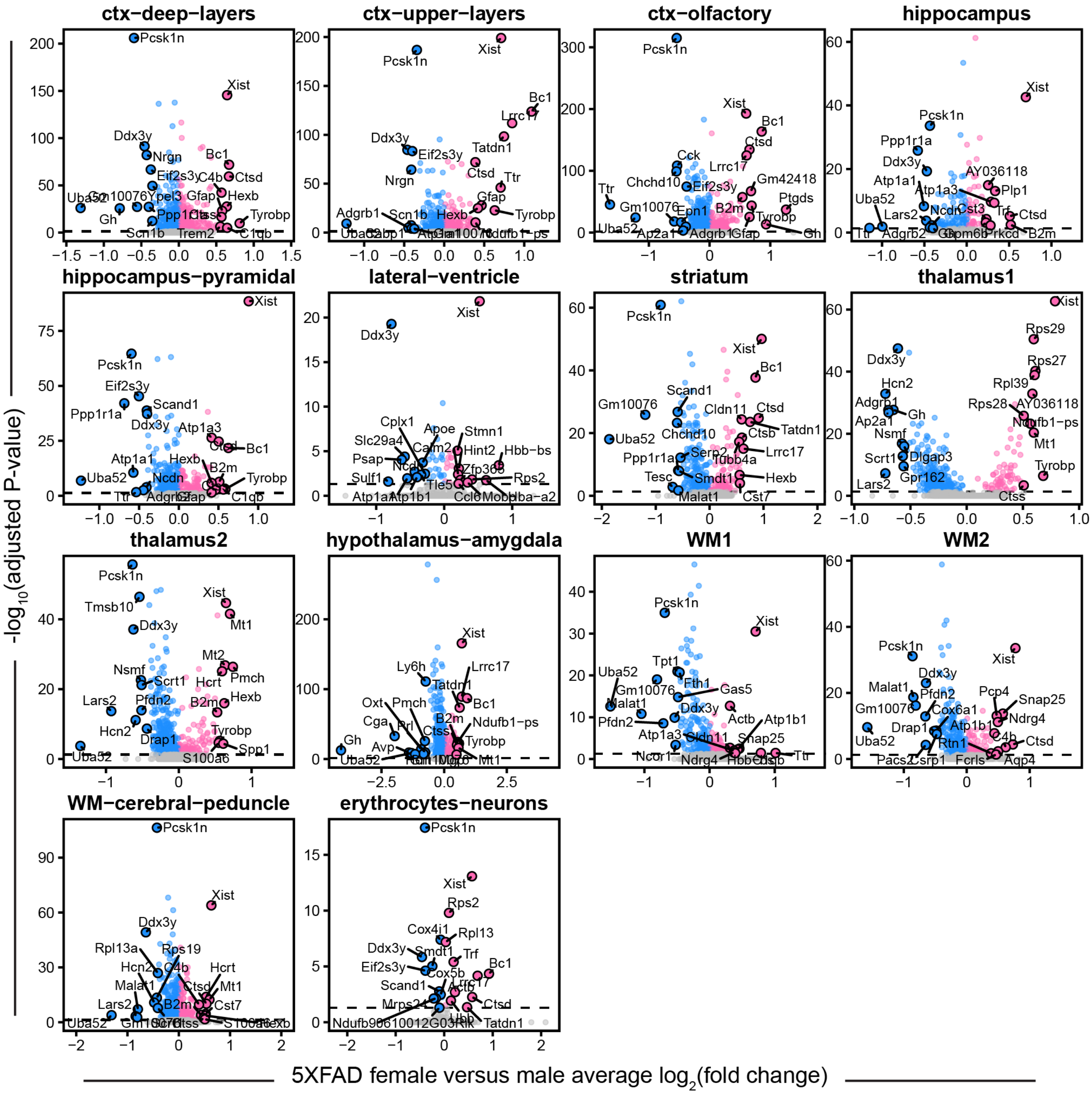

Supplementary Fig. 37 | Sex differences within 8 month 5xFAD spatial transcriptomics. Volcano plots showing the effect size and significance level from the 8 months of age 5xFAD female versus male differential expression tests in each of the snRNA-seq clusters. The top 10 and bottom 10 significant genes by effect size are labeled. Statistical test: MAST [53], two-sided test.

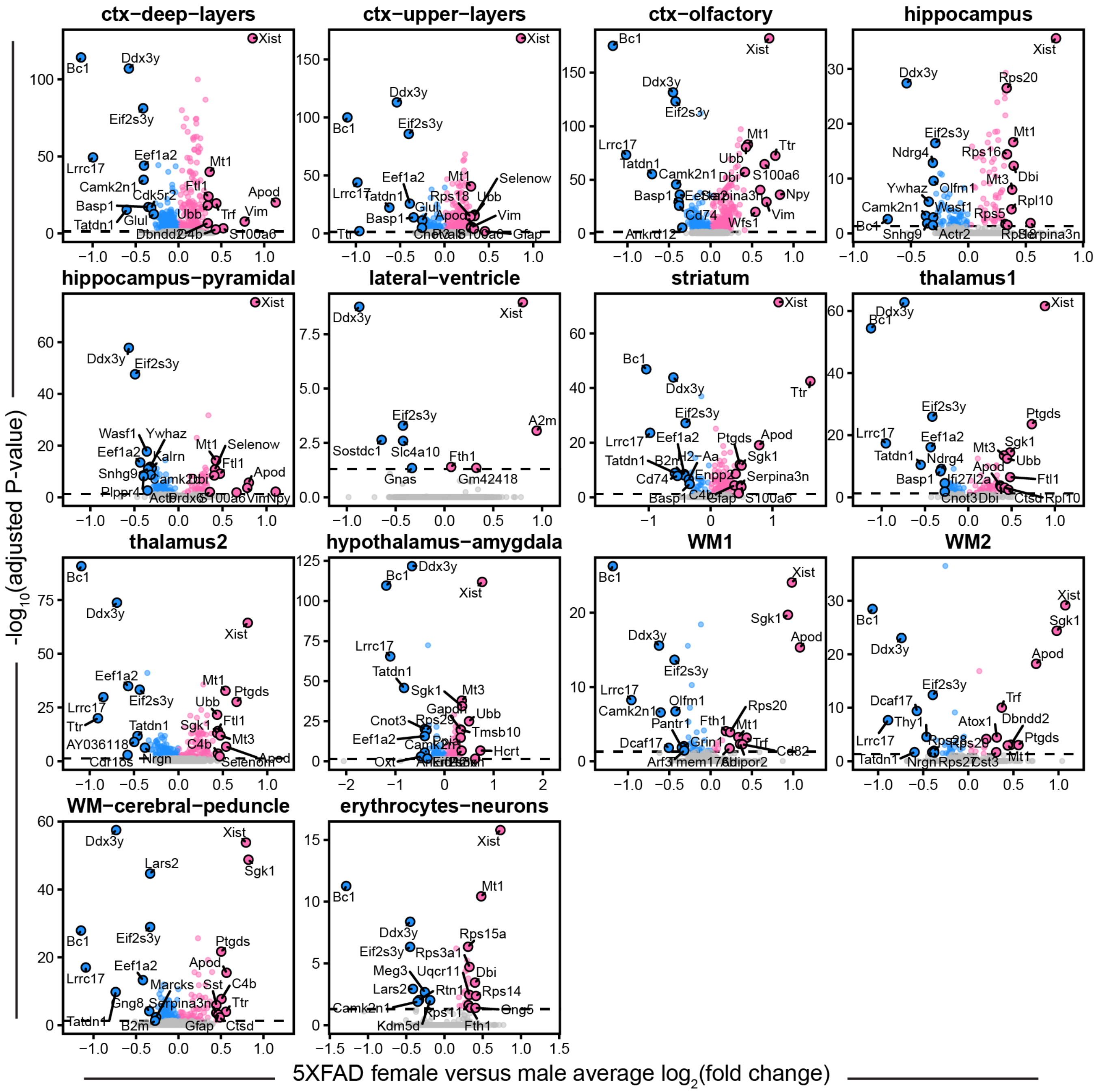

Supplementary Fig. 38 | Sex differences within 12 month 5xFAD spatial transcriptomics. Volcano plots showing the effect size and significance level from the 12 months of age 5xFAD female versus male differential expression tests in each of the snRNA-seq clusters. The top 10 and bottom 10 significant genes by effect size are labeled. Statistical test: MAST [53], two-sided test.

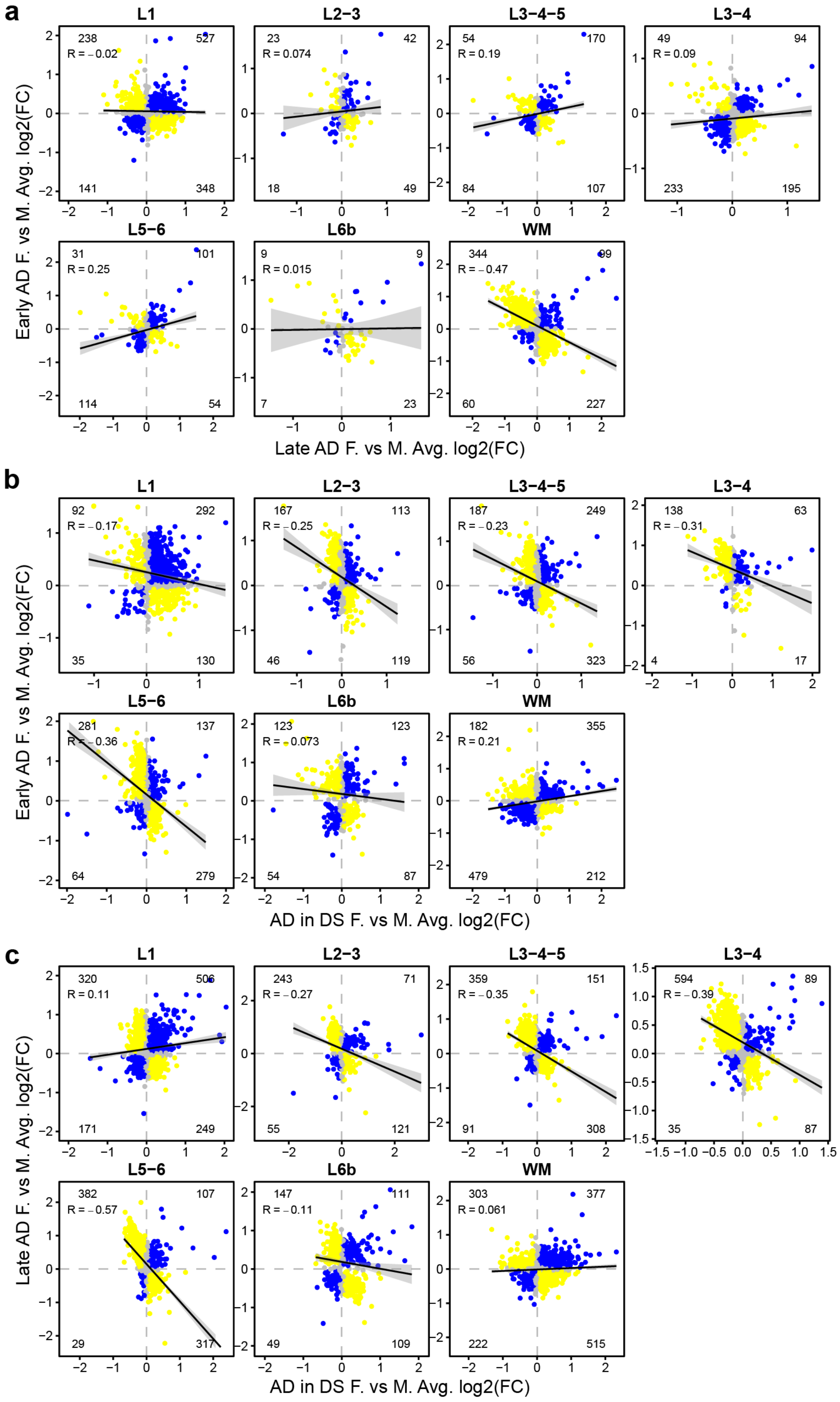

Supplementary Fig. 39 | Comparison of female vs. male spatial transcriptomics differential expression effect sizes across disease conditions. Comparison of female vs. male differential expression effect sizes from early-stage AD and late-stage AD (**a**); early-stage AD and AD in DS (**b**); late-stage AD and AD in DS (**c**). Genes that were statistically significant (adjusted p-value < 0.05) in either comparison were included in this analysis. Genes are colored blue if the direction is consistent, yellow if inconsistent, and grey if the absolute effect sizes were smaller than 0.05. Black line represents a linear regression with a 95% confidence interval around the mean shown in grey. Pearson correlation coefficients are shown in the upper left corner of each panel. Numbers of inconsistent and consistent genes in each quadrant are reported in the corners of each plot.

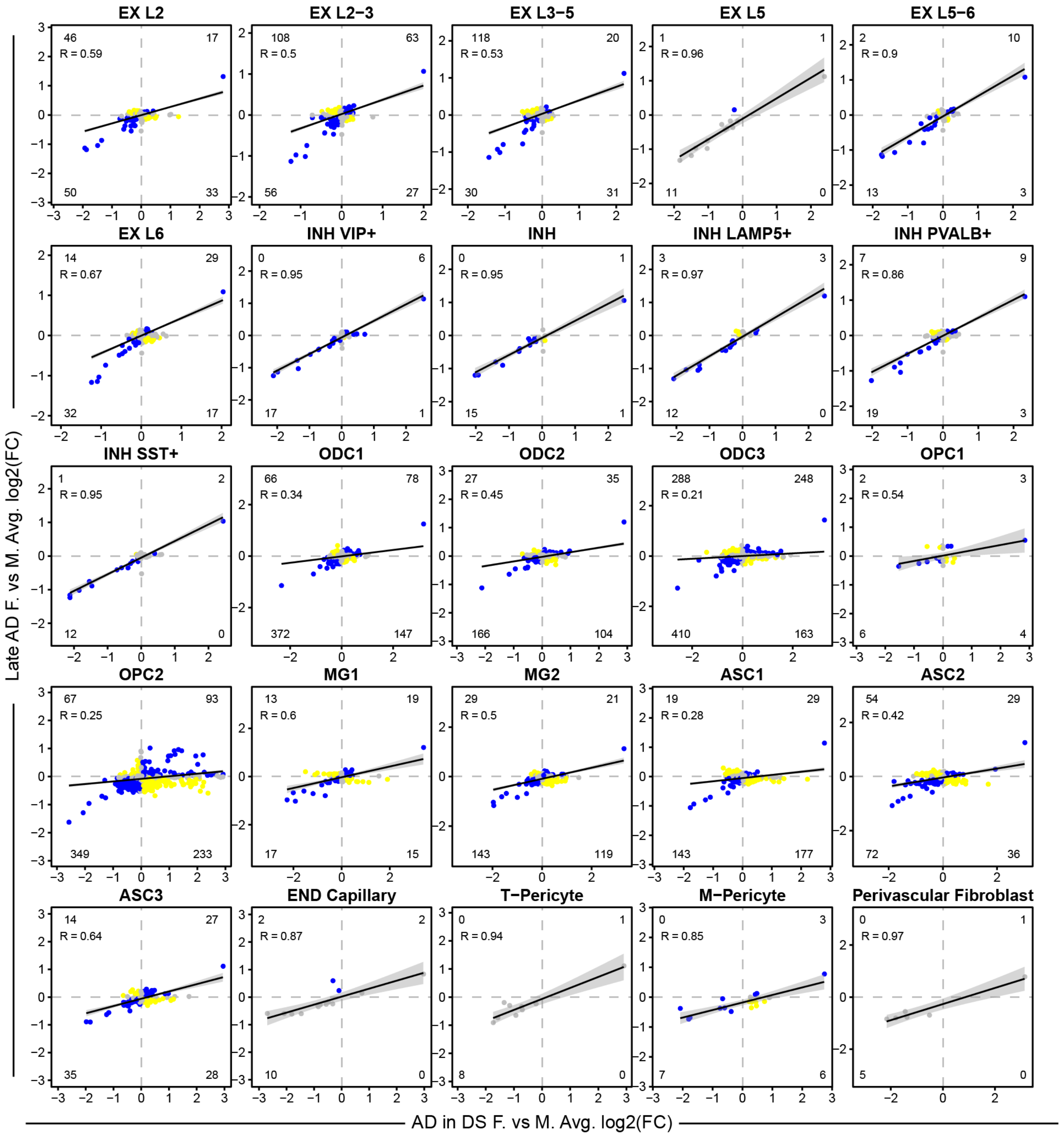

Supplementary Fig. 40 | Comparison of female vs. male differential expression effect sizes between AD in DS and late-stage AD snRNA-seq. Comparison of female vs. male differential expression effect sizes in AD in DS and late-stage AD for each snRNA-seq cluster. Genes that were statistically significant (adjusted p-value < 0.05) in either comparison were included in this analysis. Genes are colored blue if the direction is consistent, yellow if inconsistent, and grey if the absolute effect sizes were smaller than 0.05. Black line represents a linear regression with a 95% confidence interval around the mean shown in grey. Pearson correlation coefficients are shown in the upper left corner of each panel. Numbers of inconsistent and consistent genes in each quadrant are reported in the corners of each plot.

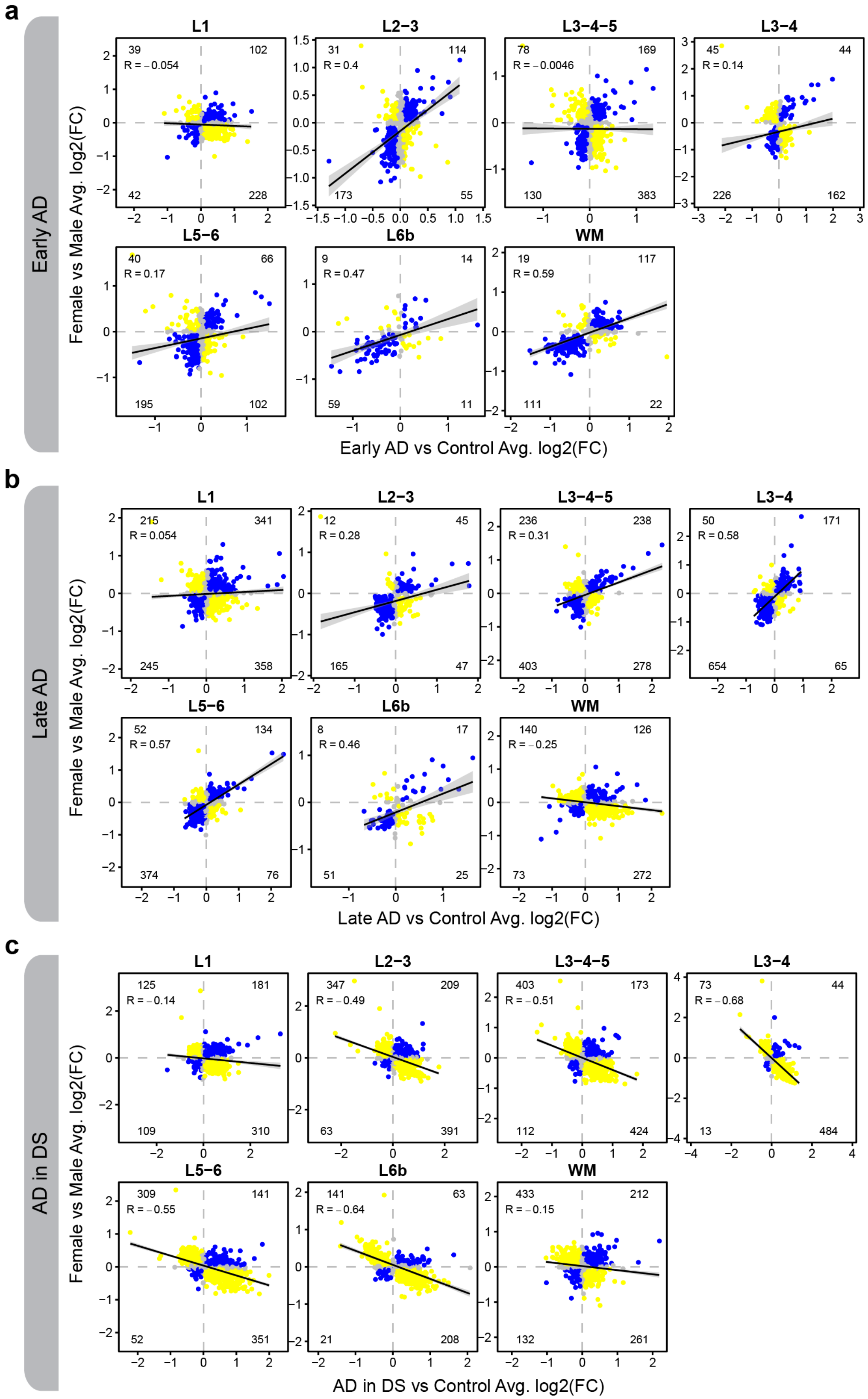

Supplementary Fig. 41 | Comparison of female vs. male and disease vs. control differential expression effect sizes across disease conditions in spatial transcriptomics. Comparison of female vs. male and disease vs. control differential expression effect sizes from early-stage AD and late-stage AD (**a**); early-stage AD and AD in DS (**b**); late-stage AD and AD in DS (**c**). Genes that were statistically significant (adjusted p-value < 0.05) in either comparison were included in this analysis. Genes are colored blue if the direction is consistent, yellow if inconsistent, and grey if the absolute effect sizes were smaller than 0.05. Black line represents a linear regression with a 95% confidence interval around the mean shown in grey. Pearson correlation coefficients are shown in the upper left corner of each panel. Numbers of inconsistent and consistent genes in each quadrant are reported in the corners of each plot.

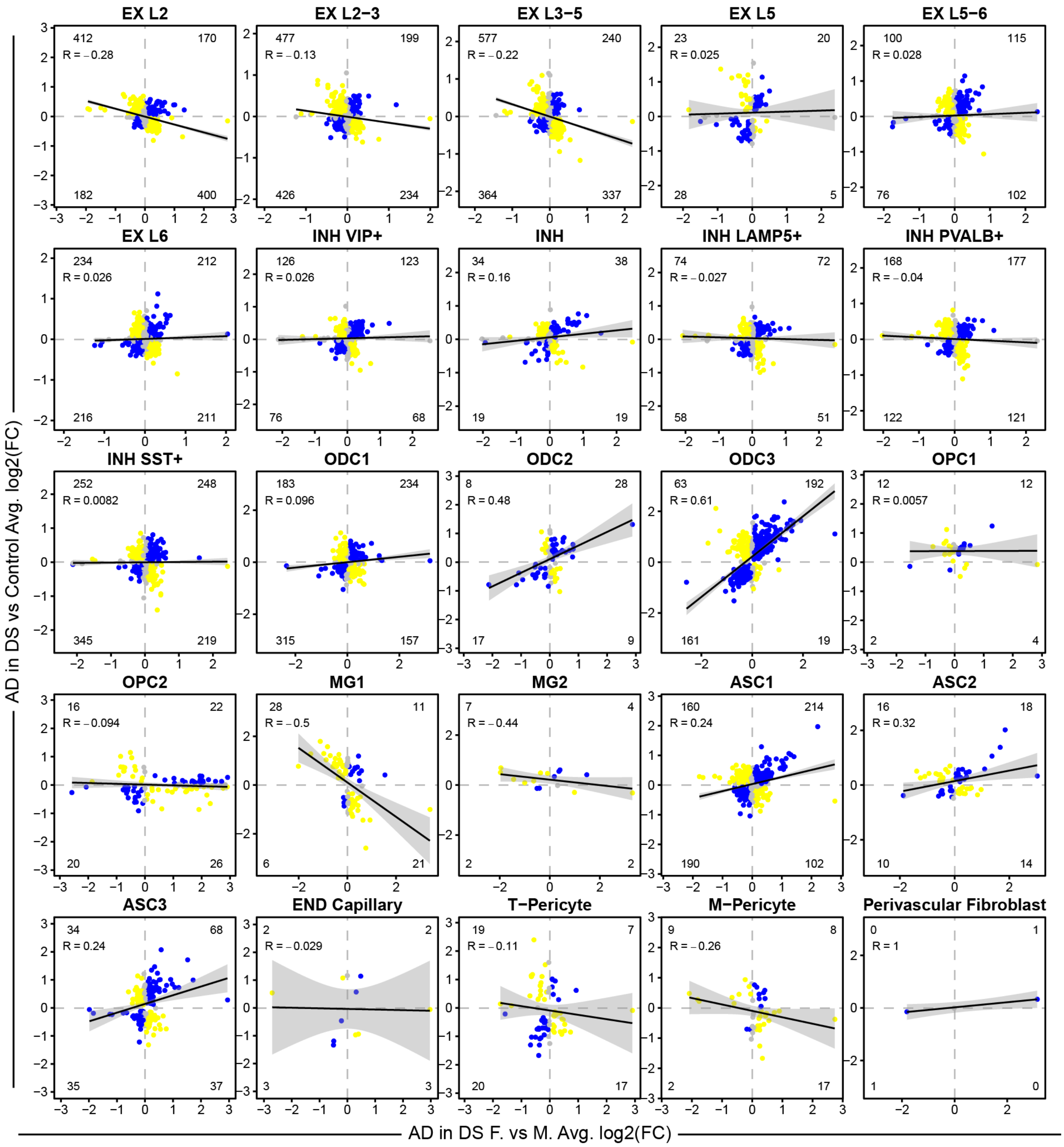

Supplementary Fig. 42 | Comparison of female vs. male and AD in DS vs. control differential expression effect sizes across disease conditions in frontal cortex snRNA-seq. Comparison of female vs. male and AD in DS vs. control (frontal cortex) differential expression effect sizes. Genes that were statistically significant (adjusted p-value < 0.05) in either comparison were included in this analysis. Genes are colored blue if the direction is consistent, yellow if inconsistent, and grey if the absolute effect sizes were smaller than 0.05. Black line represents a linear regression with a 95% confidence interval around the mean shown in grey. Pearson correlation coefficients are shown in the upper left corner of each panel. Numbers of inconsistent and consistent genes in each quadrant are reported in the corners of each plot.

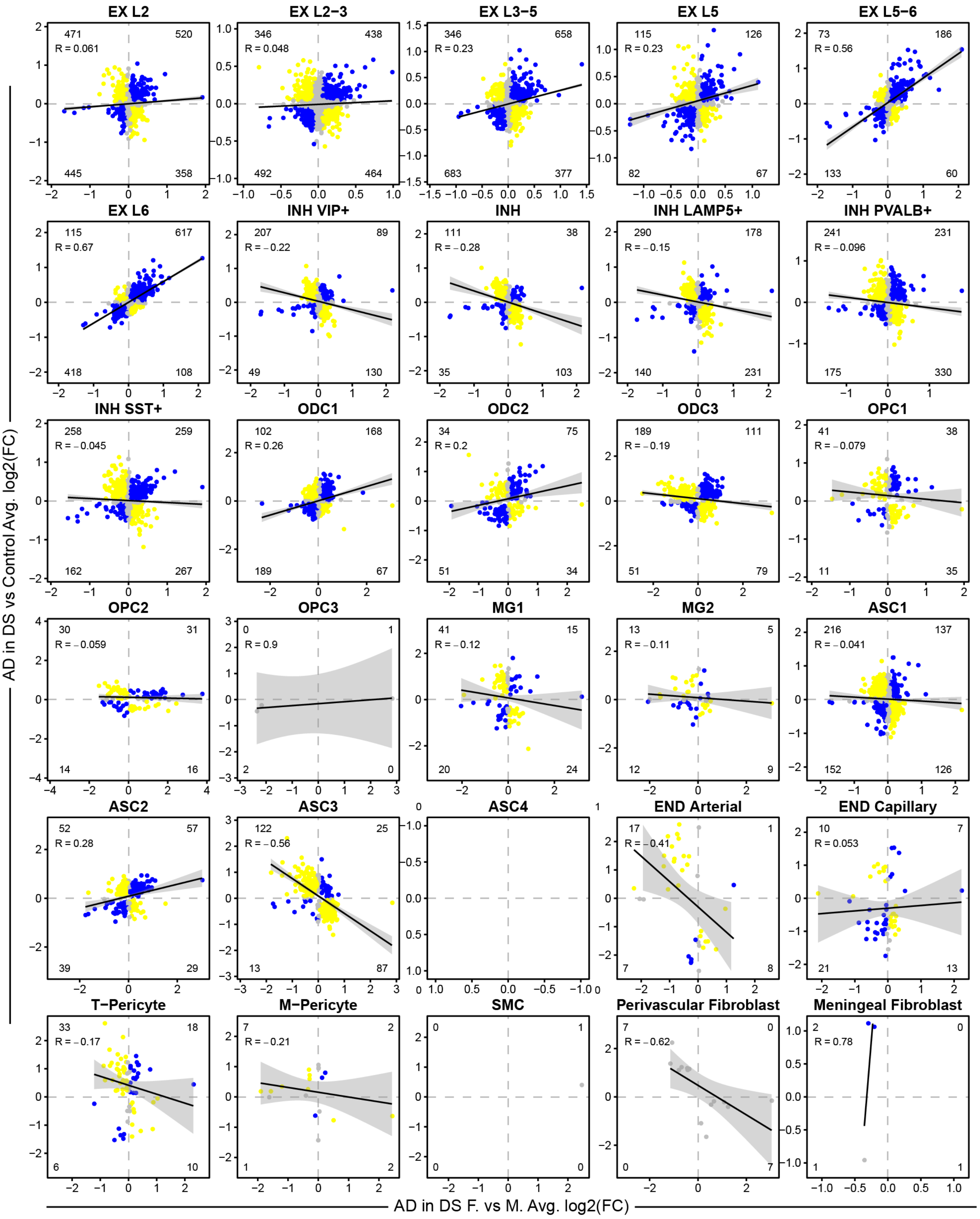

Supplementary Fig. 43 | Comparison of female vs. male and AD in DS vs. control differential expression effect sizes across disease conditions in posterior cingulate cortex snRNA-seq. Comparison of female vs. male and AD in DS vs. control (posterior cingluate cortex) differential expression effect sizes. Genes that were statistically significant (adjusted p-value < 0.05) in either comparison were included in this analysis. Genes are colored blue if the direction is consistent, yellow if inconsistent, and grey if the absolute effect sizes were smaller than 0.05. Black line represents a linear regression with a 95% confidence interval around the mean shown in grey. Pearson correlation coefficients are shown in the upper left corner of each panel. Numbers of inconsistent and consistent genes in each quadrant are reported in the corners of each plot.

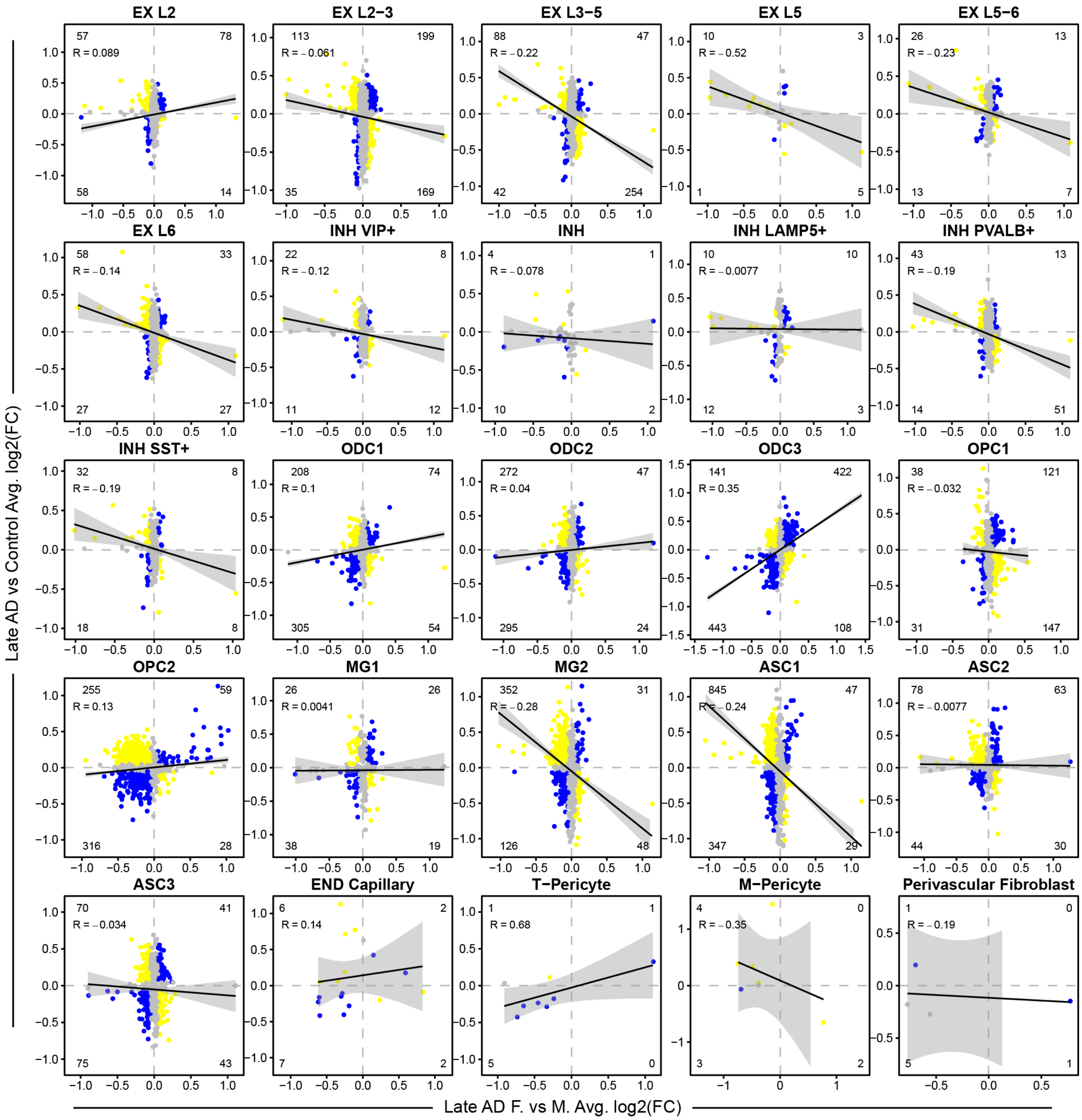

Supplementary Fig. 44 | Comparison of female vs. male and AD vs. control differential expression effect sizes across disease conditions in late-stage AD snRNA-seq. Comparison of female vs. male and late-stage AD vs. control differential expression effect sizes. Genes that were statistically significant (adjusted p-value < 0.05) in either comparison were included in this analysis. Genes are colored blue if the direction is consistent, yellow if inconsistent, and grey if the absolute effect sizes were smaller than 0.05. Black line represents a linear regression with a 95% confidence interval around the mean shown in grey. Pearson correlation coefficients are shown in the upper left corner of each panel. Numbers of inconsistent and consistent genes in each quadrant are reported in the corners of each plot.

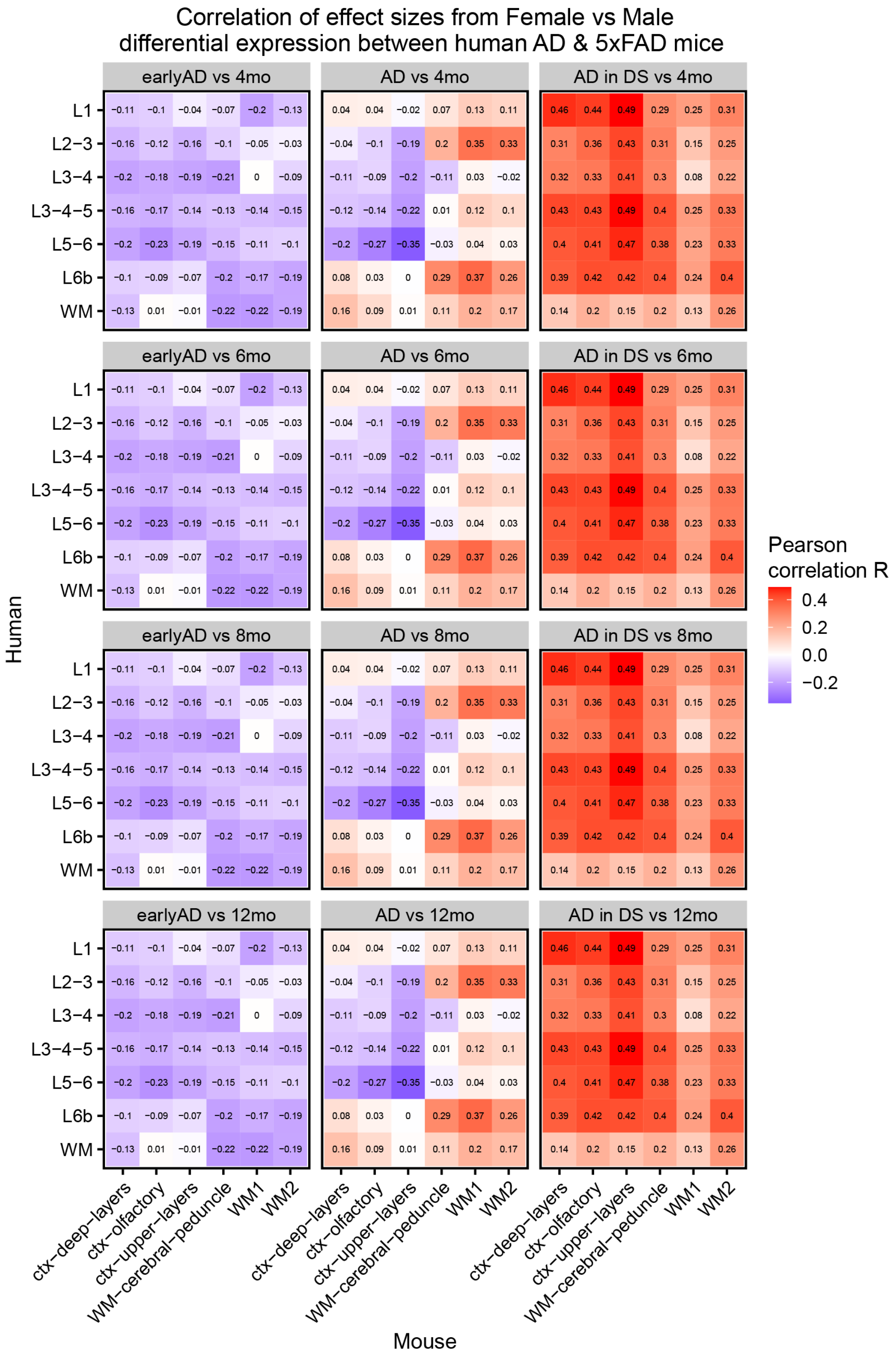

Supplementary Fig. 45 | Comparison of female vs. male differential expression effect sizes between human and mouse spatial transcriptomics. Comparison of female vs. male differential expression effect sizes between different age groups for 5xFAD mice and different disease groups for humans for spatial transcriptomic clusters. Pearson correlation coefficients were computed to compare the differential expression effect sizes between groups.

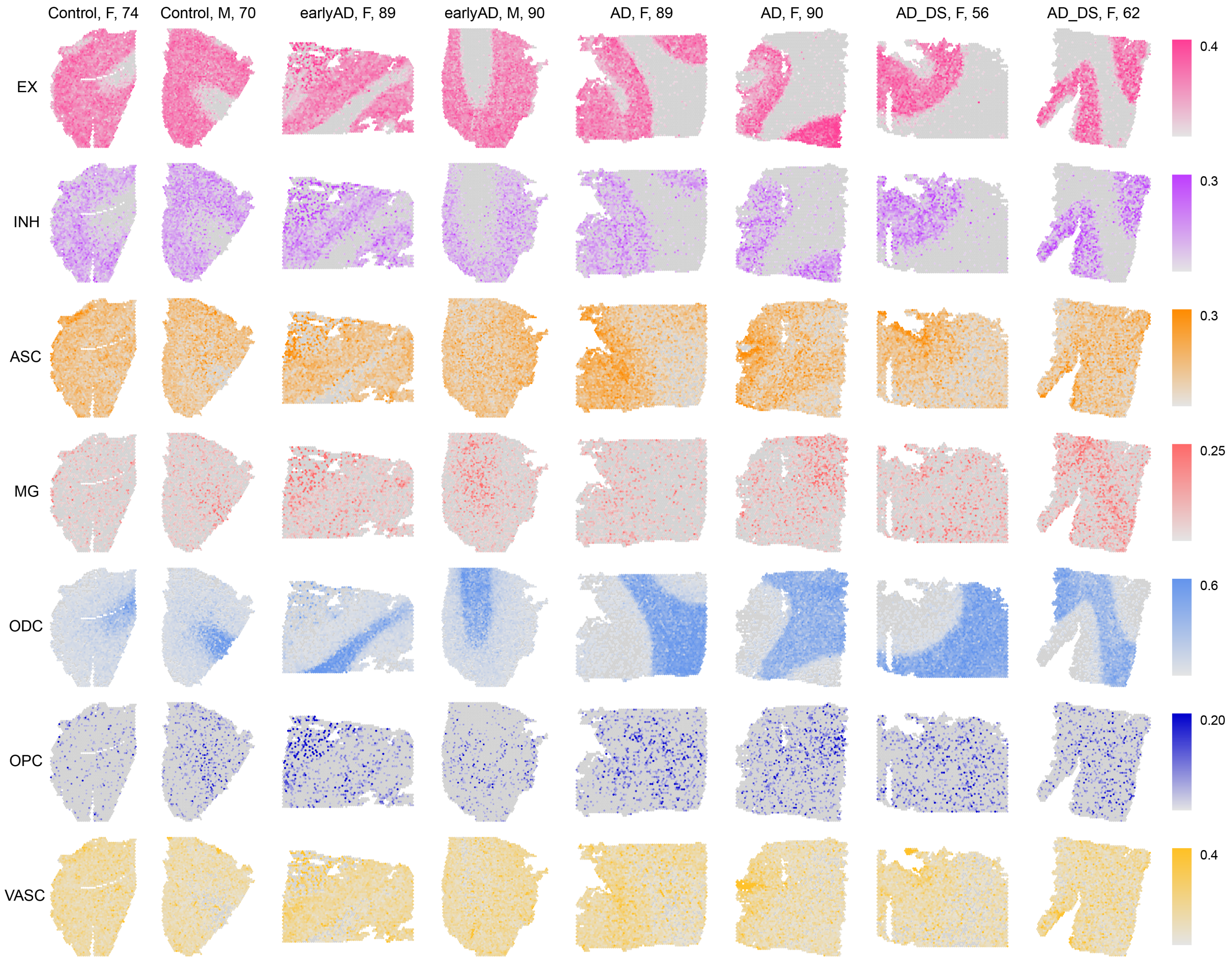

Supplementary Fig. 46 | Cell-type deconvolution results in the human ST dataset. Spatial feature plots in eight representative samples from the human ST dataset showing the inferred proportion of different major cell types based on our deconvolution analysis conducted with SPOTLight[55]..

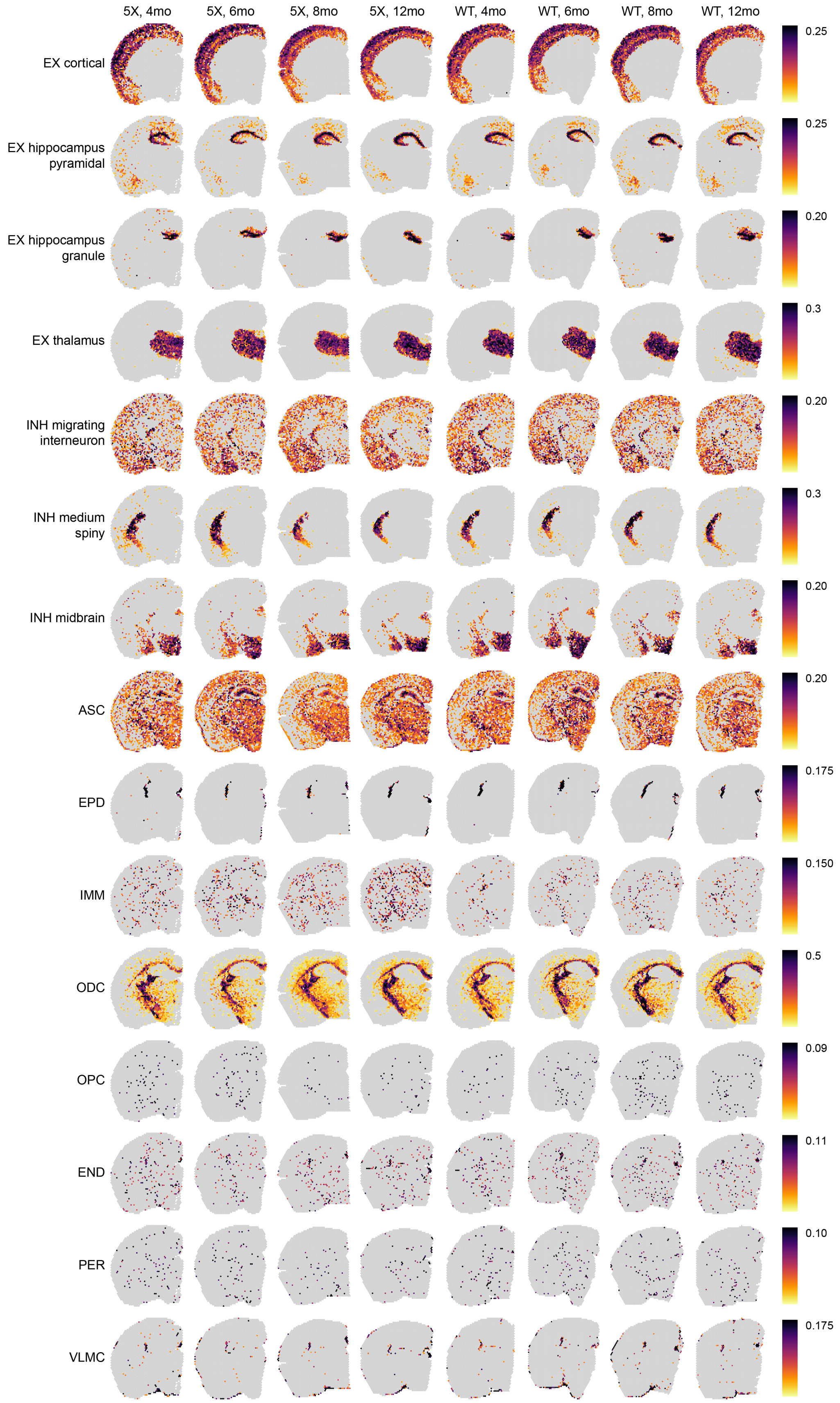

Supplementary Fig. 47 | Cell-type deconvolution results in the mouse ST dataset. Spatial feature plots in eight representative samples from the mouse ST dataset showing the inferred proportion of different major cell types based on our deconvolution analysis conducted with SPOTLight[55].

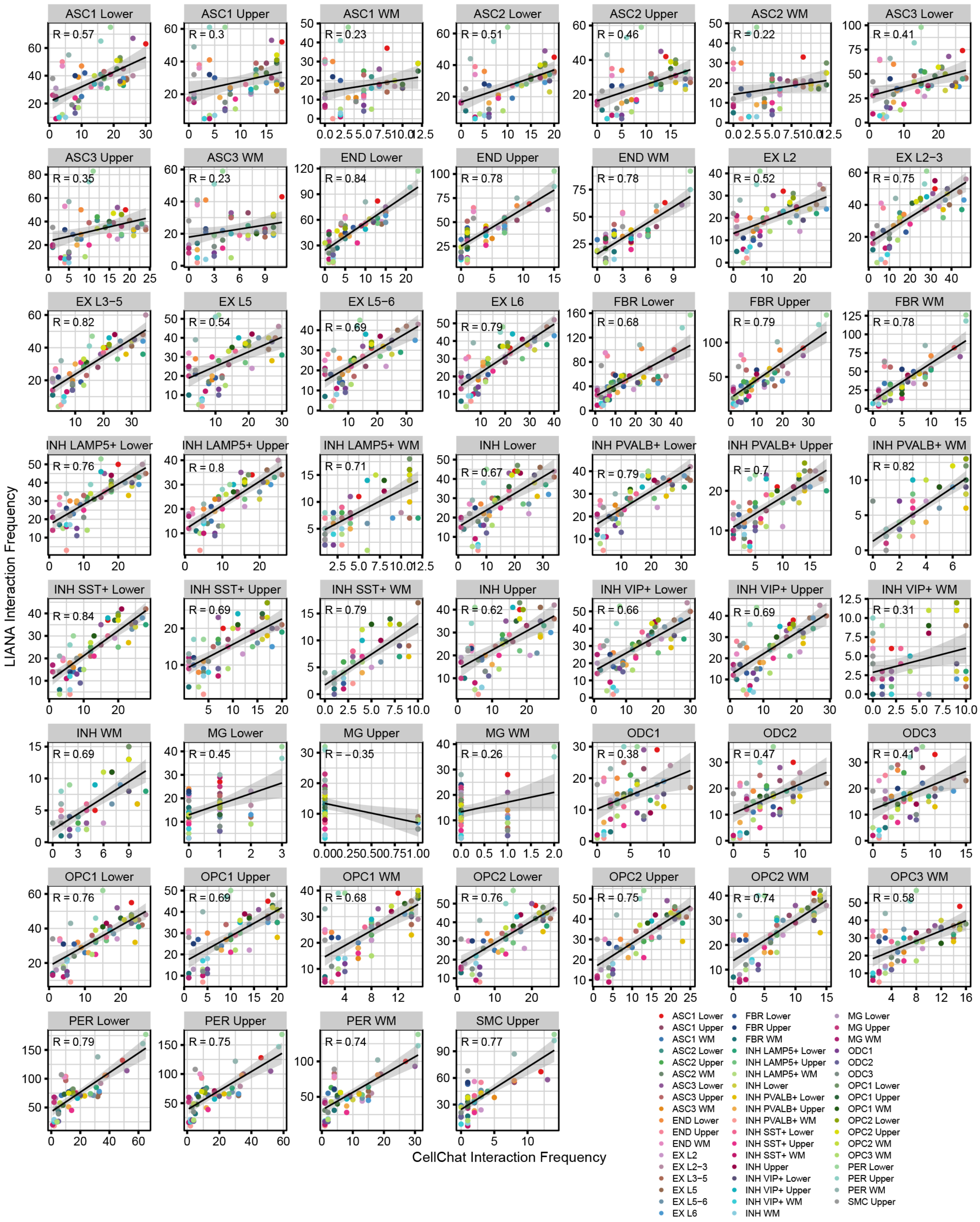

Supplementary Fig. 48 | **Comparison of predicting cell-cell signaling interactions between CellChat**[17] **and LIANA**[18] **in control snRNA-seq.** Scatter plots show the cell-cell communication interaction frequencies in CellChat[17] and LIANA[18]. Panels are grouped for each cell cluster as the signal sender, where the interaction frequencies represent the number of links to all other cell clusters. Black line represents a linear regression with a 95% confidence interval around the mean shown in grey. Pearson correlation coefficients are shown in the upper left corner of each panel.

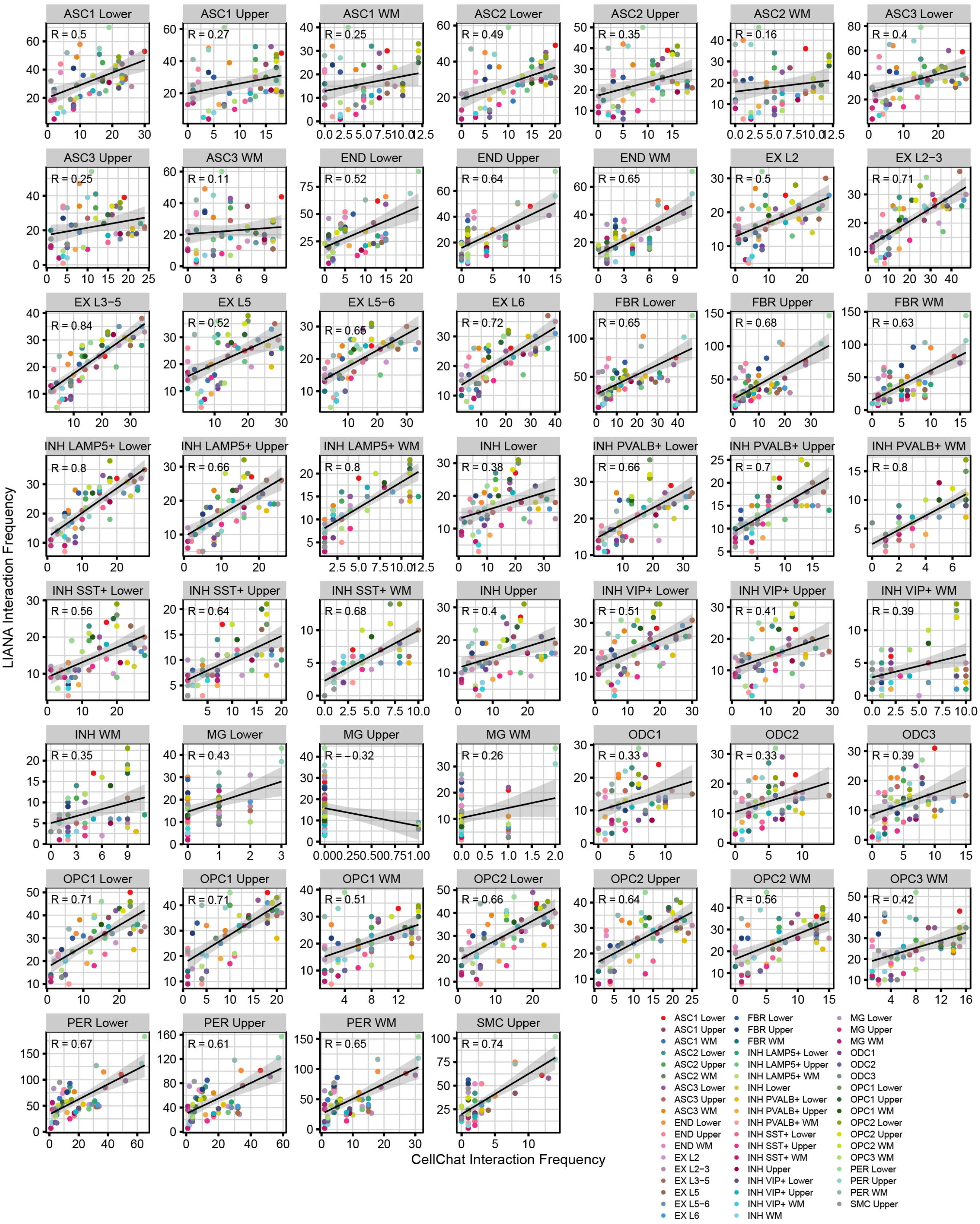

Supplementary Fig. 49 | Comparison of predicting cell-cell signaling interactions between CellChat [17] and LIANA [18] in AD in DS snRNA-seq. Scatter plots show the cell-cell communication interaction frequencies in CellChat [17] and LIANA [18]. Panels are grouped for each cell cluster as the signal sender, where the interaction frequencies represent the number of links to all other cell clusters. Black line represents a linear regression with a 95% confidence interval around the mean shown in grey. Pearson correlation coefficients are shown in the upper left corner of each panel.

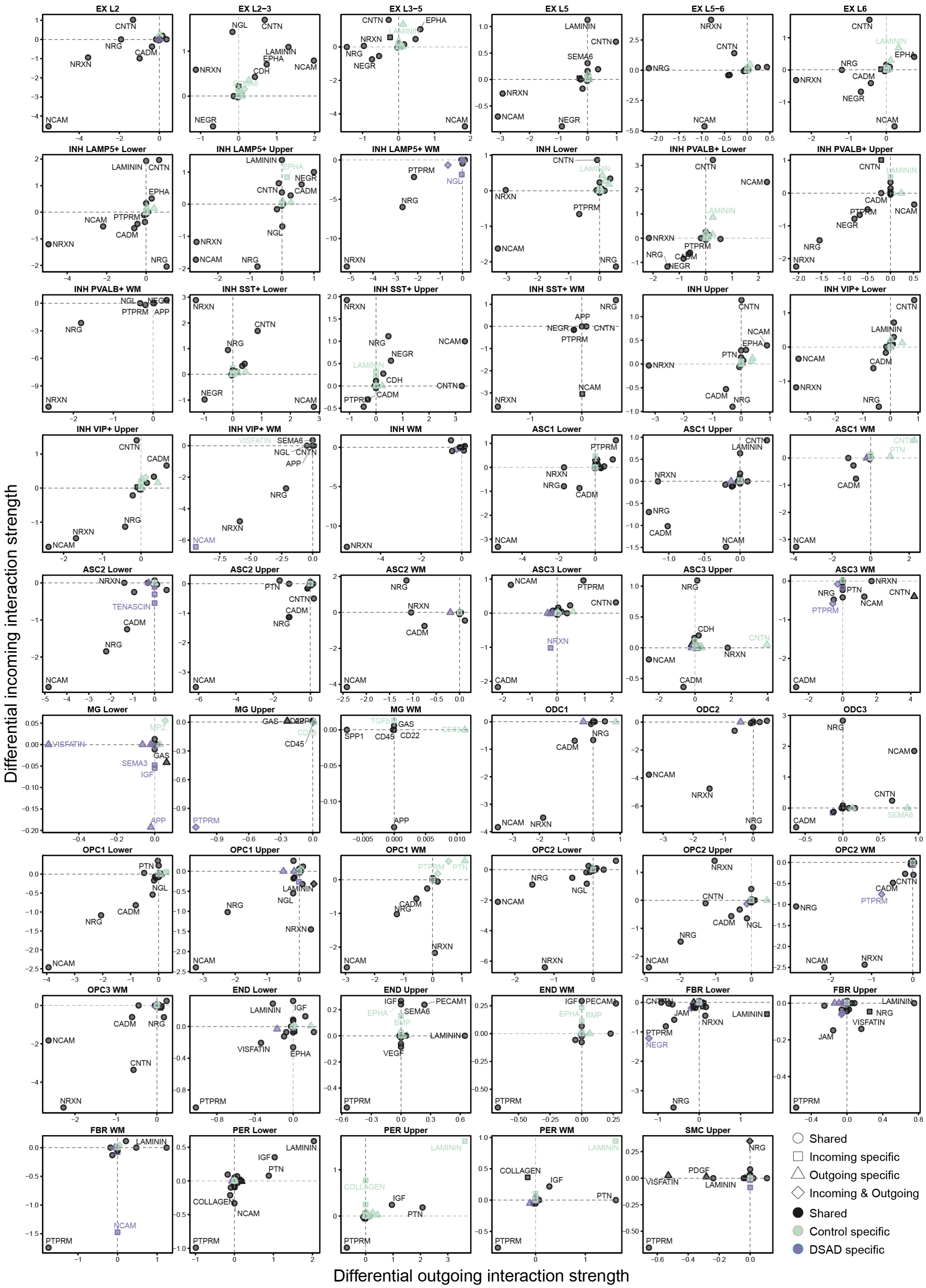

Supplementary Fig. 50 | Differential cell-cell signaling between AD in DS and control. Scatter plots showing the differential outgoing interaction strength versus the differential incoming interaction strength from the differential cell-cell signaling network analysis between AD in DS cases versus cognitively normal controls. Pathways were shown in each cluster where there was a statistically significant (p-value < 0.05) difference between AD in DS and control based on a permutation test[17].

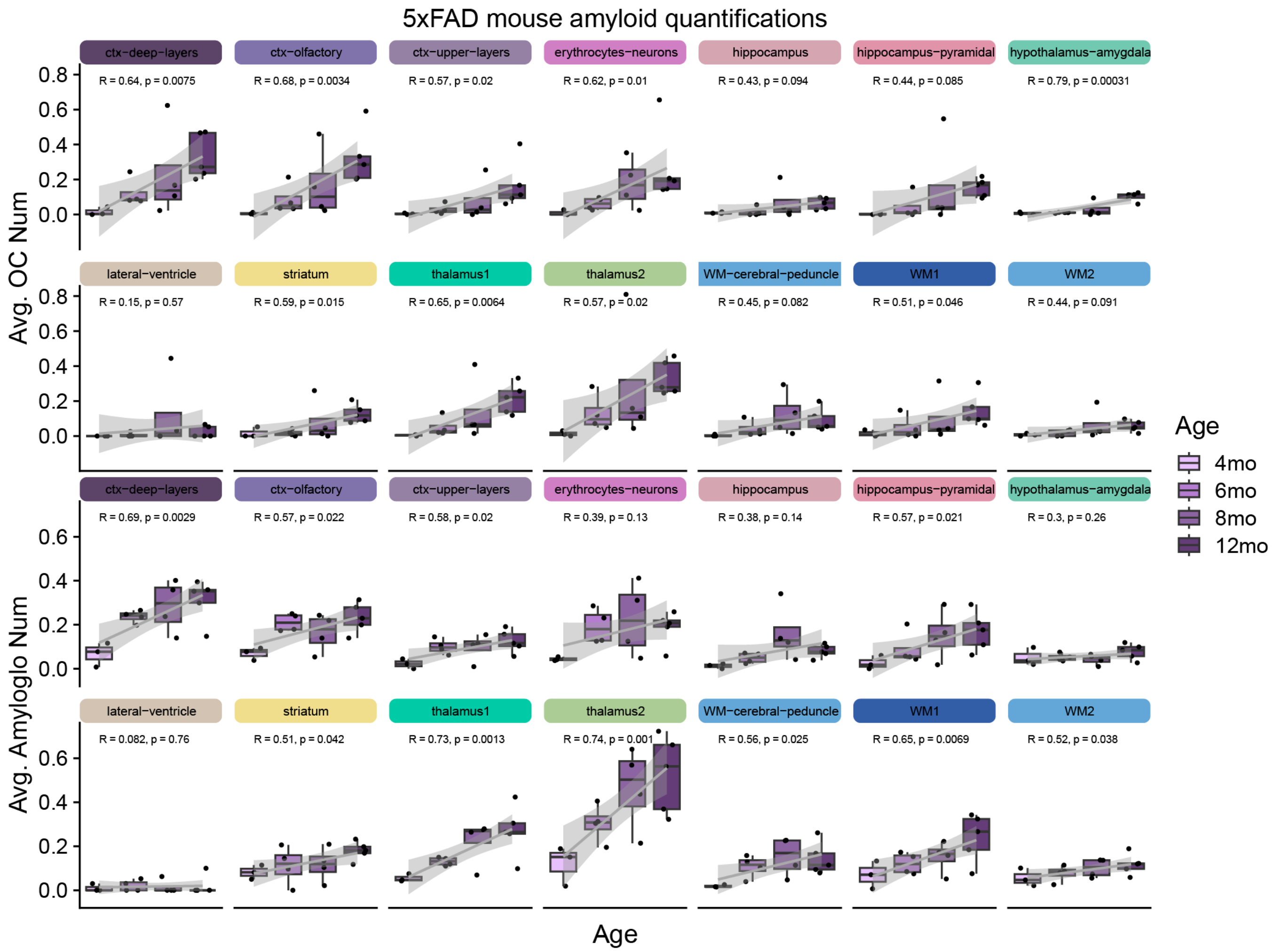

Supplementary Fig. 51 | Quantification of amyloid plaques in 5xFAD ST clusters. Box and whisker plots showing the distribution of amyloid quantifications in the 5xFAD mouse ST dataset, stratifying samples by their neuropathological plaque staging. Box boundaries and line correspond to the interquartile range (IQR) and median, respectively. Whiskers extend to the lowest or highest data points that are no further than 1.5 times the IQR from the box boundaries. Linear regression and 95% confidence interval around the mean are shown for each cluster. Pearson correlation results (coefficient and p-value) between age and amyloid burden are shown separately for each cluster. Amyloid information was obtained only for a subset of the mice within the ST dataset, the number of mice per group are as follows; 4 months: n=3; 6 months: n=4; 8 months: n=4; 12 months: n=5.

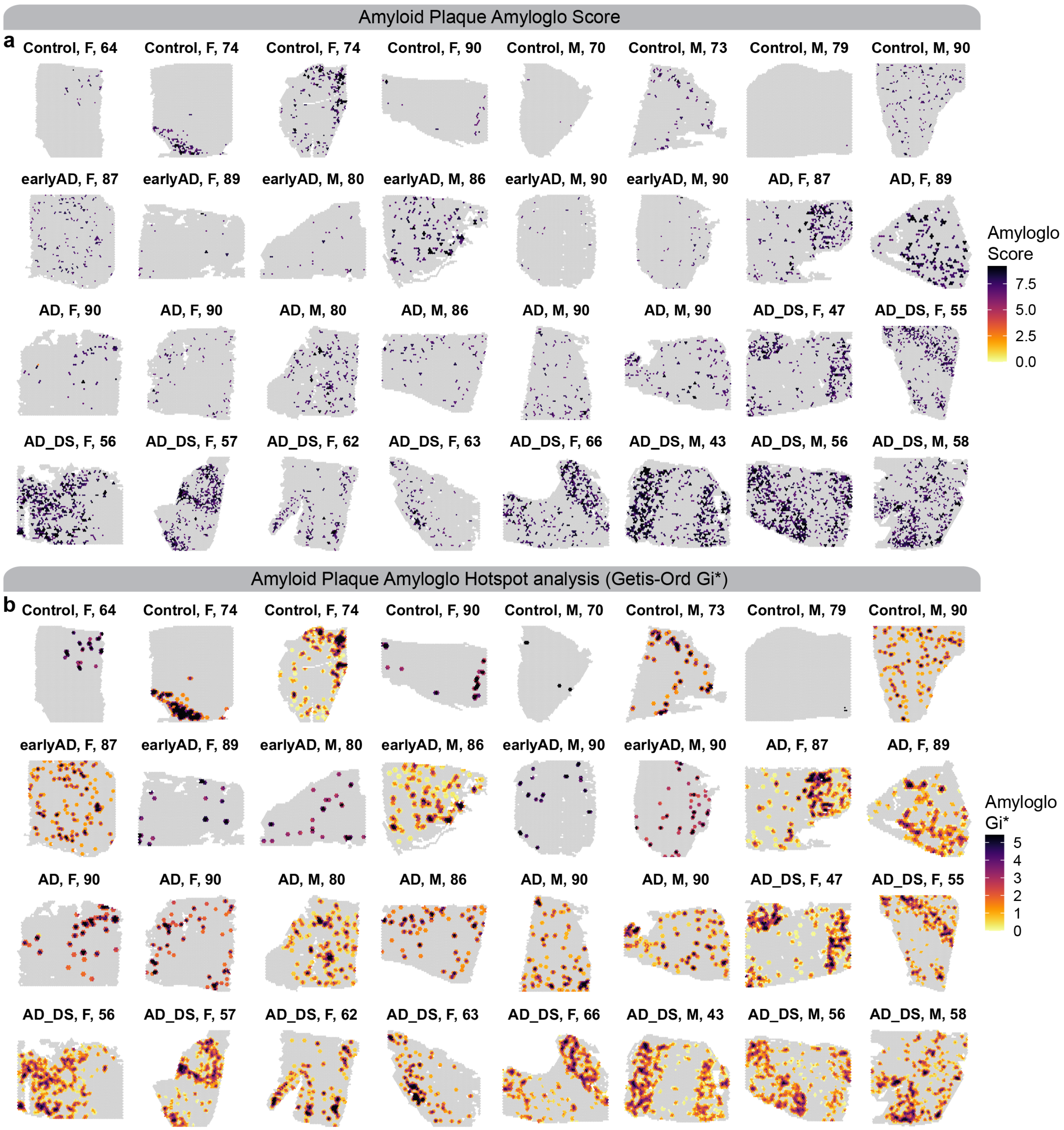

Supplementary Fig. 52 | Integration of amyloid plaque imaging (Amylo-glo) and hotspot analysis in the human ST dataset. Spatial feature plots for the human ST dataset showing the integration of amyloid plaque imaging of Amylo-glo. a, The Amylo-glo score, computed as the sum of the areas (size) for all overlapping amyloid plaques with each ST spot, and then log normalized. b, Getis-Ord Gi* hotspot analysis based on the Amylo-glo score.

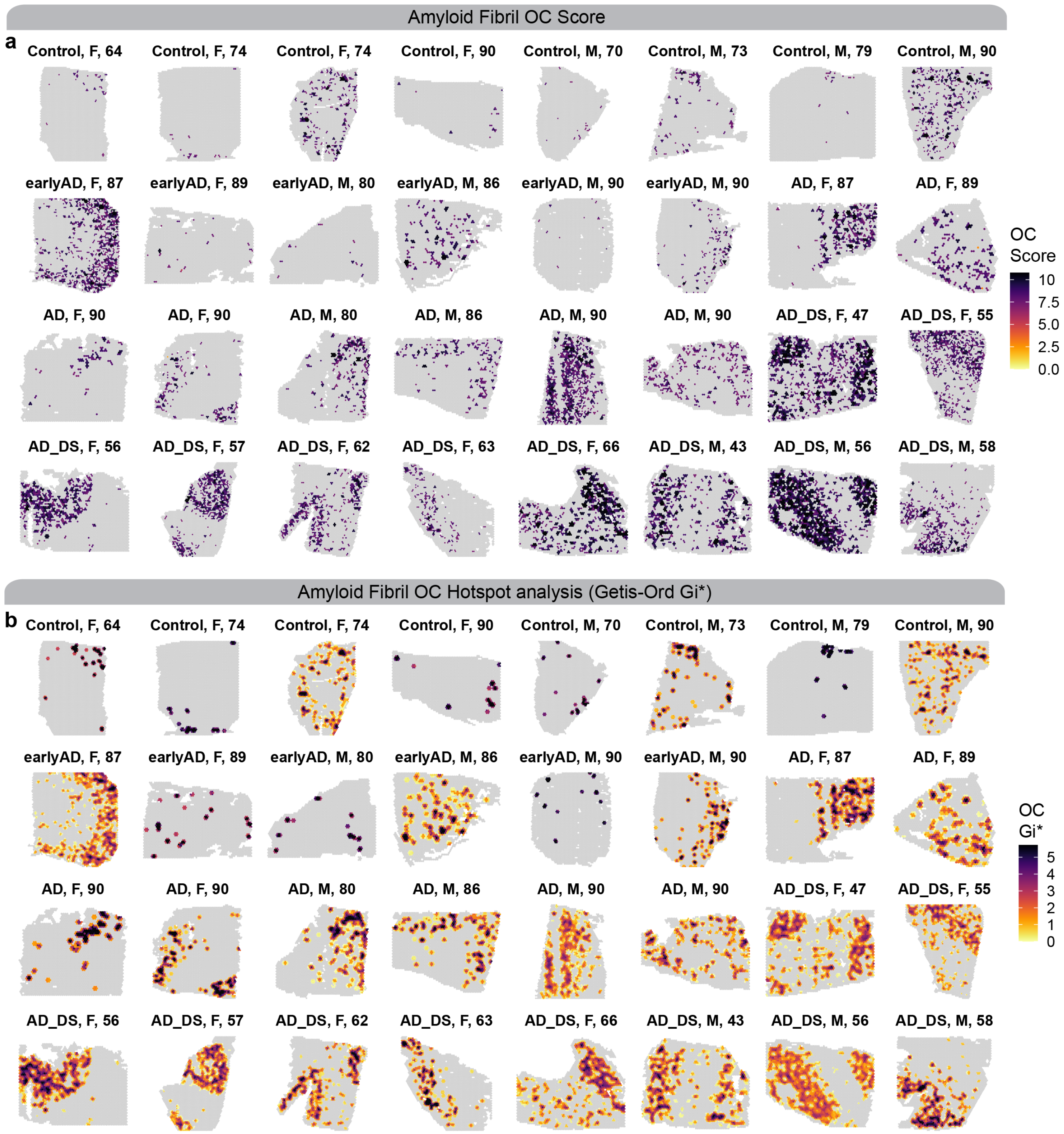

Supplementary Fig. 53 | Integration of amyloid fibril imaging (OC) and hotspot analysis in the human ST dataset. Spatial feature plots for the human ST dataset showing the integration of amyloid fibril imaging of OC. a, The OC score, computed as the sum of the areas (size) for all overlapping amyloid plaques with each ST spot, and then log normalized. b, Getis-Ord Gi* hotspot analysis based on the OC score.

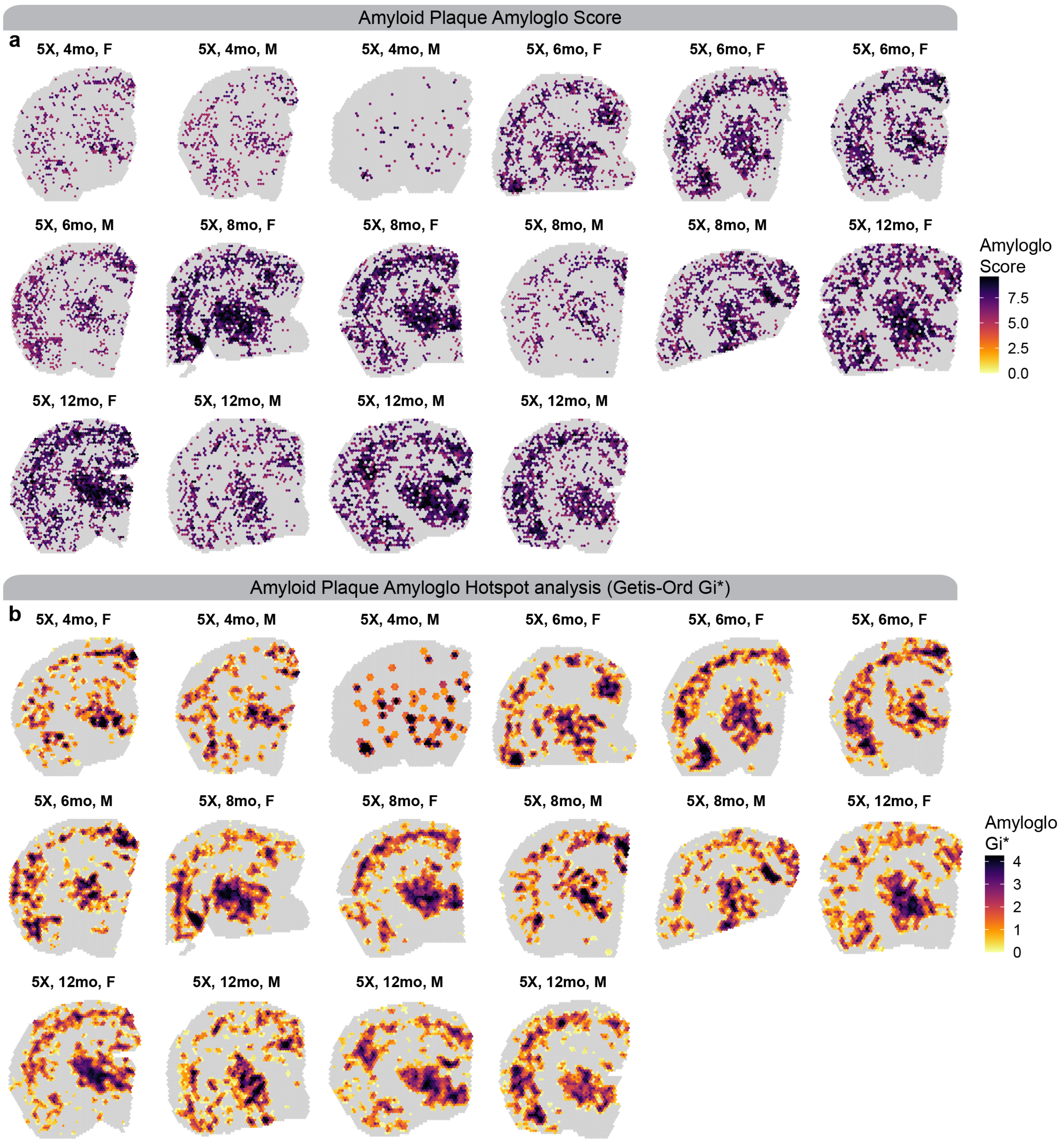

Supplementary Fig. 54 | Integration of amyloid plaque imaging (Amylo-glo) and hotspot analysis in the mouse ST dataset. Spatial feature plots for the mouse ST dataset showing the integration of amyloid plaque imaging of Amylo-glo. a, The Amylo-glo score, computed as the sum of the areas (size) for all overlapping amyloid plaques with each ST spot, and then log normalized. b, Getis-Ord Gi* hotspot analysis based on the Amylo-glo score.

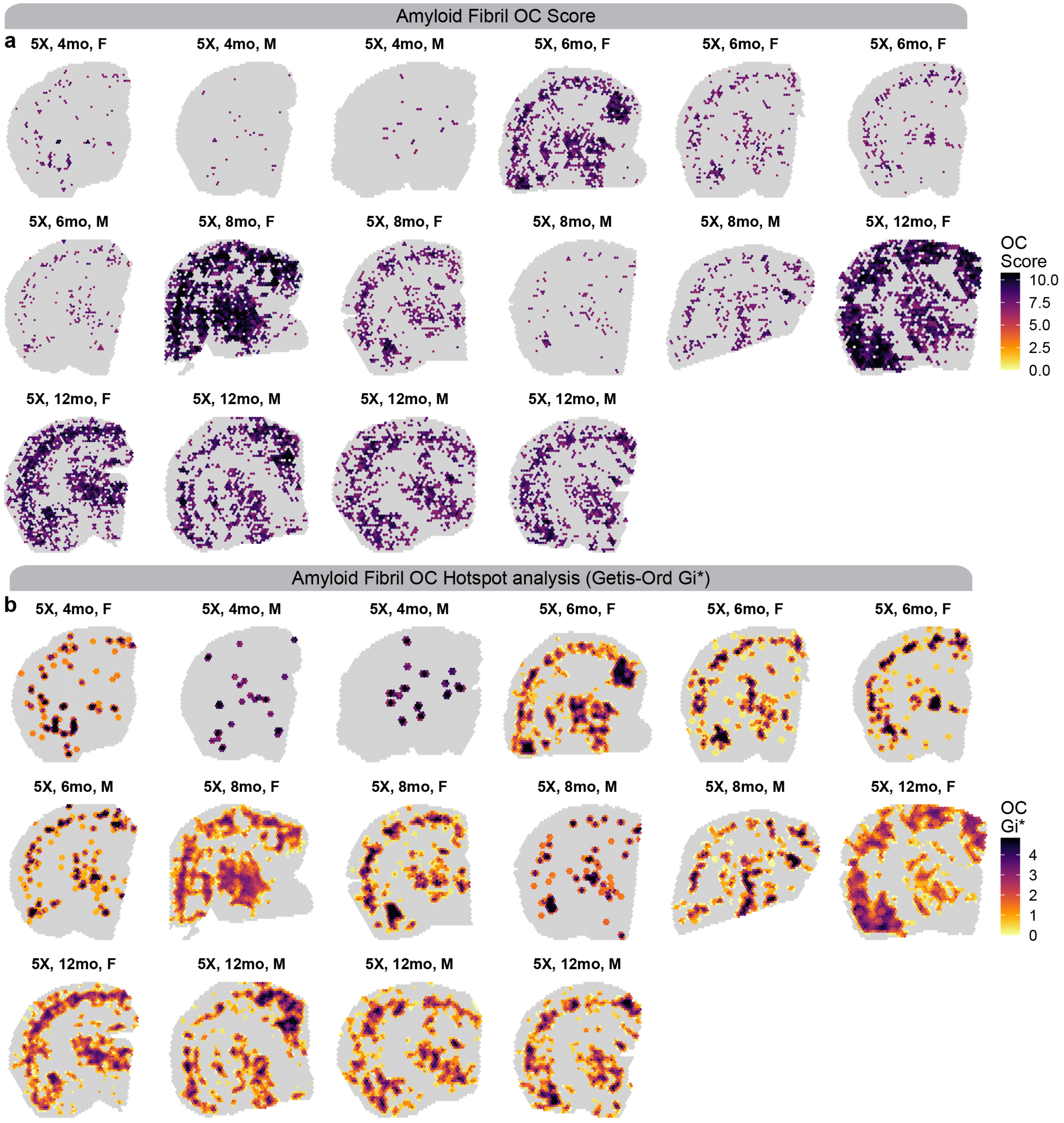

Supplementary Fig. 55 | Integration of amyloid fibril imaging (OC) and hotspot analysis in the mouse ST dataset. Spatial feature plots for the mouse ST dataset showing the integration of amyloid fibril imaging of OC. a, The OC score, computed as the sum of the areas (size) for all overlapping amyloid plaques with each ST spot, and then log normalized. b, Getis-Ord Gi* hotspot analysis based on the OC score.

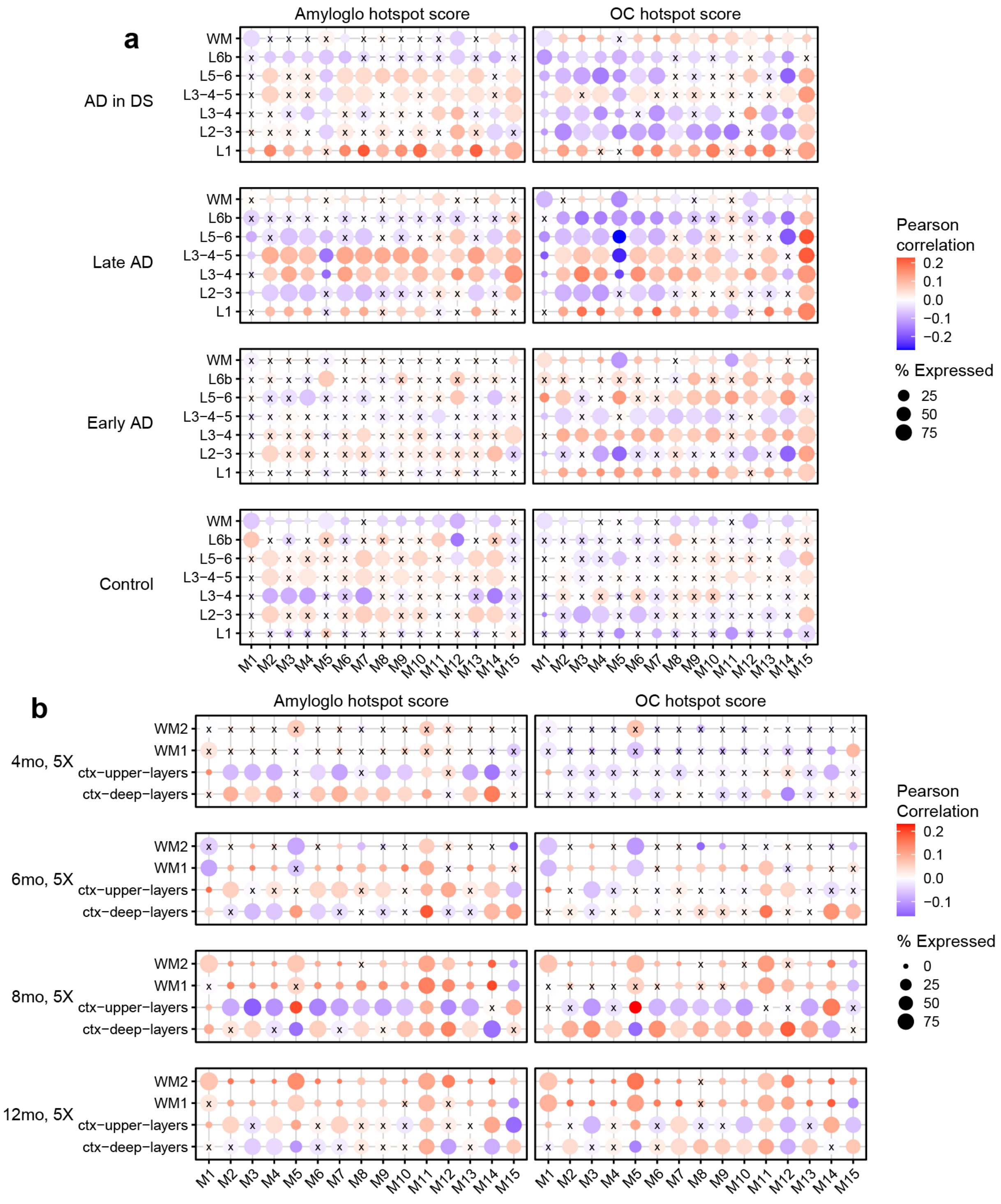

Supplementary Fig. 56 | Correlation of amyloid hotspots and co-expression module eigengenes. Dot plots show the percentage of ST spots expressing the module in each group as the size, and the Pearson correlation between the module and the amyloid hotspot score as the color. "x" symbol indicates that the correlation was not statistically significant (FDR > 0.05). This analysis is shown for the human ST dataset (**a**) and the mouse ST dataset (**b**).

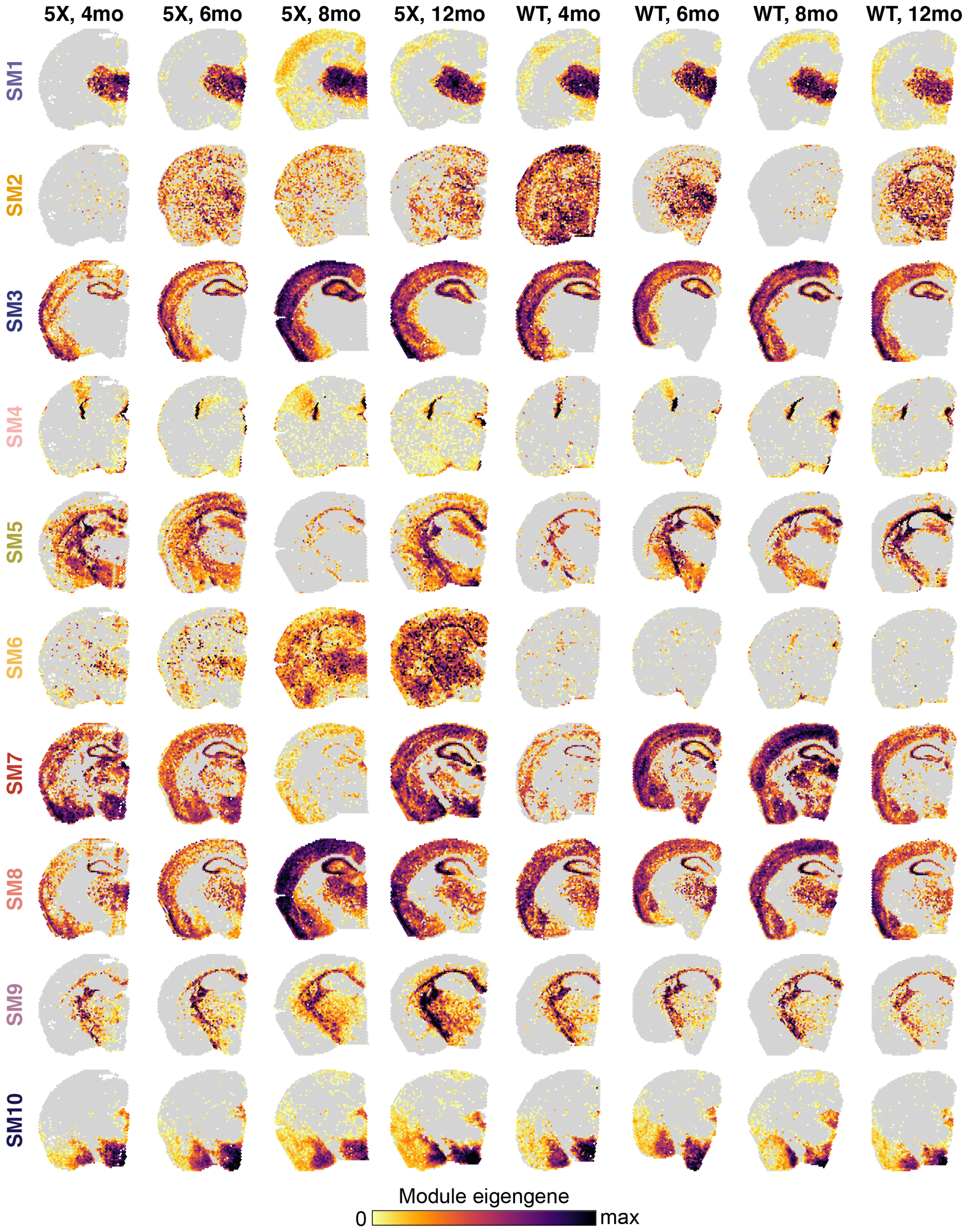

Supplementary Fig. 57 | Module eigengenes in representative samples from the mouse ST dataset. Spatial feature plots showing module eigengenes (MEs) for the ten mouse co-expression modules shown in eight representative samples.

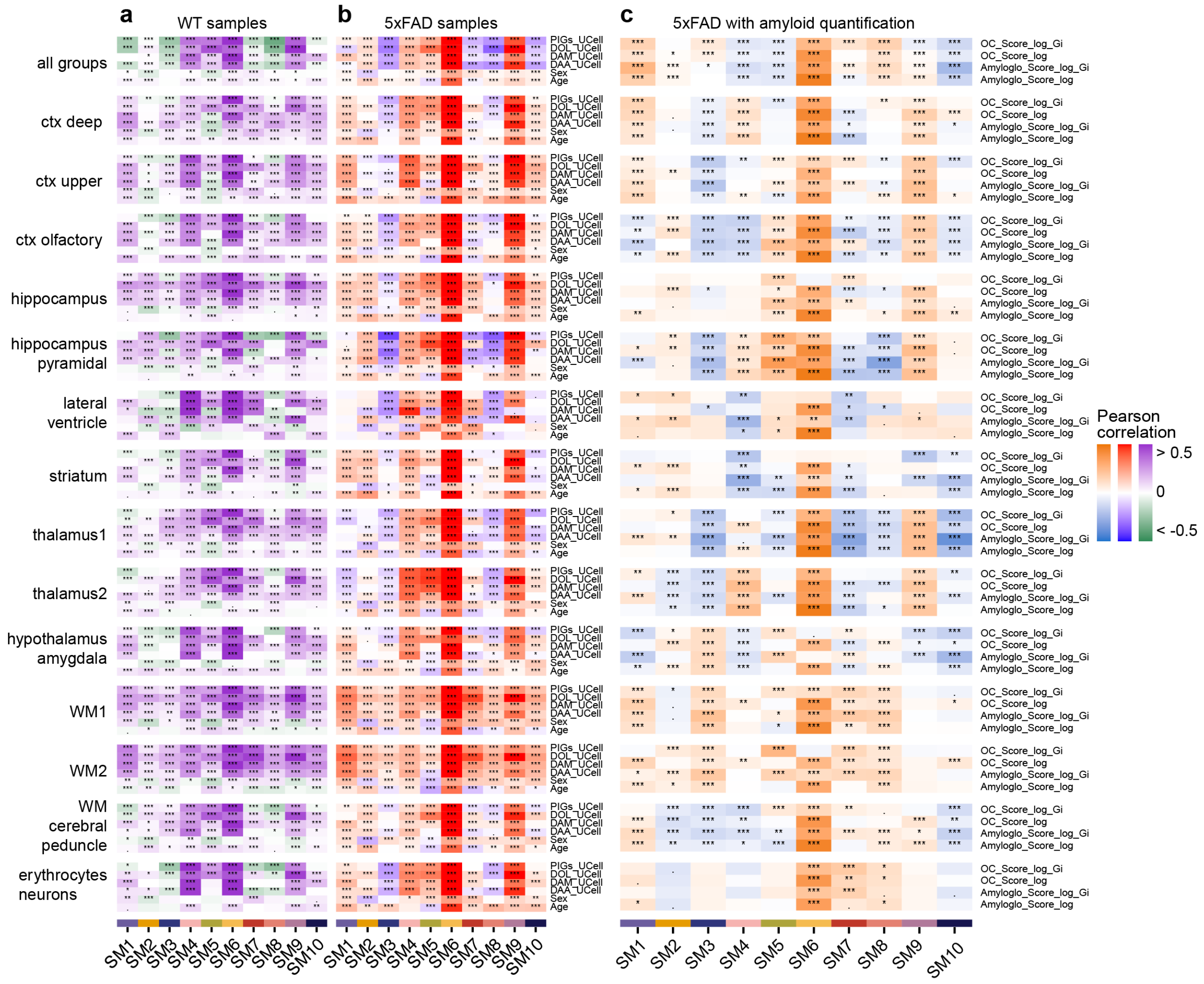

Supplementary Fig. 58 | Module-trait correlation analysis in the mouse co-expression network. a-b, Heatmap showing the module-trait correlation results for age, sex (positive correlation corresponds to female), and gene signatures for plaque-induced genes (PIGs), disease-associated oligodendrocytes (DOL), disease-associated microglia (DAM), and disease-associated astrocytes (DAA) in wild type (a) and in 5xFAD (b) mice. c, Heatmap showing the module-trait correlation results with the amyloid imaging analysis (Amylo-glo and OC) in 5xFAD with amyloid quantifications. Not significant (ns), p > 0.05; * p <= 0.05; ** p <= 0.01; *** p <= 0.001, **** p<= 0.0001.

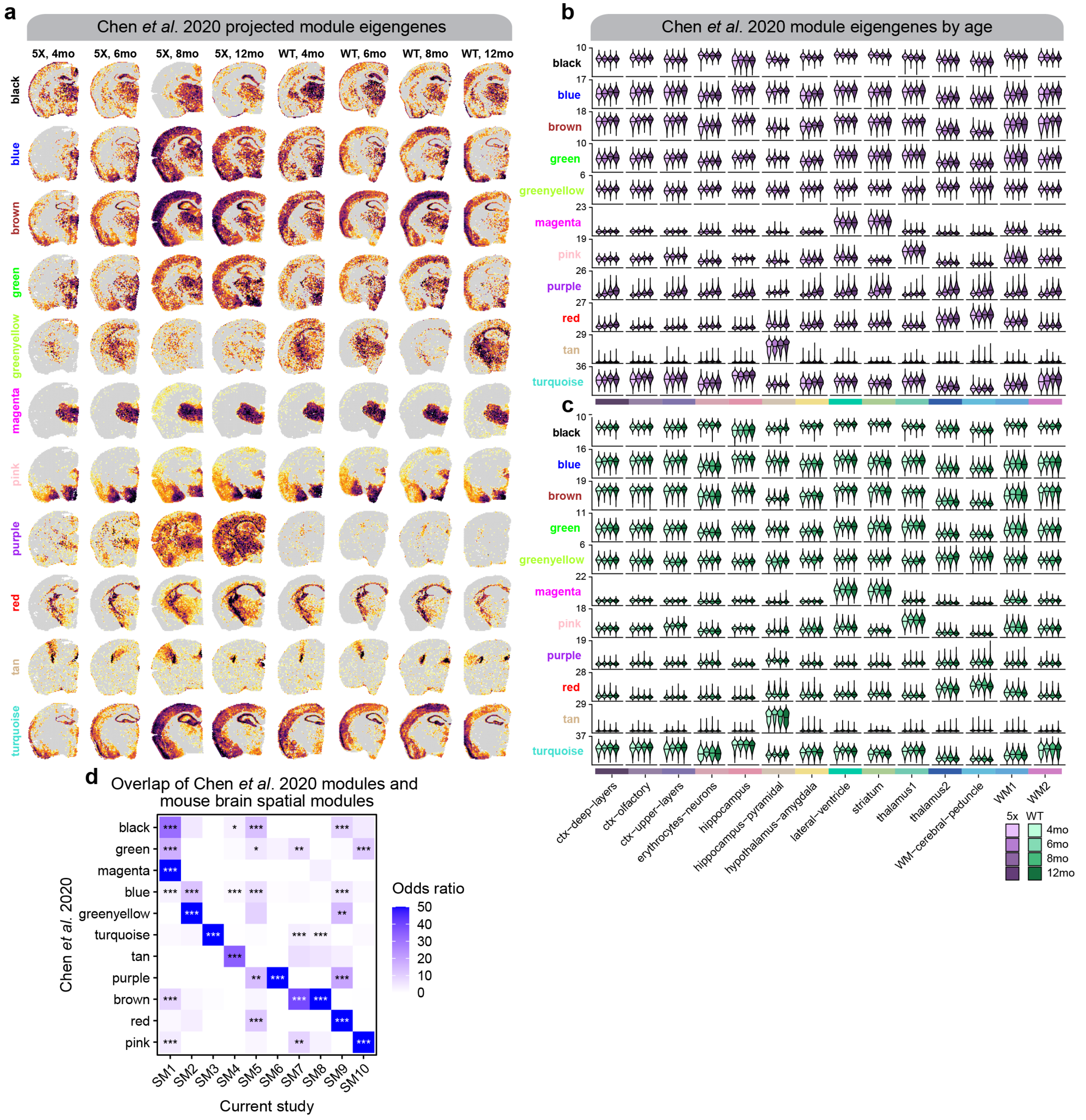

Supplementary Fig. 59 | Investigating the gene co-expression modules from Chen et al in our 5xFAD ST dataset. a, Spatial feature plots showing module eigengenes (MEs) for the ten mouse co-expression modules shown in eight representative samples. **b-c**, Module eigengene (ME) distributions for the ten mouse co-expression modules in each mouse age group (control, early-stage AD, late-stage AD, AD in DS) stratified by cluster for 5xFAD (**b**) and 5xFAD mice (**c**). **d**, Heatmap showing gene set overlap analysis results comparing the sets of genes from the mouse ST co-expression modules with the co-expression modules identified from the Chen et al.[23] study. Fisher's exact test results shown as follows: Not significant (ns), p > 0.05; * p <= 0.05; ** p <= 0.01; *** p <= 0.001, **** p<= 0.0001.

References

1. Marsh, S. E. *et al.* Dissection of artifactual and confounding glial signatures by single-cell sequencing of mouse and human brain. *Nature Neuroscience* **25**, 306–316 (2022).
2. Andreatta, M. & Carmona, S. J. UCell: Robust and scalable single-cell gene signature scoring. *Computational and Structural Biotechnology Journal* **19**, 3796–3798 (2021).
3. Dann, E., Henderson, N. C., Teichmann, S. A., Morgan, M. D. & Marioni, J. C. Differential abundance testing on single-cell data using k-nearest neighbor graphs. *Nature Biotechnology* 1–9 (2021).
4. Yang, A. C. *et al.* A human brain vascular atlas reveals diverse mediators of Alzheimer's risk. *Nature* **603**, 885–892 (2022).
5. Sweeney, M. D., Sagare, A. P. & Zlokovic, B. V. Blood–brain barrier breakdown in Alzheimer disease and other neurodegenerative disorders. *Nature Reviews Neurology* **14**, 133–150 (2018).
6. Farnsworth, B. *et al.* Gene Expression of Quaking in Sporadic Alzheimer's Disease Patients is Both Upregulated and Related to Expression Levels of Genes Involved in Amyloid Plaque and Neurofibrillary Tangle Formation. *Journal of Alzheimer's Disease* **53**, 209–219 (2016).
7. Hodge, R. D. *et al.* Conserved cell types with divergent features in human versus mouse cortex. *Nature* **573**, 61–68 (2019).
8. Zeng, H. *et al.* Large-Scale Cellular-Resolution Gene Profiling in Human Neocortex Reveals Species-Specific Molecular Signatures. *Cell* **149**, 483–496 (2012).
9. Keren-Shaul, H. *et al.* A Unique Microglia Type Associated with Restricting Development of Alzheimer's Disease. *Cell* **169**, 1276–1290.e17 (2017).
10. Habib, N. *et al.* Disease-associated astrocytes in Alzheimer's disease and aging. *Nature Neuroscience* **23**, 701–706 (2020).
11. Kenigsbuch, M. *et al.* A shared disease-associated oligodendrocyte signature among multiple CNS pathologies. *Nature Neuroscience* **25**, 876–886 (2022).
12. Hartl, C. L. *et al.* Coexpression network architecture reveals the brain-wide and multiregional basis of disease susceptibility. *Nature Neuroscience* 1–11 (2021).
13. Brown, N. *et al.* Neuronal Hemoglobin Expression and Its Relevance to Multiple Sclerosis Neuropathology. *Journal of Molecular Neuroscience* **59**, 1–17 (2016).
14. Chen, C. *et al.* Imaging mass cytometry reveals generalised deficiency in OXPHOS complexes in Parkinson's disease. *npj Parkinson's Disease* **7**, 39 (2021).
15. Wei, R. *et al.* Spatial charting of single-cell transcriptomes in tissues. *Nature Biotechnology* 1–10 (2022).
16. Sedmak, G. & Judaš, M. White Matter Interstitial Neurons in the Adult Human Brain: 3% of Cortical Neurons in Quest for Recognition. *Cells* **10**, 190 (2021).
17. Jin, S. *et al.* Inference and analysis of cell-cell communication using CellChat. *Nature Communications* **12**, 1088 (2021).
18. Dimitrov, D. *et al.* Comparison of methods and resources for cell-cell communication inference from single-cell RNA-Seq data. *Nature Communications* **13**, 3224 (2022).
19. (ADGC), A. D. G. C. *et al.* Genetic meta-analysis of diagnosed Alzheimer's disease identifies new risk loci and implicates Aβ, tau, immunity and lipid processing. *Nature Genetics* **51**, 414–430 (2019).
20. (EADI), E. A. D. I. *et al.* Meta-analysis of 74,046 individuals identifies 11 new susceptibility loci for Alzheimer's disease. *Nature Genetics* **45**, 1452–1458 (2013).
21. Rathore, N. *et al.* Paired Immunoglobulin-like Type 2 Receptor Alpha G78R variant alters ligand binding and confers protection to Alzheimer's disease. *PLoS Genetics* **14**, e1007427 (2018).
22. Lindman, K. L. *et al.* PILRA polymorphism modifies the effect of APOE4 and GM17 on Alzheimer's disease risk. *Scientific Reports* **12**, 13264 (2022).
23. Chen, W.-T. *et al.* Spatial Transcriptomics and In Situ Sequencing to Study Alzheimer's Disease. *Cell* **182**, 976–991.e19 (2020).
24. Getis, A. & Ord, J. K. The Analysis of Spatial Association by Use of Distance Statistics. *Geographical Analysis* **24**, 189–206 (1992).
25. Ord, J. K. & Getis, A. Local Spatial Autocorrelation Statistics: Distributional Issues and an Application. *Geographical Analysis* **27**, 286–306 (1995).
26. Moses, L. *et al.* Voyager: exploratory single-cell genomics data analysis with geospatial statistics. *bioRxiv* (2023).
27. Morabito, S., Reese, F., Rahimzadeh, N., Miyoshi, E. & Swarup, V. hdWGCNA identifies co-expression networks in high-dimensional transcriptomics data. *Cell Reports Methods* (2023).
28. Moses, L. & Pachter, L. Museum of spatial transcriptomics. *Nature Methods* 1–13 (2022).
29. Rodriques, S. G. *et al.* Slide-seq: A scalable technology for measuring genome-wide expression at high spatial resolution. *Science* **363**, 1463–1467 (2019).
30. Srivatsan, S. R. *et al.* Embryo-scale, single-cell spatial transcriptomics. *Science* **373**, 111–117 (2021).
31. Ståhl, P. L. *et al.* Visualization and analysis of gene expression in tissue sections by spatial transcriptomics. *Science* **353**, 78–82 (2016).
32. Wang, X. *et al.* Three-dimensional intact-tissue sequencing of single-cell transcriptional states. *Science* **361** (2018).
33. Ke, R. *et al.* In situ sequencing for RNA analysis in preserved tissue and cells. *Nature Methods* **10**, 857–860 (2013).
34. Bellenguez, C. *et al.* New insights into the genetic etiology of Alzheimer's disease and related dementias. *Nature Genetics* 1–25 (2022).
35. Jansen, I. E. *et al.* Genome-wide meta-analysis identifies new loci and functional pathways influencing Alzheimer's disease risk. *Nature Genetics* **51**, 404–413 (2019).
36. Lopes, K. d. P. *et al.* Genetic analysis of the human microglial transcriptome across brain regions, aging and disease pathologies. *Nature Genetics* **54**, 4–17 (2022).
37. Bryois, J. *et al.* Cell-type-specific cis-eQTLs in eight human brain cell types identify novel risk genes for psychiatric and neurological disorders. *Nature Neuroscience* **25**, 1104–1112 (2022).
38. Boyle, E. A., Li, Y. I. & Pritchard, J. K. An Expanded View of Complex Traits: From Polygenic to Omnigenic. *Cell* **169**, 1177–1186 (2017).
39. Cusanovich, D. A. *et al.* A Single-Cell Atlas of In Vivo Mammalian Chromatin Accessibility. *Cell* **174**, 1309–1324.e18 (2018).
40. Nott, A. *et al.* Brain cell type–specific enhancer–promoter interactome maps and disease-risk association. *Science* **366**, 1134–1139 (2019).
41. Corces, M. R. *et al.* Single-cell epigenomic analyses implicate candidate causal variants at inherited risk loci for Alzheimer's and Parkinson's diseases. *Nature Genetics* **52**, 1158–1168 (2020).
42. Morabito, S. *et al.* Single-nucleus chromatin accessibility and transcriptomic characterization of Alzheimer's disease. *Nature Genetics* **53**, 1143–1155 (2021).
43. McQuade, A. *et al.* Gene expression and functional deficits underlie TREM2-knockout microglia responses in human models of Alzheimer's disease. *Nature Communications* **11**, 5370 (2020).
44. Zhang, M. J. *et al.* Polygenic enrichment distinguishes disease associations of individual cells in single-cell RNA-seq data. *Nature Genetics* 1–9 (2022).
45. Seyfried, N. T. *et al.* A Multi-network Approach Identifies Protein-Specific Co-expression in Asymptomatic and Symptomatic Alzheimer's Disease. *Cell Systems* **4**, 60–72.e4 (2017).
46. Simon, M. J. *et al.* Transcriptional network analysis of human astrocytic endfoot genes reveals region-specific associations with dementia status and tau pathology. *Scientific Reports* **8**, 12389 (2018).
47. Shijo, M. *et al.* Association of adipocyte enhancer-binding protein 1 with Alzheimer's disease pathology in human hippocampi. *Brain Pathology* **28**, 58–71 (2018).
48. Zhou, Y. *et al.* Human and mouse single-nucleus transcriptomics reveal TREM2-dependent and TREM2-independent cellular responses in Alzheimer's disease. *Nature Medicine* **26**, 131–142 (2020).
49. Thrupp, N. *et al.* Single-Nucleus RNA-Seq Is Not Suitable for Detection of Microglial Activation Genes in Humans. *Cell Reports* **32**, 108189 (2020).
50. Neuner, S. M., Heuer, S. E., Huentelman, M. J., O'Connell, K. M. & Kaczorowski, C. C. Harnessing Genetic Complexity to Enhance Translatability of Alzheimer's Disease Mouse Models: A Path toward Precision Medicine. *Neuron* **101**, 399–411.e5 (2019).
51. Telpoukhovskaia, M. A. *et al.* Conserved cell-type specific signature of resilience to Alzheimer's disease nominates role for excitatory intratelencephalic cortical neurons. *bioRxiv* 2022.04.12.487877 (2023).
52. Zhao, E. *et al.* Spatial transcriptomics at subspot resolution with BayesSpace. *Nature Biotechnology* 1–10 (2021).
53. Finak, G. *et al.* MAST: a flexible statistical framework for assessing transcriptional changes and characterizing heterogeneity in single-cell RNA sequencing data. *Genome Biology* **16**, 278 (2015).
54. Mathys, H. *et al.* Single-cell transcriptomic analysis of Alzheimer's disease. *Nature* **570**, 332–337 (2019).
55. Elosua-Bayes, M., Nieto, P., Mereu, E., Gut, I. & Heyn, H. SPOTlight: seeded NMF regression to deconvolute spatial transcriptomics spots with single-cell transcriptomes. *Nucleic Acids Research* **49**, e50–e50 (2021).

Alzheimer's Biomarkers Consortium-Down Syndrome (ABC-DS)

University of California, Irvine
Eric Doran[8], Elizabeth Head[2,9], Christy Hom[11], David Keator[11], Ira T. Lott[2,8], Mark Mapstone[12], Wayne P. Silverman[9], Batool Rizvi[1] & Michael A. Yassa[1]

University of Pittsburgh
Howard J. Aizenstein[13], Peter Bulova[14], Ann D. Cohen[13], Eleanor Feingold[15], Benjamin L. Handen[13], Milos D. Ikonomovic[16] M. Ilyas Kamboh[15], William E. Klunk[13], Julia K. Kofler[17], Charles Laymon[18], Chester A. Mathis[18], Davneet Singh Minhas[18], Neelesh Nadkarni[14], Julie C. Price[rad], Dana L. Tudorascu[13] & Rameshwari Tumuluru[13]

Washington University in St. Louis
Beau M. Ances[19], John N. Constantino[20] Anne Fagan[19], Rachel Henson[19], Courtney Jordan[19] & Desiree A. White[21]

Columbia University
Howard F. Andrews[22], Karen Bell[23], Adam M. Brickman[24], Lorraine Clark[25], Patrick Lao[24], Lawrence Honig[23], William C. Kreisl[26], Joseph H. Lee[27], Deborah Pang[28], Nicole Schupf[27], Benjamin Tycko[29] & Badri Varadarajan[28]

University of Wisconsin-Madison
Rasmus M. Birn[30], Bradley T. Christian[31], Sigan L. Hartley[31] & Sterling C. Johnson[32]

University of Cambridge
Isabel Clare[33], Victoria Lupson[34] & Shahid Zaman[35]

University of Kentucky
Jordan Harp[36] & Frederick Schmitt[36]

Georgetown University
Amrita Cheema[37]

Banner Alzheimer's Institute
Kewei Chen[38] & Eric Reiman[38]

Indiana University
Tatiana M. Foroud[39]

Johns Hopkins University
Mei-Cheng Wang[40]

University of North Texas Health Science Center
Sid O'Bryant[41], Melissa Petersen[42] & Fan Zhang[42]

Massachusetts General Hospital, Harvard Medical School
Florence Lai[43], Margaret Pulsifer[44] & H. Diana Rosas[43]

New York State Institute for Basic Research in Developmental Disabilities
Sharon Krinsky-McHale[45]

University of Southern California
Michael S. Rafii[46]

National Institutes of Health (NIH)
Melissa Parisi[47] & Laurie Ryan[48]

[11]Department of Psychiatry & Human Behavior, University of California Irvine School of Medicine, Orange, CA, USA
[12]Department of Neurology, University of California Irvine School of Medicine, Irvine, CA, USA
[13]Department of Psychiatry, University of Pittsburgh, Pittsburgh, PA, USA
[14]Department of Medicine, University of Pittsburgh, Pittsburgh, PA, USA

[15]Department of Human Genetics, Graduate School of Public Health, University of Pittsburgh, Pittsburgh, PA, USA
[16]Department of Neurology, University of Pittsburgh, Pittsburgh, PA, USA
[17]Department of Radiology, University of Pittsburgh, Pittsburgh, PA, USA
[18]Department of Pathology, University of Pittsburgh, Pittsburgh, PA, USA
[19]Department of Neurology, Washington University in St Louis, St Louis, MO, USA
[20]Department of Psychiatry and Pediatrics, Washington University in St Louis, St Louis, MO, USA
[21]Department of Psychological and Brain Sciences, Washington University in St Louis, St Louis, MO, USA
[22]Department of Biostatistics, Columbia University, New York, NY, USA
[23]Department of Neurology, Aging & Dementia, Columbia University, New York, NY, USA
[24]Department of Neuropsychology, Columbia University, New York, NY, USA
[25]Department of Obstetrics & Gynecology Research, Columbia University, New York, NY, USA
[26]Taub Institute for Research on Alzheimer's Disease & the Aging Brain, Columbia University, New York, NY, USA
[27]Department of Epidemiology, Columbia University, New York, NY, USA
[28]Gertrude H. Sergievsky Center, Columbia University, New York, NY, USA
[29]Department of Pathology & Cell Biology, Columbia University, New York, NY, USA
[30]Department of Psychiatry, University of Wisconsin-Madison, Madison, WI, USA
[31]Waisman Center, University of Wisconsin-Madison, Madison, WI, USA
[32]Department of Medicine, University of Wisconsin-Madison, Madison, WI, USA
[33]Department of Psychiatry, University of Cambridge, Cambridge, UK
[34]Wolfson Brain Imaging Centre, University of Cambridge, Cambridge, UK
[35]Intellectual Disability Research Group, University of Cambridge, Cambridge, UK
[36]Department of Neurology, University of Kentucky, Lexington, KY, USA
[37]Department of Oncology, Georgetown University School of Medicine, Washington, DC, USA
[38]Banner Alzheimer's Institute, Phoenix, AZ, USA
[39]Department of Medical & Molecular Genetics, Indiana University, Indianapolis, IN, USA
[40]Department of Biostatistics, Bloomberg School of Public Health, Johns Hopkins University, Baltimore, MD, USA
[41] Institute for Translational Research, Department of Pharmacology and Neuroscience, University of North Texas Health Science Center, Fort Worth, TX, USA
[42] Department of Family Medicine, University of North Texas Health Science Center, Fort Worth, TX, USA
[43] Department of Neurology, Massachusetts General Hospital, Harvard Medical School, Boston, MA, USA
[44] Department of Psychiatry, Massachusetts General Hospital, Harvard Medical School, Boston, MA, USA
[45] Department of Psychology, New York State Institute for Basic Research in Developmental Disabilities, New York, NY, USA
[46] Alzheimer's Therapeutic Research Institute, Keck School of Medicine, University of Southern California, Los Angeles, CA, USA
[47] Intellectual and Developmental Disabilities Branch, National Institute on Aging, Eunice Kennedy Shriver National Institute of Child Health and Human Development (NICHD), National Institutes of Health, Bethesda, MD, USA
[48] Division of Neuroscience, National Institute on Aging (NIA), National Institutes of Health, Bethesda, MD, USA
