# Supplementary material for: Spatial and single-nucleus transcriptomic analysis of genetic and sporadic forms of Alzheimer’s disease
Source: Nat Genet. 2024 Nov 22;56(12):2704–17. doi: 10.1038/s41588-024-01961-x (PMC11631771; doi:10.1038/s41588-024-01961-x)
Supplement: Supplementary file 2 — Reporting Summary [file 41588_2024_1961_MOESM2_ESM.pdf]

nature portfolio

Corresponding author(s): Vivek Swarup

Last updated by author(s): Aug 8, 2024

Reporting Summary

Nature Portfolio wishes to improve the reproducibility of the work that we publish. This form provides structure for consistency and transparency in reporting. For further information on Nature Portfolio policies, see our Editorial Policies and the Editorial Policy Checklist.

Statistics

For all statistical analyses, confirm that the following items are present in the figure legend, table legend, main text, or Methods section.

n/a	Confirmed	
☐	☒	The exact sample size (*n*) for each experimental group/condition, given as a discrete number and unit of measurement
☐	☒	A statement on whether measurements were taken from distinct samples or whether the same sample was measured repeatedly
☐	☒	The statistical test(s) used AND whether they are one- or two-sided<br>*Only common tests should be described solely by name; describe more complex techniques in the Methods section.*
☐	☒	A description of all covariates tested
☐	☒	A description of any assumptions or corrections, such as tests of normality and adjustment for multiple comparisons
☐	☒	A full description of the statistical parameters including central tendency (e.g. means) or other basic estimates (e.g. regression coefficient) AND variation (e.g. standard deviation) or associated estimates of uncertainty (e.g. confidence intervals)
☐	☒	For null hypothesis testing, the test statistic (e.g. *F*, *t*, *r*) with confidence intervals, effect sizes, degrees of freedom and *P* value noted<br>*Give P values as exact values whenever suitable.*
☒	☐	For Bayesian analysis, information on the choice of priors and Markov chain Monte Carlo settings
☒	☐	For hierarchical and complex designs, identification of the appropriate level for tests and full reporting of outcomes
☐	☒	Estimates of effect sizes (e.g. Cohen's *d*, Pearson's *r*), indicating how they were calculated

Our web collection on statistics for biologists contains articles on many of the points above.

Software and code

Policy information about availability of computer code

Data collection	No software was used for data collection
Data analysis	All analysis was done in R (v4.0 and higher) or python (v 3.6 or higher). All software used in the analysis is described in materials and methods section and code used is available in swaruplab github. snRNA-seq and spatial transcriptomics analyses were conducted using Spaceranger (v 1.2.1), Scanpy (v1.8), BayesSpace (v 1.10.1), CellTrek (v 0.0.94), CellChat (v 1.1.3), UCell R package (v 2.2.0), monocle3 (v 1.3.1), EnrichR (v 3.1), GeneOverlap (v 1.34.0), Seurat (v 4.3.0), Voyager (v 1.0.10), UpSetR (v 1.4.0), hdWGCNA (v 0.2.19), Cellbender (v 0.2.0), Scrublet (v 0.2.3), scDRS (v 1.0.0), MiloR (v 1.8.1), and LIANA (v 0.1.13) softwares. Spatial proteomic analysis was done using Seurat (v 4.3.0).

For manuscripts utilizing custom algorithms or software that are central to the research but not yet described in published literature, software must be made available to editors and reviewers. We strongly encourage code deposition in a community repository (e.g. GitHub). See the Nature Portfolio guidelines for submitting code & software for further information.

Data

Policy information about availability of data

All manuscripts must include a data availability statement. This statement should provide the following information, where applicable:

- Accession codes, unique identifiers, or web links for publicly available datasets
- A description of any restrictions on data availability
- For clinical datasets or third party data, please ensure that the statement adheres to our policy

All data in this manuscript have been deposited in the NIH GEO database. GSE233208 can be accessed with token: aduxqegclhmjzcj.

Research involving human participants, their data, or biological material

Policy information about studies with human participants or human data. See also policy information about sex, gender (identity/presentation), and sexual orientation and race, ethnicity and racism.

Reporting on sex and gender	Sex was used as a covariate throughout the analysis. Case and control was balanced for sex and sex-specific differences are reported throughout the study.
Reporting on race, ethnicity, or other socially relevant groupings	Race and ethnicity data where available are provided. No separate analysis for race and ethnicity were performed.
Population characteristics	Population characteristics were individually collected and available clinical and demographic data including age and sex are available in Supplemental Table 1.
Recruitment	Human brain tissue from prefrontal cortex and posterior cingulate cortex was obtained from UC Irvine's Alzheimer's Disease Research Center and the NIH NeuroBioBank. Samples were assigned to groups based on both NFT and plaque staging, in addition to clinical diagnoses. Samples were also selected based upon several covariates, including age, sex, race, postmortem interval (PMI), RNA integrity number (RIN), and disease comorbidity.
Ethics oversight	Postmortem tissue were de-identified before acquisition and thus exempt from IRB approval. Exemption to this effect was obtained from UCI's Institutional Review Board (IRB).

Note that full information on the approval of the study protocol must also be provided in the manuscript.

Field-specific reporting

Please select the one below that is the best fit for your research. If you are not sure, read the appropriate sections before making your selection.

☒ Life sciences ☐ Behavioural & social sciences ☐ Ecological, evolutionary & environmental sciences

For a reference copy of the document with all sections, see nature.com/documents/nr-reporting-summary-flat.pdf

Life sciences study design

All studies must disclose on these points even when the disclosure is negative.

Sample size	Sample sizes were chosen based on sample availability and power analysis with previously available data (Morabito et al., Nature Genetics, 2021 and Chen et al ., Cell, 2020)
Data exclusions	We used network connectivity based outlier removal to remove outliers from the analysis. However the raw data included the outlier samples.
Replication	Additional samples were used for replication and where applicable (like snRNA-seq and spatial transcriptomics data) data were correlated by published datasets for replication,
Randomization	Randomization is not relevant for this study - blinded identity (de-identified) samples were obtained from brain banks and were given sample level metadata by the brain bank. when subset-level data was analyzed the subset was randomized.
Blinding	Blinding is not relevant for this study, samples were obtained from the brain banks.

Reporting for specific materials, systems and methods

We require information from authors about some types of materials, experimental systems and methods used in many studies. Here, indicate whether each material, system or method listed is relevant to your study. If you are not sure if a list item applies to your research, read the appropriate section before selecting a response.

Materials & experimental systems

n/a	Involved in the study
☐	☒ Antibodies
☒	☐ Eukaryotic cell lines
☒	☐ Palaeontology and archaeology
☐	☒ Animals and other organisms
☒	☐ Clinical data
☒	☐ Dual use research of concern
☒	☐ Plants

Methods

n/a	Involved in the study
☒	☐ ChIP-seq
☒	☐ Flow cytometry
☒	☐ MRI-based neuroimaging

Antibodies

Antibodies used

ANGPTL4 Antibody; Cat#710186; 1:500; ThermoFisher, GFAP Polyclonal Antibody; Cat#PA3- 16727; 1:500; ThermoFisher, CD99 antibody; Cat#MA5-12287; 1:250; ThermoFisher, CD99L2 antibody; Cat#PA5-58539; 1:500; ThermoFisher, Nectin 2 Antibody; Cat#PA582470;1:250;ThermoFisher, MAP2 Antibody; Cat#PA1-10005; 1:250; ThermoFisher, C1QB Polyclonal Antibody; Cat#PA5-42554; 1:250; ThermoFisher. For Imaging mass cytometry (Hyperion) data we used these antibodies - CD44, Clone#IM7, BioLegend, 5 μg/ml; rPTPRM, Clone#MAB4446, R&D Systems, 30 μg/ml; Moesin, Clone#MSN492, Biotium, 30 μg/ml; Cystatin C, Clone#MA5-29195, ThermoFisher, 25 μg/ml; B-Amyloid, Clone#6E10, BioLegend, 5 μg/ml; CD68, Clone#KP1, BioLegend, 10 μg/ml; MAP2, Clone#EPR19691, Abcam, 2 μg/ml; ERBIN, Clone#AF7866, R&D Systems, 40 μg/ml; BIN1, Clone#EPR13463-25, Abcam, 30 μg/ml; CD163, Clone#EPR19518, Abcam, 30 μg/ml; GFAP, Clone#2E1.E9, BioLegend, 2 μg/ml; Foxp2, Clone#AF5647, R&D Systems, 20 μg/ml; NeuN, Clone#D4G4O, Cell Signaling, 20 μg/ml; APOE, Clone#WUE-4, Novus, 20 μg/ml; Midkine, Clone#EP1143Y, Abcam, 40 μg/ml; CLP-1, Clone#AF2690, R&D Systems, 20 μg/ml; COL25A1, Clone#540802, R&D Systems, 40 μg/ml; GPC5, Clone#297716, R&D Systems, 40 μg/ml; pTau, Clone#AT8, ThermoFisher, 20 μg/ml; Iba1, Polyclonal#019-19741, Wako, 15 μg/ml; Mac-2/Gal3, Clone#M3/38, Cedarlane, 20 μg/ml; YKL-40, Clone#ab180569, Abcam, 30 μg/ml; S100b, Clone#EP1576Y, Abcam, 2 μg/ml; ApoJ/Clusterin, Clone#210, ThermoFisher, 40 μg/ml.

Validation

Antibodies were validated by respective manufactures and available on their website. Additional validations were also run before using the antibodies for Hyperion panel by IMC core at UCI

Animals and other research organisms

Policy information about studies involving animals; ARRIVE guidelines recommended for reporting animal research, and Sex and Gender in Research

Laboratory animals

C57BL/6J mouse, 5xFAD mouse model of AD harboring five familial AD mutations. 5xFAD hemizygous (C57BL16) and wildtype littermates were bred and housed until sacrifice at 4, 6, 8, and 12 months.

Wild animals

No wild animals were used in the study

Reporting on sex

Sex was considered throughout the study for both human and mouse studies. Sex specific differences were described in the results section

Field-collected samples

No field-collected samples were collected

Ethics oversight

All mouse work was approved by the Institutional Animal Care and Use (IACUC) committee at UCI.

Note that full information on the approval of the study protocol must also be provided in the manuscript.
